# Supplementary material for: A Time-to-Event Comparison of Immune and Endocrine Biomarkers and Latent Profiles in Hospitalisation: An Outcome-wide Approach
Source: medRxiv. 2026 Jan 11:2026.01.09.26343800. Preprint. [Version 1] doi: 10.64898/2026.01.09.26343800 (PMC12803293; doi:10.64898/2026.01.09.26343800)

Figure S1. Directed acyclic graph of *a priory* confounding

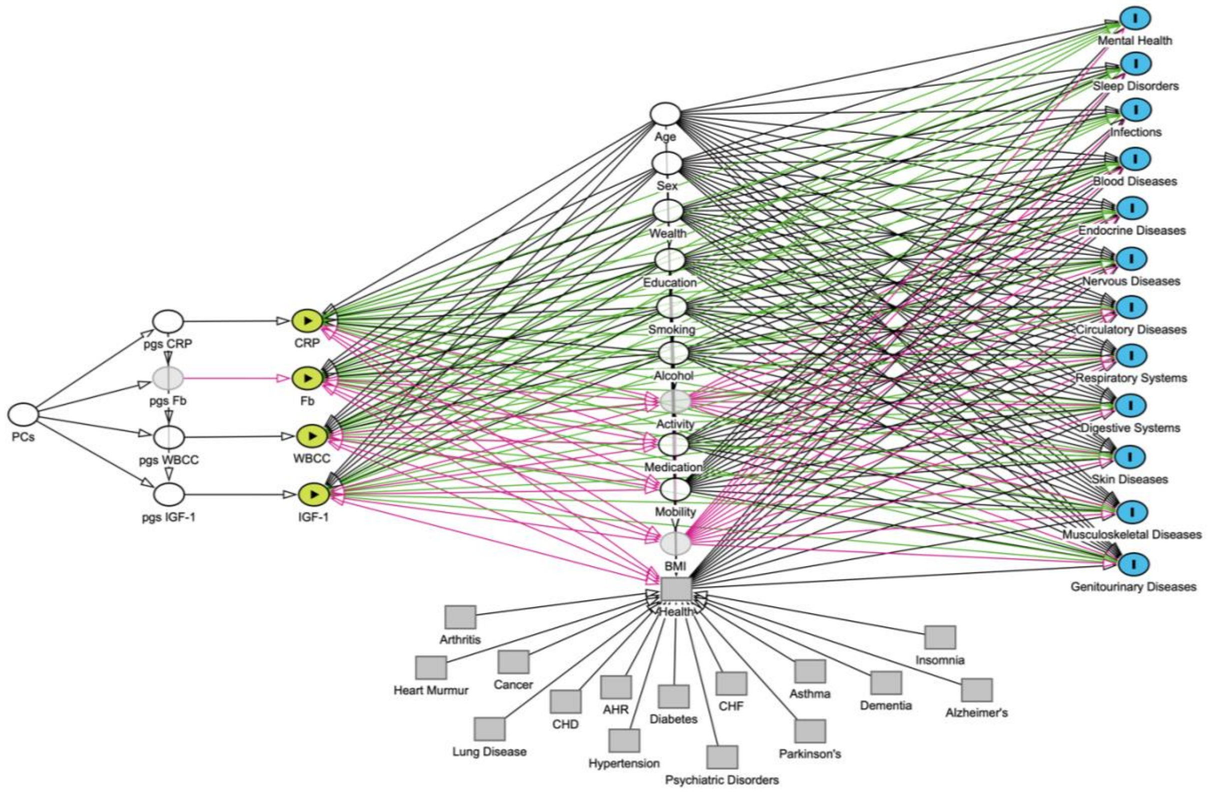

KEY

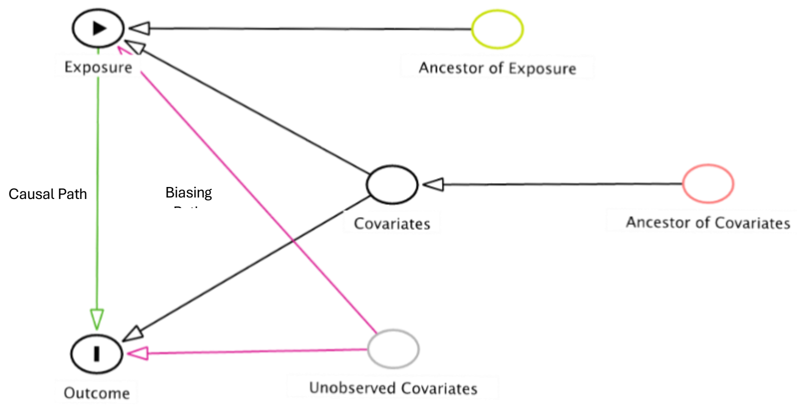

Figure S2. **Akaike Information Criterion (AIC) and Bayesian Information Criterion (BIC) Values of Immune and Neuroendocrine Profiles to Assess Model Fit**

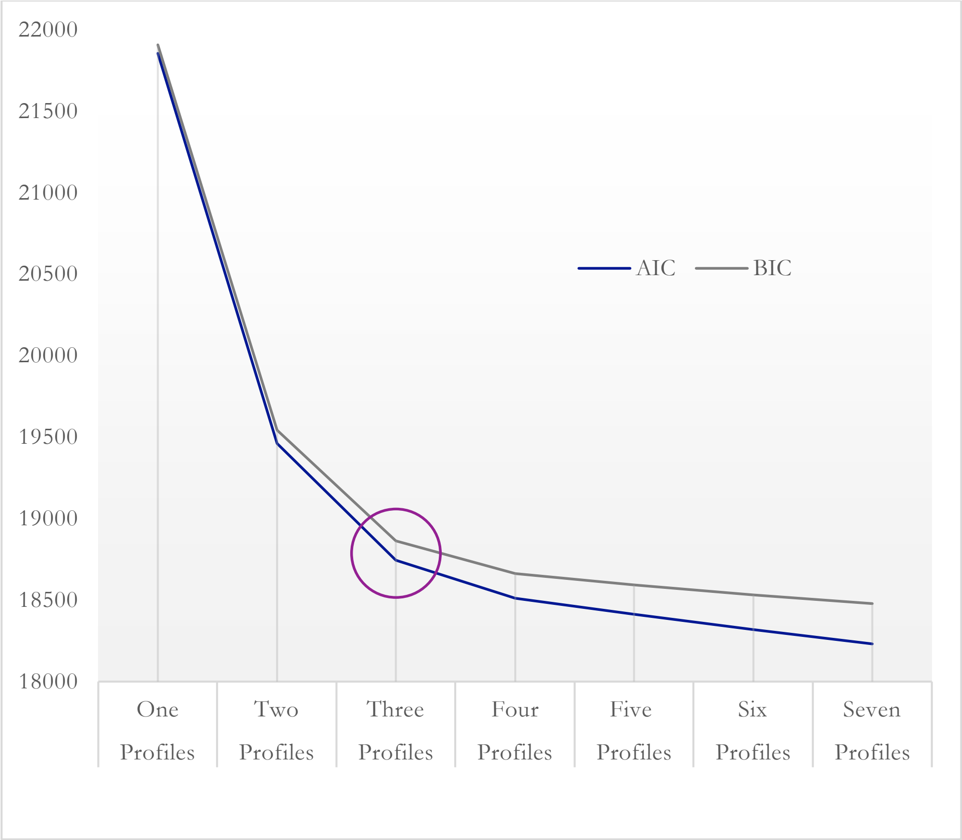

Figure S3. **Entropy and Normalised Entropy Values of Immune and Neuroendocrine Biomarker Profiles to Assess Profile Quality**

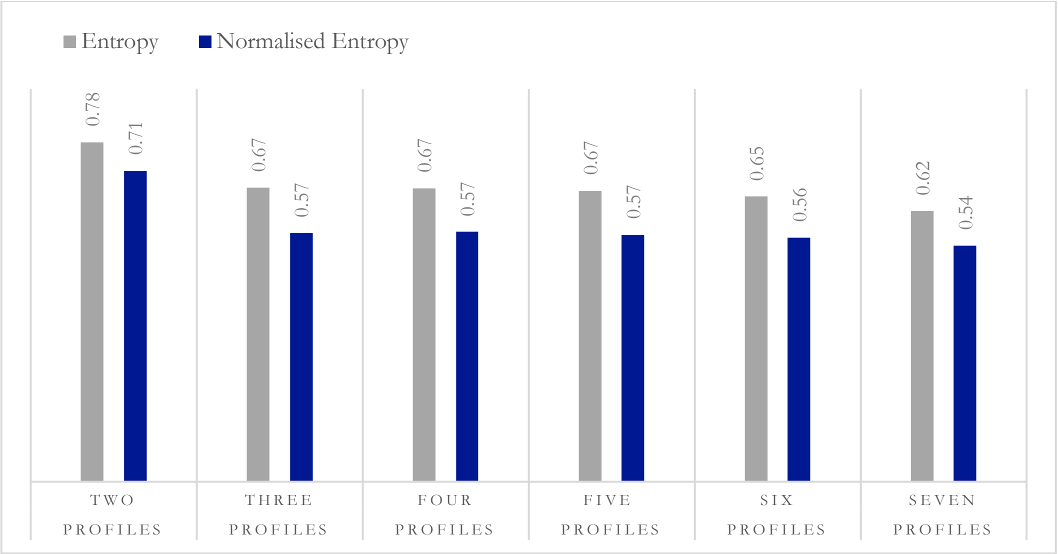

Figure S4. **Mean Posterior Probabilities of Immune and Neuroendocrine Biomarker Profiles to Assess Membership Confidence (≥5%)**

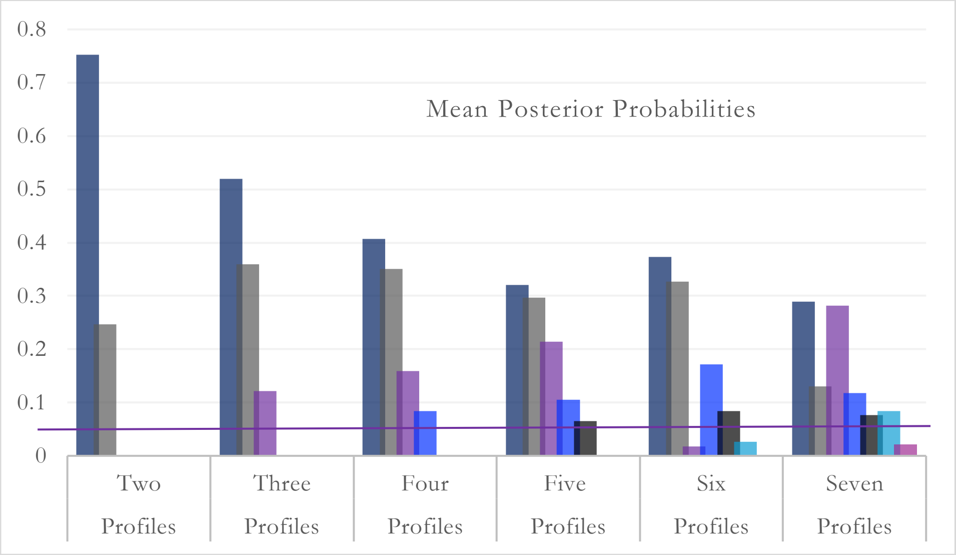

Figure S5 [a-g]. **Predicted mean of immune and neuroendocrine biomarker levels for a one to seven profile solution (N = 4,940)**

a

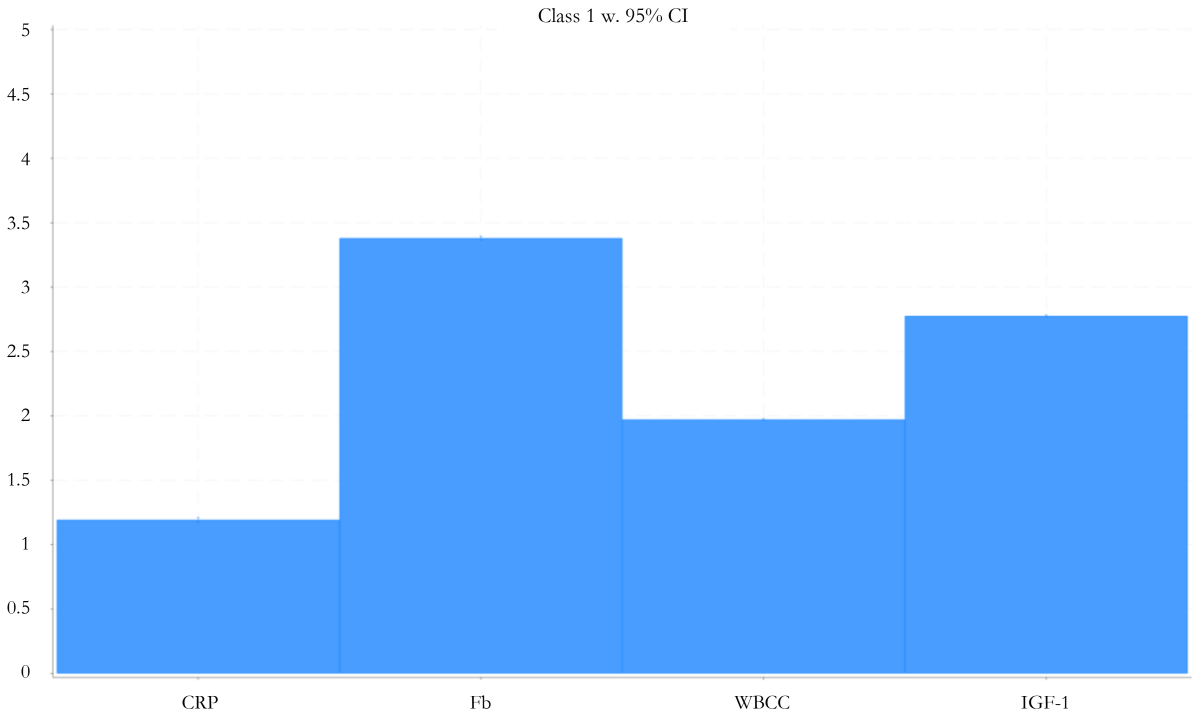

b

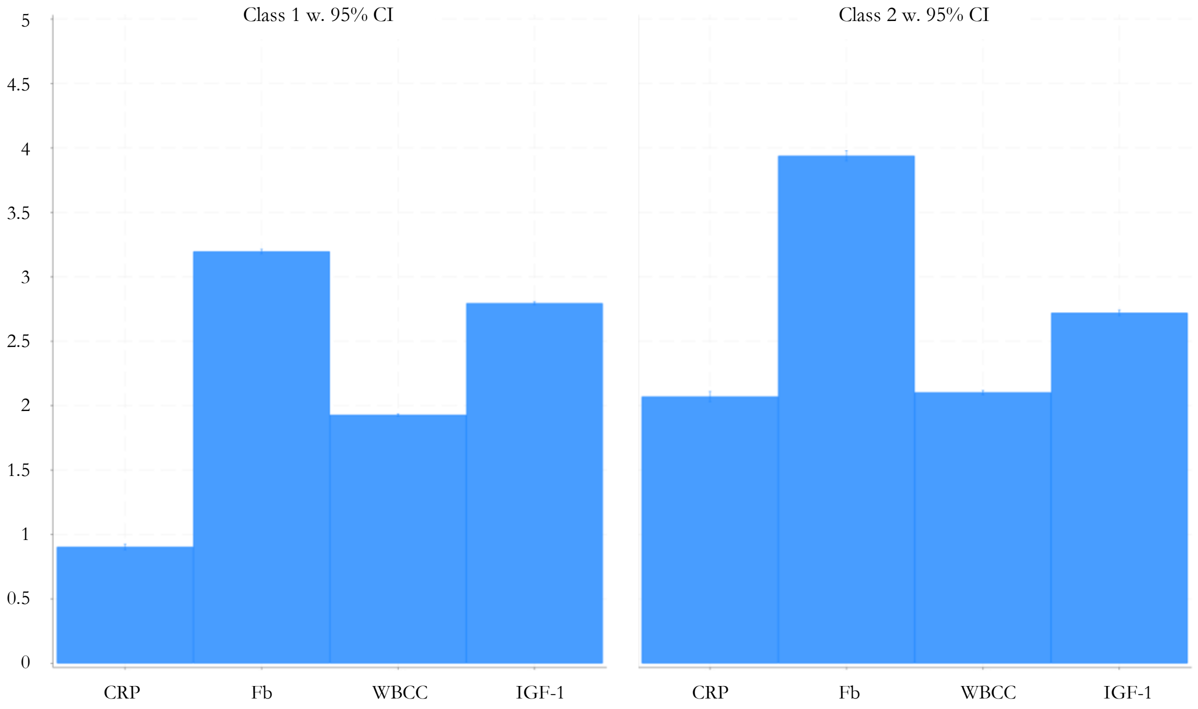

c

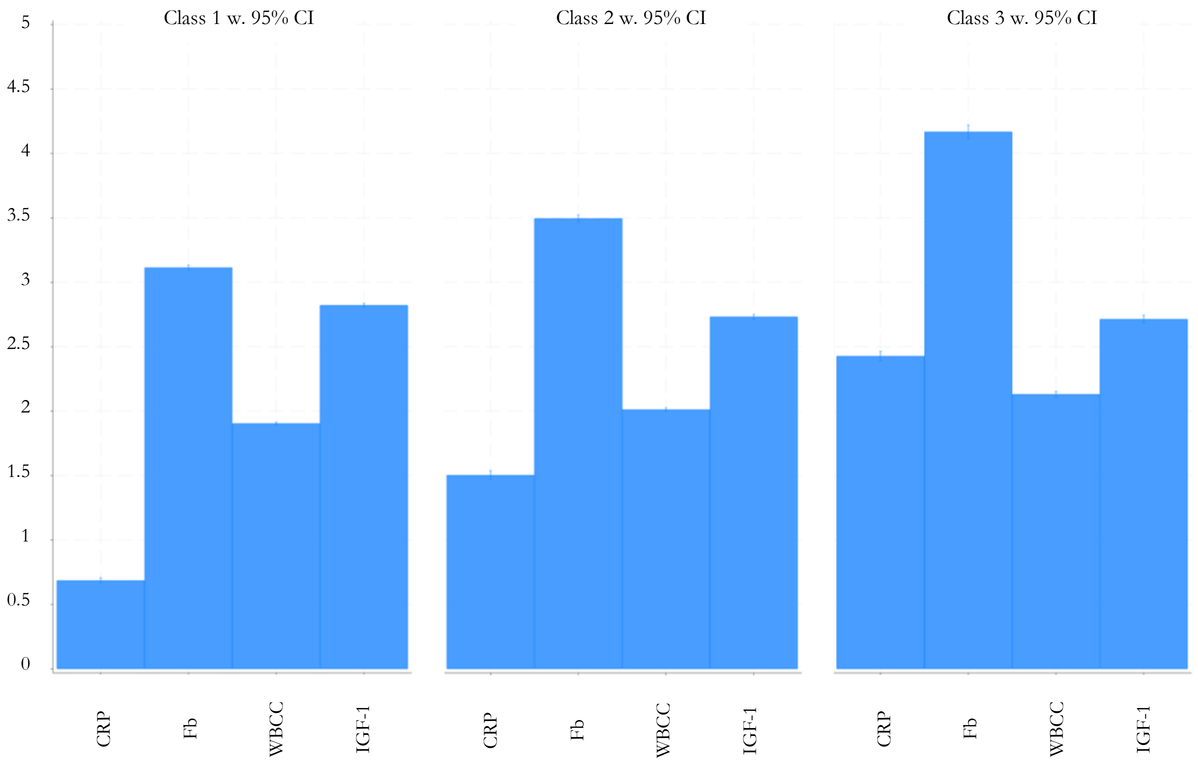

Class 1 w. 95% CI
Class 2 w. 95% CI
Class 3 w. 95% CI
5
4.5
4
3.5
3
2.5
2
1.5
1
0.5
0
CRP
Fb
WBCC
IGF-1

d

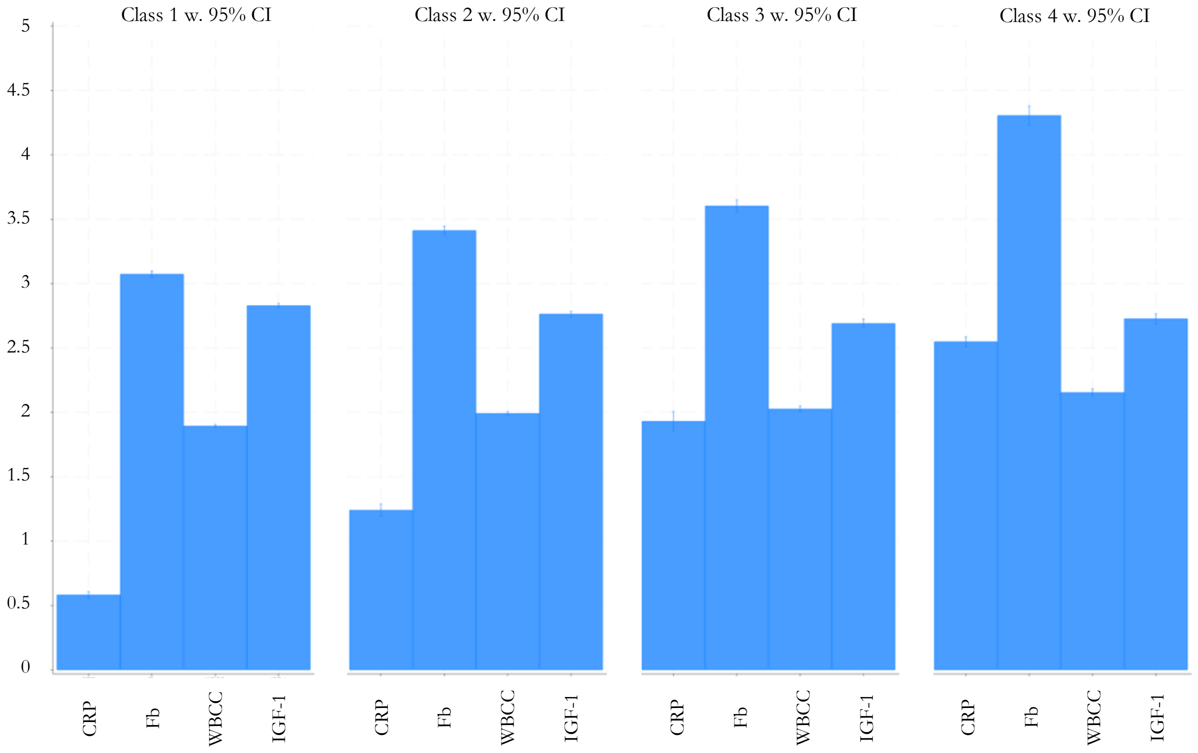

Class 1 w. 95% CI
Class 2 w. 95% CI
Class 3 w. 95% CI
Class 4 w. 95% CI
5
4.5
4
3.5
3
2.5
2
1.5
1
0.5
0
CRP
Fb
WBCC
IGF-1

e

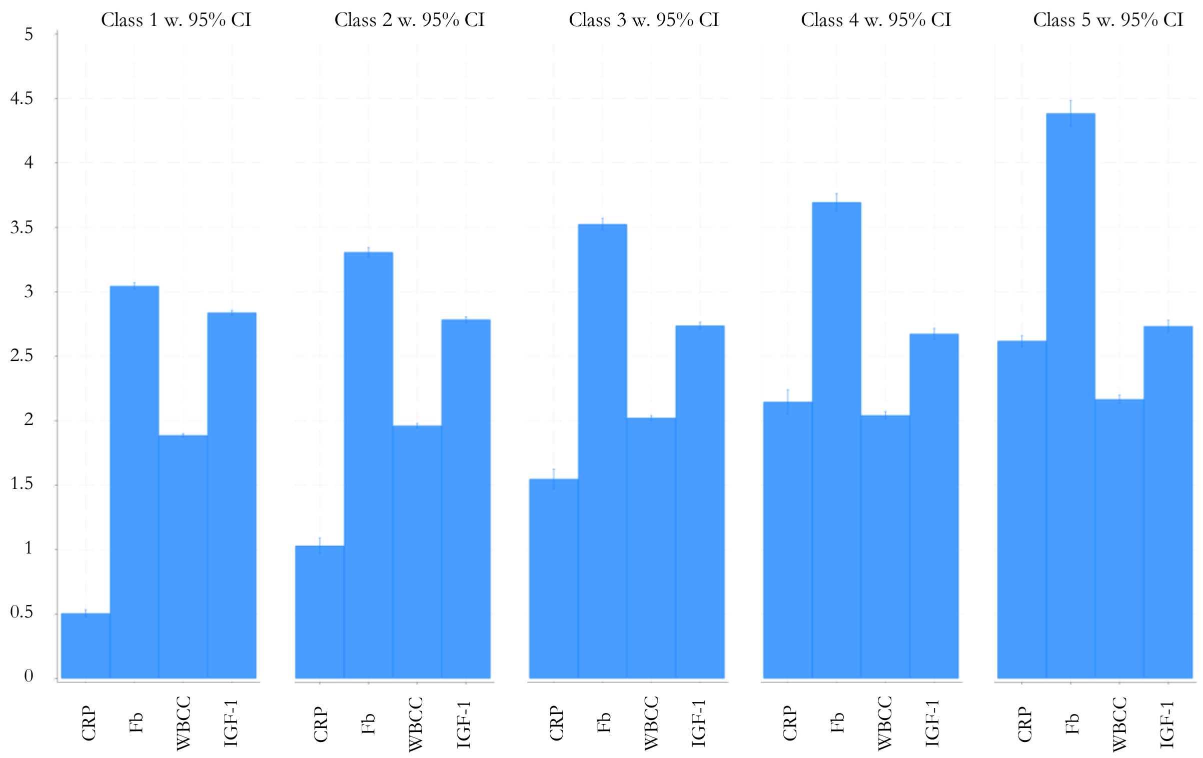
Class 1 w. 95% CI
Class 2 w. 95% CI
Class 3 w. 95% CI
Class 4 w. 95% CI
Class 5 w. 95% CI
5
4.5
4
3.5
3
2.5
2
1.5
1
0.5
0
CRP
Fb
WBCC
IGF-1

f

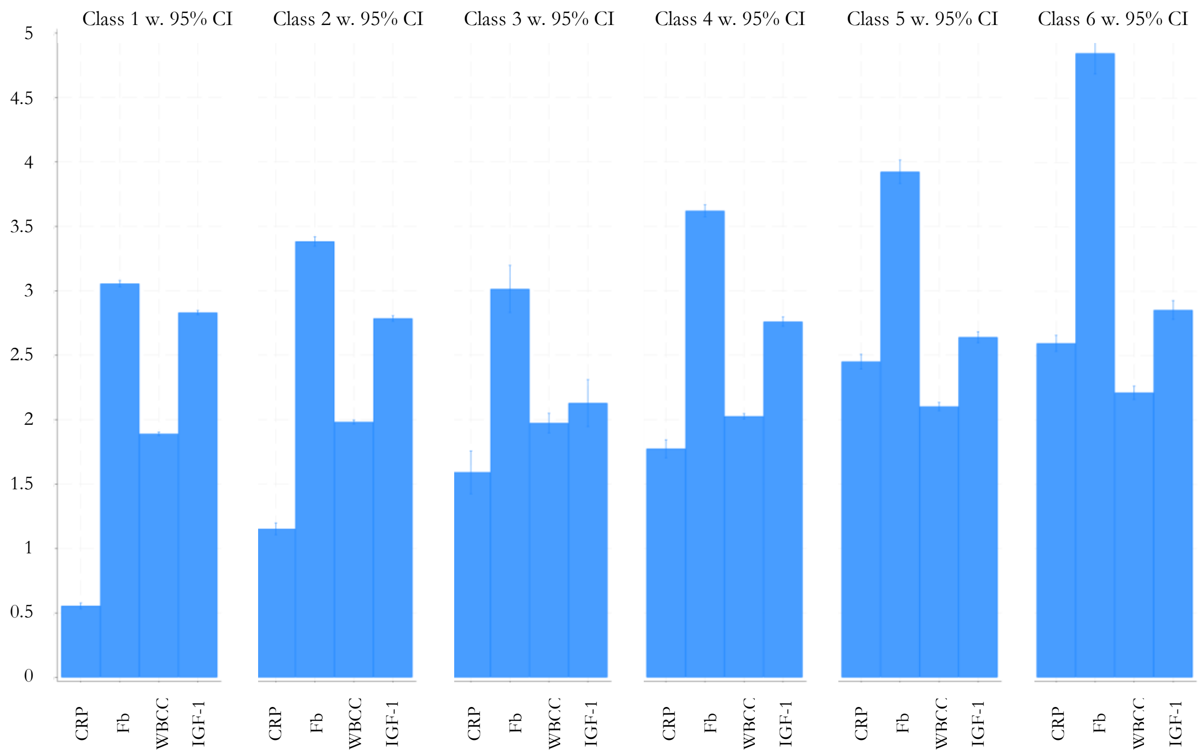
Class 1 w. 95% CI
Class 2 w. 95% CI
Class 3 w. 95% CI
Class 4 w. 95% CI
Class 5 w. 95% CI
Class 6 w. 95% CI
5
4.5
4
3.5
3
2.5
2
1.5
1
0.5
0
CRP
Fb
WBCC
IGF-1

g

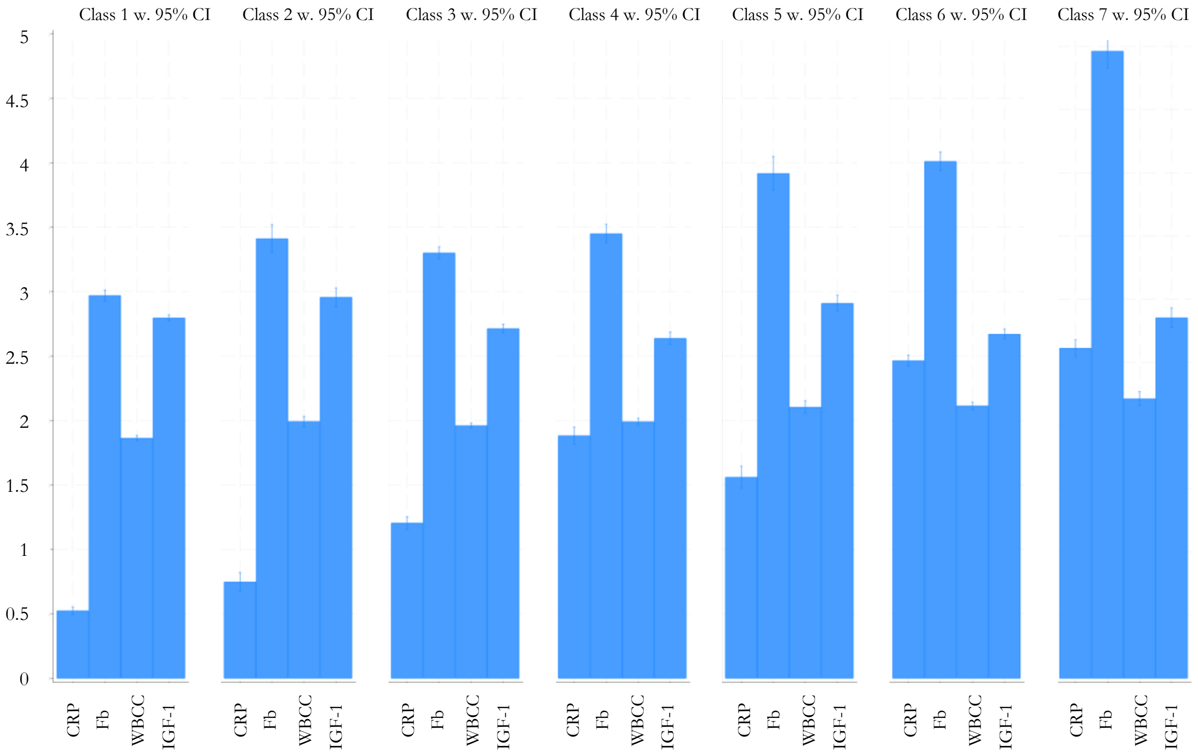
Class 1 w. 95% CI
Class 2 w. 95% CI
Class 3 w. 95% CI
Class 4 w. 95% CI
Class 5 w. 95% CI
Class 6 w. 95% CI
Class 7 w. 95% CI
5
4.5
4
3.5
3
2.5
2
1.5
1
0.5
0
CRP
Fb
WBCC
IGF-1

Figure S6. **The percentage of participants belonging to each immune and neuroendocrine biomarker profile with 95% confidence intervals for the wave 4 three-profile solution (N = 4,940)**

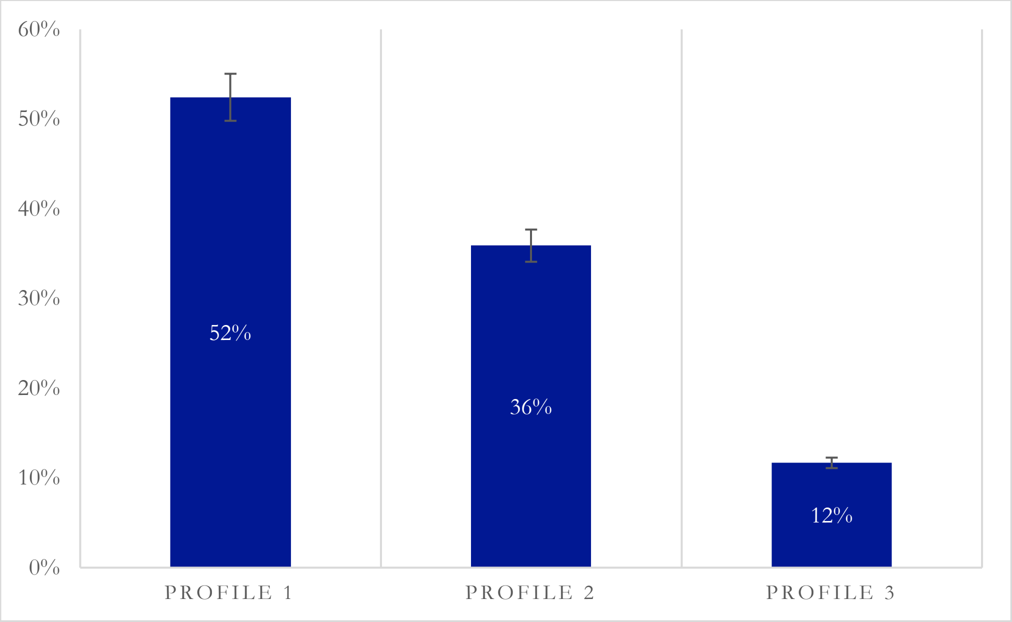

Profile	N	%
**1**	2,590	52.43
**2**	1,773	35.89
**3**	577	11.68

Figure S7. **Predicted Margins of Immune and Neuroendocrine Profiles**

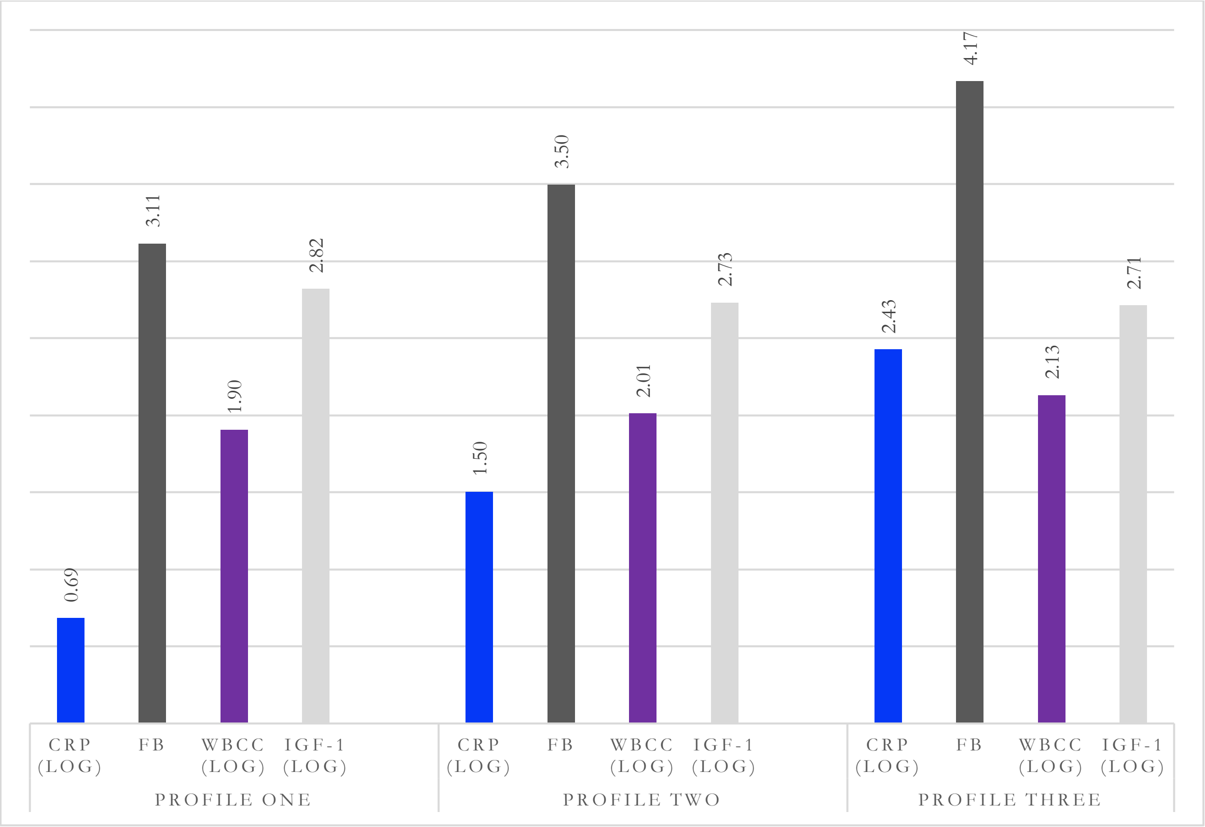

Supplement: Supplement 1 [file media-1.pdf]
