# Supplementary material for: A Time-to-Event Comparison of Immune and Endocrine Biomarkers and Latent Profiles in Hospitalisation: An Outcome-wide Approach
Source: medRxiv. 2026 Jan 11:2026.01.09.26343800. Preprint. [Version 1] doi: 10.64898/2026.01.09.26343800 (PMC12803293; doi:10.64898/2026.01.09.26343800)
Supplement: Supplement 3 [file media-3.pdf]

Table S1. **Diagnostic chapters, codes, and descriptions for hospitalisation episodes**

Chapter	Code	Description
**Chapter V** Psychiatric (mental and behavioural) disorders	F00-F09	Organic, including symptomatic, mental disorders
	F10-F19	Mental and behavioural disorders due to psychoactive substance use
	F20-F29	Schizophrenia, schizotypal and delusional disorders
	F30-F39	Mood [affective] disorders
	F40-F48	Neurotic, stress-related and somatoform disorders
	F50, F52-F59	Behavioural syndromes associated with physiological disturbances and physical factors
	F60-F69	Disorders of adult personality and behaviour
	F70-F79	Mental retardation
	F80-F89	Disorders of psychological development
	F90-F98	Behavioural and emotional disorders with onset usually occurring in childhood and adolescence
	F99-F99	Unspecified mental disorder
**Chapter V (F51) and Chapter VI (G47)** Sleep disorders	F51	Non-organic sleep disorders
	F51.0	Nonorganic insomnia
	F51.1	Nonorganic hypersomnia
	F51.2	Nonorganic disorder of the sleep-wake schedule
	F51.3	Sleepwalking [somnambulism]
	F51.4	Sleep terrors [night terrors]
	F51.5	Nightmares
	F51.8	Other nonorganic sleep disorders
	F51.9	Nonorganic sleep disorder, unspecified
	G47	Sleep disorders
	G47.0	Disorders of initiating and maintaining sleep [insomnias]
	G47.1	Disorders of excessive somnolence [hypersomnias]
	G47.2	Disorders of the sleep-wake schedule
	G47.3	Sleep apnoea
	G47.4	Narcolepsy and cataplexy
	G47.8	Other sleep disorders
	G47.9	Sleep disorder, unspecified
**Chapter I** Infectious and parasitic disorders	A00-A09	Intestinal infectious diseases
	A15-A19	Tuberculosis
	A20-A28	Certain zoonotic bacterial diseases
	A30-A49	Other bacterial diseases
	A50-A64	Infections with a predominantly sexual mode of transmission
	A65-A69	Other spirochaetal diseases
	A70-A74	Other diseases caused by chlamydiae
	A75-A79	Rickettsioses
	A80-A89	Viral infections of the central nervous system
	A92-A99	Arthropod-borne viral fevers and viral haemorrhagic fevers
	B00-B09	Viral infections characterized by skin and mucous membrane lesions
	B15-B19	Viral hepatitis
	B20-B24	Human immunodeficiency virus [HIV] disease
	B25-B34	Other viral diseases
	B35-B49	Mycoses
	B50-B64	Protozoal diseases

	B65-B83	Helminthiases
	B85-B89	Pediculosis, acariasis and other infestations
	B90-B94	Sequelae of infectious and parasitic diseases
	B95-B98	Bacterial, viral and other infectious agents
	B99-B99	Other infectious diseases
**Chapter III** Disorders of the blood and blood-forming organs and certain disorders involving the immune mechanism	D50-D53	Nutritional anaemias
	D55-D59	Haemolytic anaemias
	D60-D64	Aplastic and other anaemias
	D65-D69	Coagulation defects, purpura and other haemorrhagic conditions
	D70-D77	Other diseases of blood and blood-forming organs
	D80-D89	Certain disorders involving the immune mechanism
**Chapter IV** Endocrine, nutritional and metabolic disorders	E00-E07	Disorders of thyroid gland
	E10-E14	Diabetes mellitus
	E15-E16	Other disorders of glucose regulation and pancreatic internal secretion
	E20-E35	Disorders of other endocrine glands
	E40-E46	Malnutrition
	E50-E64	Other nutritional deficiencies
	E65-E68	Obesity and other hyperalimentation
	E70-E90	Metabolic disorders
**Chapter VI** Disorders of the nervous system	G00-G09	Inflammatory diseases of the central nervous system
	G10-G14	Systemic atrophies primarily affecting the central nervous system
	G20-G26	Extrapyramidal and movement disorders
	G30-G32	Other degenerative diseases of the nervous system
	G35-G37	Demyelinating diseases of the central nervous system
	G40-G46	Episodic and paroxysmal disorders
	G50-G59	Nerve, nerve root and plexus disorders
	G60-G64	Polyneuropathies and other disorders of the peripheral nervous system
	G70-G73	Diseases of myoneural junction and muscle
	G80-G83	Cerebral palsy and other paralytic syndromes
	G90-G99	Other disorders of the nervous system
**Chapter IX** Disorders of the circulatory system	I00-I02	Acute rheumatic fever
	I05-I09	Chronic rheumatic heart diseases
	I10-I15	Hypertensive diseases
	I20-I25	Ischaemic heart diseases
	I26-I28	Pulmonary heart disease and diseases of pulmonary circulation
	I30-I52	Other forms of heart disease
	I60-I69	Cerebrovascular diseases
	I70-I79	Diseases of arteries, arterioles and capillaries
	I80-I89	Diseases of veins, lymphatic vessels and lymph nodes, not elsewhere classified
	I95-I99	Other and unspecified disorders of the circulatory system
**Chapter X** Disorders of the respiratory system	J00-J06	Acute upper respiratory infections
	J09-J18	Influenza and pneumonia
	J20-J22	Other acute lower respiratory infections
	J30-J39	Other diseases of upper respiratory tract
	J40-J47	Chronic lower respiratory diseases
	J60-J70	Lung diseases due to external agents
	J80-J84	Other respiratory diseases principally affecting the interstitium

	J85-J86	Suppurative and necrotic conditions of lower respiratory tract
	J90-J94	Other diseases of pleura
	J95-J99	Other diseases of the respiratory system
**Chapter XI** Disorders of the digestive system	K00-K14	Diseases of oral cavity, salivary glands and jaws
	K20-K31	Diseases of oesophagus, stomach and duodenum
	K35-K38	Diseases of appendix
	K40-K46	Hernia
	K50-K52	Noninfective enteritis and colitis
	K55-K64	Other diseases of intestines
	K65-K67	Diseases of peritoneum
	K70-K77	Diseases of liver
	K80-K87	Disorders of gallbladder, biliary tract and pancreas
	K90-K93	Other diseases of the digestive system
**Chapter XII** Disorders of the skin and subcutaneous tissue	L00-L08	Infections of the skin and subcutaneous tissue
	L10-L14	Bullous disorders
	L20-L30	Dermatitis and eczema
	L40-L45	Papulosquamous disorders
	L50-L54	Urticaria and erythema
	L55-L59	Radiation-related disorders of the skin and subcutaneous tissue
	L60-L75	Disorders of skin appendages
	L80-L99	Other disorders of the skin and subcutaneous tissue
**Chapter XIII** Disorders of the musculoskeletal system and connective tissue	M00-M03	Infectious arthropathies
	M05-M14	Inflammatory polyarthropathies
	M15-M19	Arthrosis
	M20-M25	Other joint disorders
	M30-M36	Systemic connective tissue disorders
	M40-M43	Deforming dorsopathies
	M45-M49	Spondylopathies
	M50-M54	Other dorsopathies
	M60-M63	Disorders of muscles
	M65-M68	Disorders of synovium and tendon
	M70-M79	Other soft tissue disorders
	M80-M85	Disorders of bone density and structure
	M86-M90	Other osteopathies
	M91-M94	Chondropathies
	M95-M99	Other disorders of the musculoskeletal system and connective tissue
**Chapter XIV** Disorders of the genitourinary system	N00-N08	Glomerular diseases
	N10-N16	Renal tubulo-interstitial diseases
	N17-N19	Renal failure
	N20-N23	Urolithiasis
	N25-N29	Other disorders of kidney and ureter
	N30-N39	Other diseases of urinary system
	N40-N51	Diseases of male genital organs
	N60-N64	Disorders of breast
	N70-N77	Inflammatory diseases of female pelvic organs
	N80-N98	Noninflammatory disorders of female genital tract
	N99-N99	Other disorders of the genitourinary system

Table S2. **Comparison of sample characteristics**

		CHAPTERS												
**Variables**		**ELSA**	**V**	**V (F51) & VI (G47)**	**III**	**IX**	**XI**	**IV**	**XIV**	**I**	**XIII**	**VI**	**X**	**XII**
		n **(%) | M ±**	***n*** **(%) | M ±**	***n*** **(%) | M ±**	***n*** **(%) | M ±**	***n*** **(%) | M ±**	***n*** **(%) | M ±**	***n*** **(%) | M ±**	***n*** **(%) | M ±**	***n*** **(%) | M ±**	***n*** **(%) | M ±**	***n*** **(%) | M ±**	***n*** **(%) | M ±**	***n*** **(%) | M ±**
		4,940	4,181	4,276	4,139	3,163	3,188	3,749	3,524	4,118	3,441	4,057	3,846	4,045
Age		66.3 ±9.4	66.7 ±9.3	66.7 ±9.3	66.5 ±9.3	65.3 ±8.7	65.8 ±9.2	66.2 ±9.2	66.5 ±9.3	66.5 ±9.3	66.1 ±9.2	66.5 ±9.3	66.4 ±9.2	66.7 ±9.3
Age (Binary)	< Md	2,436 (49.31)	2,007 (48.00)	2047 (47.87)	2,008 (48.51)	1,713 (54.16)	1,667 (52.29)	1,884 (50.25)	1,721 (48.84)	1,990 (48.32)	1,724 (50.10)	1,957 (48.24)	1,884 (48.99)	1,949 (48.18)
	≥ Md	2,504 (50.69)	2,174 (52.00)	2229 (52.13)	2,131 (51.49)	1,450 (45.84)	1,521 (47.71)	1,865 (49.75)	1,803 (51.16)	2,128 (51.68)	1,717 (49.90)	2,100 (51.76)	1,962 (51.01)	2,096 (51.82)
Sex	Male	2,237 (45.28)	1,904 (45.54)	1,949 (45.58)	1,897 (45.83)	1,404 (44.39)	1,428 (44.79)	1,720 (45.88)	1,694 (48.07)	1,889 (45.87)	1,595 (46.35)	1,864 (45.95)	1,749 (45.48)	1,857 (45.91)
	Female	2,703 (54.72)	2,277 (54.46)	2,327 (54.42)	2,242 (54.17)	1,759 (55.61)	1,760 (55.21)	2,029 (54.12)	1,830 (51.93)	2,229 (54.13)	1,846 (53.65)	2,193 (54.05)	2,097 (54.52)	2,188 (54.09)
Education	Higher	1,589 (32.17)	1,370 (32.77)	1,388 (32.46)	1,364 (32.95)	1,099 (34.75)	1,084 (34.00)	1,265 (33.74)	1,181 (33.51)	1,357 (32.95)	1,157 (33.62)	1,325 (32.66)	1,294 (33.65)	1,314 (32.48)
	Primary/ Secondary/ Tertiary	1,543 (31.23)	1,303 (31.16)	1,335 (31.22)	1,286 (31.07)	1,013 (32.03)	1,019 (31.96)	1,190 (31.74)	1,108 (31.44)	1,285 (31.20)	1,095 (31.82)	1,270 (31.30)	1,196 (31.10)	1,273 (31.47)
	Alternative/ None	1,808 (36.60)	1,508 (36.07)	1,553 (36.32)	1,489 (35.97)	1,051 (33.23)	1,019 (31.96)	1,294 (34.52)	1,235 (35.05)	1,476 (35.84)	1,189 (34.55)	1,462 (36.04)	1,356 (35.26)	1,458 (36.04)
Wealth	Lowest	1,573 (31.84)	1,388 (33.20)	1,448 (33.86)	1,383 (33.41)	976 (30.86)	1,010 (31.68)	1,201 (32.04)	1,165 (33.06)	1,373 (33.34)	1,082 (31.44)	1,347 (33.20)	1,237 (32.16)	1,357 (33.55)
	Middle	2,014 (40.77)	1,753 (41.93)	1,786 (41.77)	1,732 (41.85)	1,343 (42.46)	1,339 (42.00)	1,583 (42.22)	1,470 (41.71)	1,719 (41.74)	1,458 (42.37)	1,709 (42.12)	1,634 (42.49)	1,692 (41.83)
	Highest	1,353 (27.39)	1,040 (24.87)	1,042 (24.37)	1,024 (24.74)	844 (26.68)	839 (26.32)	965 (25.74)	889 (25.23)	1,026 (24.92)	901 (26.18)	1,001 (24.67)	975 (25.35)	996 (24.62)
Smoking Status	Never/ Ex-Smokers	4,312 (87.29)	3,673 (87.85)	3,736 (87.37)	3,614 (87.32)	2,744 (86.75)	2,768 (86.83)	3,278 (87.44)	3,082 (87.46)	3,596 (87.32)	3,005 (87.33)	3,545 (87.38)	3,383 (87.96)	3,545 (87.64)
	Current Smoker	628 (12.71)	508 (12.15)	540 (12.63)	525 (12.68)	419 (13.25)	420 (13.17)	471 (12.56)	442 (12.54)	522 (12.68)	436 (12.67)	512 (12.62)	463 (12.04)	500 (12.36)
Alcohol	<3 days a week	3,175 (64.27)	2,680 (64.10)	2,748 (64.27)	2,642 (63.83)	1,977 (62.50)	2,013 (63.14)	2,364 (63.06)	2,219 (62.97)	2,633 (63.94)	2,161 (62.80)	2,582 (63.64)	2,446 (63.60)	2,589 (64.00)
Consumption	≥3 days a week	1,765 (35.73)	1,501 (35.90)	1,528 (35.73)	1,497 (36.17)	1,186 (37.50)	1,175 (36.86)	1,385 (36.94)	1,305 (37.03)	1,485 (36.06)	1,280 (37.20)	1,475 (36.36)	1,400 (36.40)	1,456 (36.00)
Mobility	Mobile	2,678 (54.21)	2,291 (54.80)	2,363 (55.26)	2,252 (54.41)	1,534 (48.50)	1,665 (52.23	1,952 (52.07)	1,894 (53.75)	2,240 (54.40)	1,708 (49.64)	2,182 (53.78)	2,029 (52.76)	2,202 (54.44)
	Not Mobile	2,262 (45.79)	1,890 (45.20)	1,913 (44.74)	1,887 (45.59)	1,629 (51.50)	1,523 (47.77)	1,797 (47.93)	1,630 (46.25)	1,878 (45.60)	1,733 (50.36)	1,875 (46.22)	1,817 (47.24)	1,843 (45.56)
Medication	Not Medicated	4,887 (98.93)	4,139 (99.00)	4,230 (98.92)	4,098 (99.01)	3,142 (99.34)	3,163 (99.22)	3,720 (99.23)	3,487 (98.95)	4,074 (98.93)	3,415 (99.24)	4,020 (99.09)	3,814 (99.17)	4,003 (98.96)
	Medicated	53 (1.07)	42 (1.00)	46 (1.08)	41 (0.99)	21 (0.66)	25 (0.78)	29 (0.77)	37 (1.05)	44 (1.07)	26 (0.76)	37 (0.91)	32 (0.83)	42 (1.04)
Physical Activity	Sedentary	1,340 (27.13)	1,120 (26.79)	1,169 (27.34)	1,109 (26.79)	737 (23.30)	785 (24.62)	941 (25.10)	922 (26.16)	1,094 (26.57)	837 (24.32)	1,079 (26.60)	970 (25.22)	1,079 (26.67)
	Active	3,600 (72.87)	3,061 (73.21)	3,107 (72.66)	3,030 (73.21)	2,426 (76.70)	2,403 (75.38)	2,808 (74.90)	2,602 (73.84)	3,024 (73.43)	2,604 (75.68)	2,978 (73.40)	2,876 (74.78)	2,966 (73.33)
BMI	<25/ Normal	1,312 (26.56)	1,085 (25.95)	1,110 (25.96)	1,070 (25.85)	894 (28.26)	879 (27.57)	1,013 (27.02)	933 (26.48)	1,080 (26.23)	950 (27.61)	1,076 (26.52)	1,009 (26.24)	1,057 (26.13)
	25-30, Overweight: Pre-obese	2,213 (44.80)	1,877 (44.89)	1,919 (44.88)	1,866 (45.08)	1,432 (45.27)	1,393 (43.70)	1,692 (45.13)	1,581 (44.86)	1,844 (44.78)	1,552 (45.10)	1,813 (44.69)	1,742 (45.29)	1,822 (45.04)
	≥30, Obese	1,415 (28.64)	1,219 (29.16)	1,247 (29.16)	1,203 (29.06)	837 (26.46)	916 (28.73)	1,044 (27.85)	1,010 (28.66)	1,194 (28.99)	939 (27.29)	1,168 (28.79)	1,095 (28.47)	1,166 (28.83)
Health	No health condition	3,319 (67.19)	2,754 (65.87)	2,809 (65.69)	2,744 (66.30)	2,245 (70.98)	2,216 (69.51)	2,567 (68.47)	2,369 (67.22)	2,732 (66.34)	2,421 (70.36)	2,705 (66.67)	2,629 (68.36)	2,685 (66.38)
	At least one health condition	1,621 (32.81)	1,427 (34.13)	1,467 (34.31)	1,395 (33.70)	918 (29.02)	972 (30.49)	1,182 (31.53)	1,155 (32.78)	1,386 (33.66)	1,020 (29.64)	1,352 (33.33)	1,217 (31.64)	1,360 (33.62)
CRP* (mg/L)		1.2 ±0.7	1.2 ±0.7	1.2 ±0.7	1.2 ±0.7	1.2 ±0.7	1.2 ±0.7	1.2 ±0.7	1.2 ±0.7	1.2 ±0.7	1.2 ±0.7	1.2 ±0.7	1.2 ±0.7	1.2 ±0.7
Fb (g/L)		3.4 ±0.6	3.4 ±0.6	3.4 ±0.6	3.4 ±0.6	3.4 ±0.5	3.4 ±0.6	3.4 ±0.6	3.4 ±0.6	3.4 ±0.6	3.4 ±0.6	3.4 ±0.6	3.4 ±0.5	3.4 ±0.6
WBCC* (nmol/L)		2.0 ±0.3	2.0 ±0.3	2.0 ±0.3	2.0 ±0.3	2.0 ±0.3	2.0 ±0.3	2.0 ±0.3	2.0 ±0.3	2.0 ±0.3	2.0 ±0.3	2.0 ±0.3	2.0 ±0.3	2.0 ±0.3
IGF-1* (nmol/L)		2.8 ±0.3	2.8 ±0.3	2.8 ±0.3	2.8 ±0.3	2.8 ±0.3	2.8 ±0.3	2.8 ±0.3	2.8 ±0.3	2.8 ±0.3	2.8 ±0.3	2.8 ±0.3	2.8 ±0.3	2.8 ±0.3
I-N Profiles	*Low-risk*	2,590 (52.43)	2,199 (52.60)	2,234 (52.25)	2,176 (52.57)	1,739 (54.98)	1,728 (54.20)	1,994 (53.19)	1,897 (53.83)	2,176 (52.84)	1,887 (54.84)	2,128 (52.45)	2,075 (53.95)	2,145 (53.03)
	Moderate-risk	1,773 (35.89)	1,493 (35.71)	1,535 (35.90)	1,481 (35.78)	1,091 (34.49)	1,113 (34.91)	1,330 (35.48)	1,234 (35.02)	1,467 (35.62)	1,189 (34.55)	1,453 (35.81)	1,356 (35.26)	1,441 (35.62)
	High-risk	577 (11.68)	489 (11.70)	507 (11.86)	482 (11.65)	333 (10.53)	347 (10.88)	425 (11.34)	393 (11.15)	475 (11.53)	365 (10.61)	476 (11.73)	415 (10.79)	459 (11.35)

Notes: ELSA = English Longitudinal Study of Ageing; Chapter V = Mental and behavioural disorders; Chapter V (F51) and Chapter VI (G47) = Sleep disorders; Chapter III = Diseases of the blood and blood-forming organs and certain disorders involving the immune mechanism; Chapter IX = Diseases of the circulatory system; Chapter XI = Diseases of the digestive system; Chapter IV = Endocrine, nutritional, and metabolic diseases; Chapter XIV = Diseases of the genitourinary system; Chapter I = Infectious and parasitic diseases; Chapter XIII = Diseases of the musculoskeletal system and connective tissue; Chapter VI = Diseases of the nervous system; Chapter X = Diseases of the respiratory system; Chapter XII = Diseases of the skin and subcutaneous tissue; *n* = observations; M = Mean; Md = Median; % = percentage frequencies; ± = standard deviations; < = less than; ≥ = greater than or equal to; BMI = Body Mass Index; PGS = Polygenic Score; CRP = C-reactive protein; Fb = Fibrinogen; WBCC = White Blood Cell Counts; IGF-1 = Insulin-growth factor-1; * Log-transformed variable; I-N = Immune and Neuroendocrine.

Table S3. **Sample size and size difference, along with the number of failures and time to risk.**

Disorder Chapter	*n*	Difference	Failures	Time at Risk
**ELSA Sample**	**4,940**	-	-	-
**Chapter V (F51) and Chapter VI (G47)** Sleep disorders	4,276	664	49	36,019.76
**Chapter V** Psychiatric (mental and behavioural) disorders	4,181	759	740	33,316.10
**Chapter III** Diseases of the blood and blood-forming organs and certain disorders involving the immune mechanism	4,139	801	449	33,870.95
**Chapter I** Infectious and parasitic diseases	4,118	822	553	33,474.10
**Chapter VI** Diseases of the nervous system	4,057	883	539	32,794.69
**Chapter XII** Diseases of the skin and subcutaneous tissue	4,045	895	472	32,794.12
**Chapter X** Diseases of the respiratory system	3,846	1,094	896	30,151.82
**Chapter IV** Endocrine, nutritional, and metabolic diseases	3,749	1,191	1,174	27,624.52
**Chapter XIV** Diseases of the genitourinary system	3,524	1,416	906	26,907.90
**Chapter XIII** Diseases of the musculoskeletal system and connective tissue	3,441	1,499	1,080	25,155.85
**Chapter XI** Diseases of the digestive system	3,188	1,752	1,155	22,686.37
**Chapter IX** Diseases of the circulatory system	3,163	1,777	1,406	21,543.72

Table S4. **Correlations between biomarkers and polygenic scores**

	CRP	Fb	WBCC	IGF-1	PGS CRP	PGS WBCC	PGS IGF-1	PGS Anxiety	PGS Depression	PGS Schizophrenia	PGS Insomnia	PGS Pain
**CRP**	1											
**Fb**	**0.567***	1										
	<0.001											
**WBCC**	**0.301***	**0.245***	1									
	<0.001	*<0.001*										
**IGF-1**	**-0.164***	**0.051***	0.003	1								
	<0.001	*0.001*	*0.831*									
**PGS CRP**	**0.043***	-0.005	0.006	**-0.035***	1							
	0.005	*0.734*	*0.674*	*0.024*								
**PGS WBCC**	0.027	-0.009	**0.085***	0.024	0.001	1						
	0.078	*0.556*	*<0.001*	*0.111*	*0.972*							
**PGS IGF-1**	0.018	**0.034***	0.004	0.023	-0.019	0.018	1					
	0.242	*0.026*	*0.803*	*0.126*	*0.207*	*0.234*						
**PGS Anxiety**	0.004	-0.002	0.027	0.002	-0.011	**0.578***	0.014	1				
	0.786	*0.883*	*0.073*	*0.915*	*0.476*	*<0.001*	*0.349*					
**PGS Depression**	0.029	0.008	**0.047***	-0.003	-0.021	**0.273***	0.008	**0.213***	1			
	0.061	*0.584*	*0.002*	*0.861*	*0.164*	*<0.001*	*0.623*	*<0.001*				
**PGS Schizophrenia**	0.025	0.006	**0.040***	0.022	-0.023	**0.789***	0.008	**0.557***	**0.275***	1		
	0.101	*0.679*	*0.009*	*0.151*	*0.126*	*<0.001*	*0.619*	*<0.001*	*<0.001*			
**PGS Insomnia**	-0.006	0.019	0.002	0.013	0.024	**-0.032***	-0.029	**-0.103***	**-0.037***	**-0.124***	1	
	0.698	*0.224*	*0.906*	*0.386*	*0.124*	*0.036*	*0.058*	*<0.001*	*0.015*	*<0.001*		
**PGS Pain**	**0.075***	**0.059***	**0.054***	-0.004	-0.020	**0.215***	**-0.031***	**0.167***	**0.267***	**0.214***	**0.043***	1
	<0.001	*<0.001*	*<0.001*	*0.777*	*0.202*	*<0.001*	*0.040*	*<0.001*	*<0.001*	*<0.001*	*0.005*	

Notes: PGS = Polygenic Score; CRP = C-reactive protein; Fb = Fibrinogen; WBCC = White Blood Cell Counts; IGF-1 = Insulin-growth factor-1.

Table S5. **Latent Profile Analysis**

	**One Profile**	**Two Profiles**	**Three Profiles**	**Four Profiles**	**Five Profiles**	**Six Profiles**	**Seven Profiles**
AIC	21856.03	19459.96	18745.36	18512.47	18412.80	18318.50	18231.59
AIC Difference (N)	-	2396.07	714.60	232.89	99.67	94.30	86.91
AIC Difference (%)	-	12.31	3.81	1.26	0.54	0.51	0.48
BIC	21908.07	19544.53	18862.45	18662.08	18594.94	18533.17	18478.78
BIC Difference (N)	-	2363.54	682.08	200.37	67.14	61.77	54.39
BIC Difference (%)	-	12.09	3.62	1.07	0.36	0.33	0.29
aBIC	21882.65	19503.22	18805.26	18589.00	18505.97	18428.31	18358.03
aBIC Difference (N)	-	2379.43	697.97	216.26	83.03	77.66	70.28
aBIC Difference (%)	-	12.20	3.71	1.16	0.45	0.42	0.38
Entropy	-	0.78	0.67	0.67	0.67	0.65	0.62
Normalised Entropy	-	0.71	0.57	0.57	0.57	0.56	0.54
M Posterior Probabilities (SE)	-	0.753 (.010)	0.519 (.012)	0.407 (.014)	0.320 (.016)	0.373 (.014)	0.289 (.025)
		0.247 (.010)	0.359 (.011)	0.350 (.011)	0.297 (.013)	0.327 (.012)	0.130 (.031)
			0.122 (.007)	0.159 (.011)	0.214 (.013)	0.017 (.006)	0.282 (.019)
				0.084 (.007)	0.105 (.009)	0.172 (.011)	0.118 (.013)
					0.065 (.007)	0.084 (.007)	0.076 (.017)
						0.026 (.005)	0.084 (.006)
							0.021 (.004)
N classes >5%	Yes	Yes	Yes	Yes	Yes	No	No

Table S6. **Latent Profile Predicted Means and Percentage Point Change**

		Profile 1	Profile 2	Profile 3
Predicted Means	CRP*	0.69	1.5	2.43
	**% Change**	-	**54%**	**38%**
	Fb	3.11	3.5	4.17
	**% Change**	-	**11%**	**16%**
	WBCC*	1.9	2.01	2.13
	**% Change**	-	**5%**	**6%**
	IGF-1*	2.82	2.73	2.71
	**% Change**	-	**-3%**	**-1%**

Notes: % = percentage frequencies; CRP = C-reactive protein; Fb = Fibrinogen; WBCC = White Blood Cell Counts; IGF-1 = Insulin-growth factor-1; * Log-transformed variable.

Table S7. **Fully adjusted longitudinal associations between immune and neuroendocrine profiles and all-cause hospitalisation, with *n* distribution.**

**Adjustments**	**Immune-Neuroendocrine Profiles**				
	**HR**	**SE**	**95% CI**		***p***
Psychiatric Disorders **(*n*=4,181)**					
Moderate Level - Model 3 [a]	1.12	0.09	0.95	1.32	0.182
High Level - Model 3 [a]	1.30	0.15	1.05	1.62	0.018
Sleep Disorders **(*n*=4,276)**					
Moderate Level - Model 3 [a]	2.29	0.77	1.19	4.41	0.013
High Level - Model 3 [a]	3.54	1.49	1.56	8.08	0.003
Blood Disorders **(*n*=4,139)**					
Moderate Level - Model 3 [a]	1.10	0.12	0.89	1.36	0.388
High Level - Model 3 [a]	1.68	0.23	1.28	2.19	<0.001
Circulatory Disorders **(*n*=3,163)**					
Moderate Level - Model 3 [a]	1.26	0.08	1.12	1.42	<0.001
High Level - Model 3 [a]	1.37	0.12	1.16	1.63	<0.001
Digestive Disorders **(*n*=3,188)**					
Moderate Level - Model 3 [a]	1.04	0.07	0.92	1.18	0.545
High Level - Model 3 [a]	0.93	0.09	0.76	1.13	0.460
Endocrine Disorders **(*n*=3,749)**					
Moderate Level - Model 3 [a]	1.26	0.08	1.11	1.43	<0.001
High Level - Model 3 [a]	1.38	0.13	1.16	1.66	<0.001
Genitourinary Disorders **(*n*=3,524)**					
Moderate Level - Model 3 [a]	1.18	0.09	1.02	1.36	0.027
High Level - Model 3 [a]	1.35	0.14	1.10	1.65	0.004
Infectious Disorders **(*n*=4,118)**					
Moderate Level - Model 3 [a]	1.02	0.10	0.85	1.23	0.810
High Level - Model 3 [a]	1.38	0.18	1.08	1.78	0.011
Musculoskeletal Disorders **(*n*=3,441)**					
Moderate Level - Model 3 [a]	1.13	0.08	0.99	1.30	0.063
High Level - Model 3 [a]	1.28	0.13	1.06	1.55	0.012
Nervous Disorders **(*n*=4,057)**					
Moderate Level - Model 3 [a]	1.12	0.11	0.93	1.35	0.220
High Level - Model 3 [a]	0.97	0.14	0.73	1.28	0.808
Respiratory Disorders **(*n*=3,846)**					
Moderate Level - Model 3 [a]	1.26	0.10	1.09	1.47	0.002
High Level - Model 3 [a]	1.99	0.19	1.64	2.40	<0.001
Skin Disorders **(*n*=4,045)**					
Moderate Level - Model 3 [a]	1.00	0.10	0.82	1.23	0.968
High Level - Model 3 [a]	1.19	0.17	0.90	1.58	0.231

Notes: The *low-risk* group is the reference; HR = Hazard ratio; SE = standard errors; CI = confidence interval; *p* = significance value.
a All variables: Baseline immune and neuroendocrine profiles; age; sex; 10 PCs; CRP PGS; WBCC PGS; IGF-1 PGS; PGS Anxiety; PGS Depression; PGS Schizophrenia; PGS Insomnia; PGS; Pain; education; wealth; smoking status; alcohol consumption; mobility

Table S8. **False discovery rate (FDR) adjusted longitudinal associations of immune-neuroendocrine profiles and individual biomarkers with all-cause hospitalisation (*n*=4,181)**

Disorder	Risk Level	Exposure	Alpha		FDR		Change
			p value	Significant	q value	Significant	
Psychiatric	Moderate	Profiles	0.182	FALSE	0.273	FALSE	FALSE
Psychiatric	High	Profiles	0.018	TRUE	0.033	TRUE	FALSE
Sleep	Moderate	Profiles	0.013	TRUE	0.026	TRUE	FALSE
Sleep	High	Profiles	0.003	TRUE	0.009	TRUE	FALSE
Blood	Moderate	Profiles	0.388	FALSE	0.490	FALSE	FALSE
Blood	High	Profiles	<0.001	TRUE	<0.001	TRUE	FALSE
Circulatory	Moderate	Profiles	<0.001	TRUE	<0.001	TRUE	FALSE
Circulatory	High	Profiles	<0.001	TRUE	<0.001	TRUE	FALSE
Digestive	Moderate	Profiles	0.545	FALSE	0.623	FALSE	FALSE
Digestive	High	Profiles	0.460	FALSE	0.552	FALSE	FALSE
Endocrine	Moderate	Profiles	<0.001	TRUE	<0.001	TRUE	FALSE
Endocrine	High	Profiles	<0.001	TRUE	<0.001	TRUE	FALSE
Genitourinary	Moderate	Profiles	0.027	TRUE	0.046	TRUE	FALSE
Genitourinary	High	Profiles	0.004	TRUE	0.011	TRUE	FALSE
Infectious	Moderate	Profiles	0.810	FALSE	0.845	FALSE	FALSE
Infectious	High	Profiles	0.011	TRUE	0.026	TRUE	FALSE
Musculoskeletal	Moderate	Profiles	0.063	FALSE	0.101	FALSE	FALSE
Musculoskeletal	High	Profiles	0.012	TRUE	0.026	TRUE	FALSE
Nervous	Moderate	Profiles	0.220	FALSE	0.308	FALSE	FALSE
Nervous	High	Profiles	0.808	FALSE	0.845	FALSE	FALSE
Respiratory	Moderate	Profiles	0.002	TRUE	0.007	TRUE	FALSE
Respiratory	High	Profiles	<0.001	TRUE	<0.001	TRUE	FALSE
Skin	Moderate	Profiles	0.968	FALSE	0.968	FALSE	FALSE
Skin	High	Profiles	0.231	FALSE	0.308	FALSE	FALSE
Blood	n/a	CRP	0.001	TRUE	0.004	TRUE	FALSE
Blood	n/a	Fb	0.007	TRUE	0.021	TRUE	FALSE
Blood	n/a	IGF1	0.349	FALSE	0.487	FALSE	FALSE
Blood	n/a	WBCC	0.052	FALSE	0.113	FALSE	FALSE
Circulatory	n/a	CRP	5e-04	TRUE	0.003	TRUE	FALSE
Circulatory	n/a	Fb	<0.001	TRUE	<0.001	TRUE	FALSE
Circulatory	n/a	IGF1	0.273	FALSE	0.400	FALSE	FALSE
Circulatory	n/a	WBCC	5e-04	TRUE	0.003	TRUE	FALSE
Digestive	n/a	CRP	0.620	FALSE	0.775	FALSE	FALSE
Digestive	n/a	Fb	0.580	FALSE	0.773	FALSE	FALSE
Digestive	n/a	IGF1	0.962	FALSE	0.962	FALSE	FALSE
Digestive	n/a	WBCC	0.962	FALSE	0.962	FALSE	FALSE
Endocrine	n/a	CRP	5e-04	TRUE	0.003	TRUE	FALSE

Endocrine	n/a	Fb	<0.001	TRUE	<0.001	TRUE	FALSE
Endocrine	n/a	IGF1	0.106	FALSE	0.197	FALSE	FALSE
Endocrine	n/a	WBCC	0.001	TRUE	0.004	TRUE	FALSE
Genitourinary	n/a	CRP	0.002	TRUE	0.007	TRUE	FALSE
Genitourinary	n/a	Fb	0.009	TRUE	0.024	TRUE	FALSE
Genitourinary	n/a	IGF1	0.732	FALSE	0.817	FALSE	FALSE
Genitourinary	n/a	WBCC	0.003	TRUE	0.010	TRUE	FALSE
Infectious	n/a	CRP	0.076	FALSE	0.159	FALSE	FALSE
Infectious	n/a	Fb	0.111	FALSE	0.197	FALSE	FALSE
Infectious	n/a	IGF1	0.761	FALSE	0.830	FALSE	FALSE
Infectious	n/a	WBCC	0.093	FALSE	0.186	FALSE	FALSE
Musculoskeletal	n/a	CRP	5e-04	TRUE	0.003	TRUE	FALSE
Musculoskeletal	n/a	Fb	0.273	FALSE	0.400	FALSE	FALSE
Musculoskeletal	n/a	IGF1	0.617	FALSE	0.775	FALSE	FALSE
Musculoskeletal	n/a	WBCC	0.197	FALSE	0.326	FALSE	FALSE
Nervous	n/a	CRP	0.630	FALSE	0.775	FALSE	FALSE
Nervous	n/a	Fb	0.866	FALSE	0.921	FALSE	FALSE
Nervous	n/a	IGF1	0.178	FALSE	0.305	FALSE	FALSE
Nervous	n/a	WBCC	0.703	FALSE	0.813	FALSE	FALSE
Psychiatric	n/a	CRP	0.018	TRUE	0.041	TRUE	FALSE
Psychiatric	n/a	Fb	0.109	FALSE	0.197	FALSE	FALSE
Psychiatric	n/a	IGF1	0.231	FALSE	0.370	FALSE	FALSE
Psychiatric	n/a	WBCC	0.008	TRUE	0.023	TRUE	FALSE
Respiratory	n/a	CRP	5e-04	TRUE	0.003	TRUE	FALSE
Respiratory	n/a	Fb	<0.001	TRUE	<0.001	TRUE	FALSE
Respiratory	n/a	IGF1	0.002	TRUE	0.007	TRUE	FALSE
Respiratory	n/a	WBCC	5e-04	TRUE	0.003	TRUE	FALSE
Skin	n/a	CRP	0.355	FALSE	0.487	FALSE	FALSE
Skin	n/a	Fb	0.883	FALSE	0.921	FALSE	FALSE
Skin	n/a	IGF1	0.711	FALSE	0.813	FALSE	FALSE
Skin	n/a	WBCC	0.275	FALSE	0.400	FALSE	FALSE
Sleep	n/a	CRP	0.001	TRUE	0.004	TRUE	FALSE
Sleep	n/a	Fb	0.013	TRUE	0.031	TRUE	FALSE
Sleep	n/a	IGF1	0.700	FALSE	0.813	FALSE	FALSE
Sleep	n/a	WBCC	0.013	TRUE	0.031	TRUE	FALSE

Table S9a. **Longitudinal associations between immune and neuroendocrine profiles and hospitalisation for mental and behavioural disorders (*n*=4,181)**

Adjustments	Immune and Neuroendocrine Profiles				
	HR	SE	95% CI		*p*
Moderate-risk **Profile | Mental and Behavioural Disorders**					
Model 1: *Unadjusted*	1.52	0.12	1.30	1.78	<0.001
Model 2: *Model 1 + demographics & genetics* [a]	1.33	0.11	1.13	1.55	0.001
Model 3: *Model 2 + Fully Adjusted* [b]	1.12	0.09	0.95	1.32	0.182
Model 2a: *Demographics*	1.37	0.11	1.17	1.60	<0.001
Model 2b: *Genetics*	1.33	0.11	1.13	1.55	0.001
Model 3a: Model 3 + *Medication*	1.12	0.09	0.95	1.31	0.187
Model 3b: Model 3 + *Physical Activity*	1.10	0.09	0.93	1.29	0.269
Model 3c: Model 3 + *Body Mass Index*	1.09	0.09	0.92	1.28	0.339
Model 3d: Model 3 + *Fully Adjusted* [c]	1.07	0.09	0.90	1.26	0.458
Model 4a: Model 2 + *Health*	1.30	0.11	1.11	1.53	0.001
Model 4b: Model 3 + *Health*	1.11	0.09	0.94	1.30	0.223
Model 4c: Model 3 + *Fully Adjusted* [d]	1.06	0.09	0.89	1.25	0.514
High-risk **Profile | Mental and Behavioural Disorders**					
Model 1: *Unadjusted*	1.95	0.21	1.57	2.41	<0.001
Model 2: *Model 1 + demographics & genetics* [a]	1.71	0.19	1.38	2.12	<0.001
Model 3: *Model 2 + Fully Adjusted* [b]	1.30	0.15	1.05	1.62	0.018
Model 2a: *Demographics*	1.75	0.19	1.41	2.17	<0.001
Model 2b: *Genetics*	1.71	0.19	1.38	2.12	<0.001
Model 3a: Model 3 + *Medication*	1.31	0.15	1.05	1.63	0.016
Model 3b: Model 3 + *Physical Activity*	1.24	0.14	1.00	1.55	0.052
Model 3c: Model 3 + *Body Mass Index*	1.26	0.14	1.00	1.57	0.046
Model 3d: Model 3 + *Fully Adjusted* [c]	1.21	0.14	0.96	1.51	0.101
Model 4a: Model 2 + *Health*	1.69	0.19	1.36	2.09	<0.001
Model 4b: Model 3 + *Health*	1.28	0.14	1.03	1.60	0.026
Model 4c: Model 3 + *Fully Adjusted* [d]	1.19	0.14	0.95	1.50	0.125

Notes: The *low-risk* group is the reference; HR = hazard ratio; SE = standard error; CI = confidence interval; *p* = significance value.

a *Demographic and genetic variables*: age; sex; 10 principal components (PCs); C-reactive Protein (CRP) polygenic score (PGS); White Blood Cell Counts (WBCC) PGS; Insulin Growth Factor-1 (IGF-1) PGS; Anxiety PGS; Depression PGS; Schizophrenia PGS; Insomnia PGS; Pain PGS.

b All variables: age; sex; 10 PCs; CRP PGS; WBCC PGS; IGF-1 PGS; PGS; Anxiety PGS; Depression PGS; Schizophrenia PGS; Insomnia PGS; Pain PGS; education; wealth; smoking status; alcohol consumption; mobility.

c Additional variables: medication, physical activity; BMI.

d Additional variables: health (i.e., chronic lung disease; coronary heart disease; abnormal heart rhythm; heart murmur; congestive heart failure; angina; hypertension; diabetes; cancer; Parkinson's; Alzheimer's; dementia; asthma; arthritis; osteoporosis; psychiatric disorder).

Table S9b. **Longitudinal associations between C-reactive protein and hospitalisation for mental and behavioural disorders (*n*=4,181)**

Adjustments	CRP				
	HR	SE	95% CI		*p*
**Mental and Behavioural Disorders**					
Model 1: *Unadjusted*	1.40	0.07	1.27	1.56	<0.001
Model 2: *Model 1 + demographics & genetics* [a]	1.30	0.07	1.17	1.448	<0.001
Model 3: *Model 2 + Fully Adjusted* [b]	1.14	0.06	1.02	1.27	0.018
Model 2a: *Demographics*	1.30	0.07	1.17	1.45	<0.001
Model 2b: *Genetics*	1.39	0.07	1.25	1.54	<0.001
Model 3a: Model 3 + *Medication*	1.14	0.06	1.02	1.27	0.017
Model 3b: Model 3 + *Physical Activity*	1.11	0.06	1.00	1.24	0.053
Model 3c: Model 3 + *Body Mass Index*	1.12	0.07	1.00	1.25	0.059
Model 3d: Model 3 + *Fully Adjusted* [c]	1.09	0.06	0.97	1.22	0.132
Model 4a: Model 2 + *Health*	1.29	0.07	1.16	1.430	<0.001
Model 4b: Model 3 + *Health*	1.13	0.06	1.01	1.259	0.029
Model 4c: Model 3 + *Fully Adjusted* [d]	1.08	0.06	0.97	1.213	0.175

Notes: The *low-risk* group is the reference; HR = hazard ratio; SE = standard error; CI = confidence interval; *p* = significance value.

a *Demographic and genetic variables*: age; sex; 10 principal components (PCs); C-reactive Protein (CRP) polygenic score (PGS); White Blood Cell Counts (WBCC) PGS; Insulin Growth Factor-1 (IGF-1) PGS; Anxiety PGS; Depression PGS; Schizophrenia PGS; Insomnia PGS; Pain PGS.

b All variables: age; sex; 10 PCs; CRP PGS; WBCC PGS; IGF-1 PGS; PGS; Anxiety PGS; Depression PGS; Schizophrenia PGS; Insomnia PGS; Pain PGS; education; wealth; smoking status; alcohol consumption; mobility.

c Additional variables: medication, physical activity; BMI.

d Additional variables: health (i.e., chronic lung disease; coronary heart disease; abnormal heart rhythm; heart murmur; congestive heart failure; angina; hypertension; diabetes; cancer; Parkinson's; Alzheimer's; dementia; asthma; arthritis; osteoporosis; psychiatric disorder).

Table S9c. **Longitudinal associations between fibrinogen and hospitalisation for mental and behavioural disorders (*n*=4,181)**

**Adjustments**	**Fb**				
	**HR**	**SE**	**95% CI**		***p***
**Mental and Behavioural Disorders**					
Model 1: *Unadjusted*	1.53	0.10	1.35	1.73	<0.001
Model 2: *Model 1 + demographics & genetics* [a]	1.37	0.09	1.21	1.56	<0.001
Model 3: *Model 2 + Fully Adjusted* [b]	1.11	0.08	0.98	1.27	0.109
Model 2a: *Demographics*	1.40	0.09	1.23	1.59	<0.001
Model 2b: *Genetics*	1.49	0.09	1.32	1.69	<0.001
Model 3a: Model 3 + *Medication*	1.11	0.08	0.98	1.27	0.109
Model 3b: Model 3 + *Physical Activity*	1.09	0.07	0.96	1.25	0.187
Model 3c: Model 3 + *Body Mass Index*	1.10	0.08	0.96	1.25	0.177
Model 3d: Model 3 + *Fully Adjusted* [c]	1.08	0.07	0.94	1.23	0.275
Model 4a: Model 2 + *Health*	1.37	0.09	1.20	1.56	<0.001
Model 4b: Model 3 + *Health*	1.12	0.08	0.98	1.27	0.106
Model 4c: Model 3 + *Fully Adjusted* [d]	1.08	0.07	0.94	1.23	0.262

Notes: The *low-risk* group is the reference; HR = hazard ratio; SE = standard error; CI = confidence interval; *p* = significance value.

a *Demographic and genetic variables*: age; sex; 10 principal components (PCs); C-reactive Protein (CRP) polygenic score (PGS); White Blood Cell Counts (WBCC) PGS; Insulin Growth Factor-1 (IGF-1) PGS; Anxiety PGS; Depression PGS; Schizophrenia PGS; Insomnia PGS; Pain PGS.

b All variables: age; sex; 10 PCs; CRP PGS; WBCC PGS; IGF-1 PGS; PGS; Anxiety PGS; Depression PGS; Schizophrenia PGS; Insomnia PGS; Pain PGS; education; wealth; smoking status; alcohol consumption; mobility.

c Additional variables: medication, physical activity; BMI.

d Additional variables: health (i.e., chronic lung disease; coronary heart disease; abnormal heart rhythm; heart murmur; congestive heart failure; angina; hypertension; diabetes; cancer; Parkinson's; Alzheimer's; dementia; asthma; arthritis; osteoporosis; psychiatric disorder).

Table S9d. **Longitudinal associations between white blood cell counts and hospitalisation for mental and behavioural disorders (*n*=4,181)**

Adjustments	WBCC				
	**HR**	**SE**	**95% CI**		***p***
**Mental and Behavioural Disorders**					
Model 1: *Unadjusted*	3.69	0.55	2.76	4.94	<0.001
Model 2: *Model 1 + demographics & genetics* [a]	3.66	0.56	2.72	4.92	<0.001
Model 3: *Model 2 + Fully Adjusted* [b]	1.53	0.25	1.12	2.09	0.008
Model 2a: *Demographics*	3.45	0.52	2.57	4.63	<0.001
Model 2b: *Genetics*	3.82	0.57	2.85	5.12	<0.001
Model 3a: Model 3 + *Medication*	1.54	0.25	1.12	2.10	0.007
Model 3b: Model 3 + *Physical Activity*	1.48	0.24	1.08	2.03	0.014
Model 3c: Model 3 + *Body Mass Index*	1.48	0.24	1.08	2.04	0.015
Model 3d: Model 3 + *Fully Adjusted* [c]	1.45	0.23	1.06	1.99	0.022
Model 4a: Model 2 + *Health*	3.57	0.54	2.65	4.80	<0.001
Model 4b: Model 3 + *Health*	1.51	0.24	1.10	2.07	0.010
Model 4c: Model 3 + *Fully Adjusted* [d]	1.43	0.23	1.05	1.97	0.025

Notes: The *low-risk* group is the reference; HR = hazard ratio; SE = standard error; CI = confidence interval; *p* = significance value.

a *Demographic and genetic variables*: age; sex; 10 principal components (PCs); C-reactive Protein (CRP) polygenic score (PGS); White Blood Cell Counts (WBCC) PGS; Insulin Growth Factor-1 (IGF-1) PGS; Anxiety PGS; Depression PGS; Schizophrenia PGS; Insomnia PGS; Pain PGS.

b All variables: age; sex; 10 PCs; CRP PGS; WBCC PGS; IGF-1 PGS; PGS; Anxiety PGS; Depression PGS; Schizophrenia PGS; Insomnia PGS; Pain PGS; education; wealth; smoking status; alcohol consumption; mobility.

c Additional variables: medication, physical activity; BMI.

d Additional variables: health (i.e., chronic lung disease; coronary heart disease; abnormal heart rhythm; heart murmur; congestive heart failure; angina; hypertension; diabetes; cancer; Parkinson's; Alzheimer's; dementia; asthma; arthritis; osteoporosis; psychiatric disorder).

Table S9e. **Longitudinal associations between insulin growth factor-1 and hospitalisation for mental and behavioural disorders (*n*=4,181)**

**Adjustments**	**IGF-1**				
	**HR**	**SE**	**95% CI**		***p***
**Mental and Behavioural Disorders**					
Model 1: *Unadjusted*	0.65	0.07	0.52	0.81	<0.001
Model 2: *Model 1 + demographics & genetics* [a]	0.89	0.10	0.71	1.11	0.293
Model 3: *Model 2 + Fully Adjusted* [b]	0.88	0.10	0.70	1.09	0.231
Model 2a: *Demographics*	0.88	0.10	0.70	1.10	0.246
Model 2b: *Genetics*	0.66	0.07	0.53	0.82	<0.001
Model 3a: Model 3 + *Medication*	0.87	0.10	0.70	1.09	0.220
Model 3b: Model 3 + *Physical Activity*	0.89	0.10	0.71	1.10	0.278
Model 3c: Model 3 + *Body Mass Index*	0.88	0.10	0.70	1.09	0.235
Model 3d: Model 3 + *Fully Adjusted* [c]	0.89	0.10	0.71	1.10	0.276
Model 4a: Model 2 + *Health*	0.89	0.10	0.71	1.11	0.305
Model 4b: Model 3 + *Health*	0.87	0.10	0.70	1.09	0.226
Model 4c: Model 3 + *Fully Adjusted* [d]	0.89	0.10	0.71	1.10	0.270

Notes: The *low-risk* group is the reference; HR = hazard ratio; SE = standard error; CI = confidence interval; *p* = significance value.

a *Demographic and genetic variables*: age; sex; 10 principal components (PCs); C-reactive Protein (CRP) polygenic score (PGS); White Blood Cell Counts (WBCC) PGS; Insulin Growth Factor-1 (IGF-1) PGS; Anxiety PGS; Depression PGS; Schizophrenia PGS; Insomnia PGS; Pain PGS.

b All variables: age; sex; 10 PCs; CRP PGS; WBCC PGS; IGF-1 PGS; PGS; Anxiety PGS; Depression PGS; Schizophrenia PGS; Insomnia PGS; Pain PGS; education; wealth; smoking status; alcohol consumption; mobility.

c Additional variables: medication, physical activity; BMI.

d Additional variables: health (i.e., chronic lung disease; coronary heart disease; abnormal heart rhythm; heart murmur; congestive heart failure; angina; hypertension; diabetes; cancer; Parkinson's; Alzheimer's; dementia; asthma; arthritis; osteoporosis; psychiatric disorder).

Table S9f. **Longitudinal associations between immune and neuroendocrine profiles and hospitalisation for sleep disorders (*n*=4,181)**

Adjustments	Immune and Neuroendocrine Profiles				
	HR	SE	95% CI		*p*
***Moderate-risk* Profile | Sleep Disorders**					
Model 1: *Unadjusted*	2.18	0.71	1.15	4.12	0.017
Model 2: *Model 1 + demographics & genetics* [a]	2.33	0.77	1.22	4.46	0.010
Model 3: *Model 2 + Fully Adjusted* [b]	2.29	0.77	1.19	4.41	0.013
Model 2a: *Demographics*	2.47	0.81	1.30	4.69	0.006
Model 2b: *Genetics*	2.08	0.68	1.09	3.94	0.025
Model 3a: Model 3 + *Medication*	2.29	0.77	1.19	4.41	0.013
Model 3b: Model 3 + *Physical Activity*	2.13	0.71	1.10	4.11	0.025
Model 3c: Model 3 + *Body Mass Index*	1.91	0.66	0.98	3.75	0.058
Model 3d: Model 3 + *Fully Adjusted* [c]	1.78	0.62	0.91	3.50	0.093
Model 4a: Model 2 + *Health*	2.33	0.77	1.22	4.46	0.010
Model 4b: Model 3 + *Health*	2.28	0.76	1.18	4.40	0.014
Model 4c: Model 3 + *Fully Adjusted* [d]	1.78	0.61	0.91	3.50	0.093
***High-risk* Profile | Sleep Disorders**					
Model 1: *Unadjusted*	3.10	1.25	1.41	6.84	0.005
Model 2: *Model 1 + demographics & genetics* [a]	3.51	1.45	1.57	7.87	0.002
Model 3: *Model 2 + Fully Adjusted* [b]	3.54	1.49	1.56	8.08	0.003
Model 2a: *Demographics*	3.65	1.49	1.65	8.11	0.001
Model 2b: *Genetics*	3.02	1.23	1.36	6.70	0.007
Model 3a: Model 3 + *Medication*	3.54	1.49	1.56	8.08	0.003
Model 3b: Model 3 + *Physical Activity*	3.06	1.30	1.33	7.04	0.009
Model 3c: Model 3 + *Body Mass Index*	2.82	1.22	1.21	6.59	0.016
Model 3d: Model 3 + *Fully Adjusted* [c]	2.44	1.07	1.04	5.75	0.041
Model 4a: Model 2 + *Health*	3.51	1.45	1.57	7.87	0.00
Model 4b: Model 3 + *Health*	3.53	1.49	1.55	8.06	0.00
Model 4c: Model 3 + *Fully Adjusted* [d]	2.44	1.07	1.04	5.75	0.04

Notes: The *low-risk* group is the reference; HR = hazard ratio; SE = standard error; CI = confidence interval; *p* = significance value.

a *Demographic and genetic variables*: age; sex; 10 principal components (PCs); C-reactive Protein (CRP) polygenic score (PGS); White Blood Cell Counts (WBCC) PGS; Insulin Growth Factor-1 (IGF-1) PGS; Anxiety PGS; Depression PGS; Schizophrenia PGS; Insomnia PGS; Pain PGS.

b All variables: age; sex; 10 PCs; CRP PGS; WBCC PGS; IGF-1 PGS; PGS; Anxiety PGS; Depression PGS; Schizophrenia PGS; Insomnia PGS; Pain PGS; education; wealth; smoking status; alcohol consumption; mobility.

c Additional variables: medication, physical activity; BMI.

d Additional variables: health (i.e., chronic lung disease; coronary heart disease; abnormal heart rhythm; heart murmur; congestive heart failure; angina; hypertension; diabetes; cancer; Parkinson's; Alzheimer's; dementia; asthma; arthritis; osteoporosis; psychiatric disorder).

Table S9g. **Longitudinal associations between C-reactive protein and hospitalisation for sleep disorders (*n*=4,276)**

**Adjustments**	**CRP**				
	**HR**	**SE**	**95% CI**		***p***
**Sleep Disorders**					
Model 1: *Unadjusted*	1.92	0.37	1.31	2.81	0.001
Model 2: *Model 1 + demographics & genetics* [a]	2.06	0.42	1.39	3.06	<0.001
Model 3: *Model 2 + Fully Adjusted* [b]	2.05	0.42	1.37	3.07	0.001
Model 2a: *Demographics*	2.11	0.42	1.43	3.11	<0.001
Model 2b: *Genetics*	1.88	0.38	1.27	2.78	0.002
Model 3a: Model 3 + *Medication*	2.05	0.42	1.37	3.07	0.001
Model 3b: Model 3 + *Physical Activity*	1.90	0.40	1.26	2.86	0.002
Model 3c: Model 3 + *Body Mass Index*	1.81	0.39	1.18	2.77	0.006
Model 3d: Model 3 + *Fully Adjusted* [c]	1.68	0.37	1.09	2.58	0.019
Model 4a: Model 2 + *Health*	2.06	0.42	1.39	3.06	<0.001
Model 4b: Model 3 + *Health*	2.04	0.42	1.36	3.07	0.001
Model 4c: Model 3 + *Fully Adjusted* [d]	1.68	0.37	1.09	2.58	0.019

Notes: The *low-risk* group is the reference; HR = hazard ratio; SE = standard error; CI = confidence interval; *p* = significance value.

a *Demographic and genetic variables*: age; sex; 10 principal components (PCs); C-reactive Protein (CRP) polygenic score (PGS); White Blood Cell Counts (WBCC) PGS; Insulin Growth Factor-1 (IGF-1) PGS; Anxiety PGS; Depression PGS; Schizophrenia PGS; Insomnia PGS; Pain PGS.

b All variables: age; sex; 10 PCs; CRP PGS; WBCC PGS; IGF-1 PGS; PGS; Anxiety PGS; Depression PGS; Schizophrenia PGS; Insomnia PGS; Pain PGS; education; wealth; smoking status; alcohol consumption; mobility.

c Additional variables: medication, physical activity; BMI.

d Additional variables: health (i.e., chronic lung disease; coronary heart disease; abnormal heart rhythm; heart murmur; congestive heart failure; angina; hypertension; diabetes; cancer; Parkinson's; Alzheimer's; dementia; asthma; arthritis; osteoporosis; psychiatric disorder).

Table S9h. **Longitudinal associations between fibrinogen and hospitalisation for sleep disorders (*n*=4,276)**

Adjustments	Fb				
	**HR**	**SE**	**95% CI**		***p***
**Sleep Disorders**					
Model 1: *Unadjusted*	1.64	0.39	1.03	2.62	0.037
Model 2: *Model 1 + demographics & genetics* [a]	1.80	0.41	1.15	2.82	0.010
Model 3: *Model 2 + Fully Adjusted* [b]	1.80	0.43	1.13	2.86	0.013
Model 2a: *Demographics*	1.81	0.42	1.16	2.84	0.009
Model 2b: *Genetics*	1.60	0.38	1.00	2.55	0.048
Model 3a: Model 3 + *Medication*	1.80	0.43	1.13	2.86	0.014
Model 3b: Model 3 + *Physical Activity*	1.68	0.41	1.04	2.70	0.032
Model 3c: Model 3 + *Body Mass Index*	1.67	0.41	1.02	2.71	0.040
Model 3d: Model 3 + *Fully Adjusted* [c]	1.58	0.40	0.96	2.58	0.070
Model 4a: Model 2 + *Health*	1.77	0.41	1.13	2.78	0.013
Model 4b: Model 3 + *Health*	1.79	0.43	1.12	2.85	0.014
Model 4c: Model 3 + *Fully Adjusted* [d]	1.57	0.40	0.96	2.58	0.071

Notes: The *low-risk* group is the reference; HR = hazard ratio; SE = standard error; CI = confidence interval; *p* = significance value.

a *Demographic and genetic variables*: age; sex; 10 principal components (PCs); C-reactive Protein (CRP) polygenic score (PGS); White Blood Cell Counts (WBCC) PGS; Insulin Growth Factor-1 (IGF-1) PGS; Anxiety PGS; Depression PGS; Schizophrenia PGS; Insomnia PGS; Pain PGS.

b All variables: age; sex; 10 PCs; CRP PGS; WBCC PGS; IGF-1 PGS; PGS; Anxiety PGS; Depression PGS; Schizophrenia PGS; Insomnia PGS; Pain PGS; education; wealth; smoking status; alcohol consumption; mobility.

c Additional variables: medication, physical activity; BMI.

d Additional variables: health (i.e., chronic lung disease; coronary heart disease; abnormal heart rhythm; heart murmur; congestive heart failure; angina; hypertension; diabetes; cancer; Parkinson's; Alzheimer's; dementia; asthma; arthritis; osteoporosis; psychiatric disorder).

Table S9i. **Longitudinal associations between white blood cell counts and hospitalisation for sleep disorders (*n*=4,276)**

**Adjustments**	**WBCC**				
	**HR**	**SE**	**95% CI**		***p***
**Sleep Disorders**					
Model 1: *Unadjusted*	3.77	2.12	1.25	11.36	0.018
Model 2: *Model 1 + demographics & genetics* [a]	1.80	0.41	1.15	2.82	0.010
Model 3: *Model 2 + Fully Adjusted* [b]	1.80	0.43	1.13	2.86	0.013
Model 2a: *Demographics*	1.81	0.42	1.16	2.84	0.009
Model 2b: *Genetics*	1.60	0.38	1.00	2.55	0.048
Model 3a: Model 3 + *Medication*	1.80	0.43	1.13	2.86	0.014
Model 3b: Model 3 + *Physical Activity*	1.68	0.41	1.04	2.70	0.032
Model 3c: Model 3 + *Body Mass Index*	1.67	0.41	1.02	2.71	0.040
Model 3d: Model 3 + *Fully Adjusted* [c]	1.58	0.40	0.96	2.58	0.070
Model 4a: Model 2 + *Health*	1.77	0.41	1.13	2.78	0.013
Model 4b: Model 3 + *Health*	1.79	0.43	1.12	2.85	0.014
Model 4c: Model 3 + *Fully Adjusted* [d]	1.57	0.40	0.96	2.58	0.071

Notes: The *low-risk* group is the reference; HR = hazard ratio; SE = standard error; CI = confidence interval; *p* = significance value.

a *Demographic and genetic variables*: age; sex; 10 principal components (PCs); C-reactive Protein (CRP) polygenic score (PGS); White Blood Cell Counts (WBCC) PGS; Insulin Growth Factor-1 (IGF-1) PGS; Anxiety PGS; Depression PGS; Schizophrenia PGS; Insomnia PGS; Pain PGS.

b All variables: age; sex; 10 PCs; CRP PGS; WBCC PGS; IGF-1 PGS; PGS; Anxiety PGS; Depression PGS; Schizophrenia PGS; Insomnia PGS; Pain PGS; education; wealth; smoking status; alcohol consumption; mobility.

c Additional variables: medication, physical activity; BMI.

d Additional variables: health (i.e., chronic lung disease; coronary heart disease; abnormal heart rhythm; heart murmur; congestive heart failure; angina; hypertension; diabetes; cancer; Parkinson's; Alzheimer's; dementia; asthma; arthritis; osteoporosis; psychiatric disorder).

Table S9j. **Longitudinal associations between insulin growth factor-1 and hospitalisation for sleep disorders (*n*=4,276)**

**Adjustments**	**IGF-1**				
	**HR**	**SE**	**95% CI**		***p***
**Sleep Disorders**					
Model 1: *Unadjusted*	1.09	0.48	0.46	2.57	0.854
Model 2: *Model 1 + demographics & genetics* [a]	0.79	0.36	0.33	1.91	0.606
Model 3: *Model 2 + Fully Adjusted* [b]	0.84	0.38	0.35	2.02	0.700
Model 2a: *Demographics*	0.75	0.34	0.31	1.80	0.513
Model 2b: *Genetics*	1.13	0.50	0.48	2.67	0.786
Model 3a: Model 3 + *Medication*	0.84	0.38	0.35	2.02	0.700
Model 3b: Model 3 + *Physical Activity*	0.87	0.38	0.37	2.07	0.751
Model 3c: Model 3 + *Body Mass Index*	0.88	0.39	0.37	2.08	0.761
Model 3d: Model 3 + *Fully Adjusted* [c]	0.92	0.40	0.39	2.16	0.845
Model 4a: Model 2 + *Health*	0.81	0.36	0.34	1.95	0.640
Model 4b: Model 3 + *Health*	0.85	0.38	0.35	2.03	0.712
Model 4c: Model 3 + *Fully Adjusted* [d]	0.92	0.40	0.39	2.17	0.848

Notes: The *low-risk* group is the reference; HR = hazard ratio; SE = standard error; CI = confidence interval; *p* = significance value.

a *Demographic and genetic variables*: age; sex; 10 principal components (PCs); C-reactive Protein (CRP) polygenic score (PGS); White Blood Cell Counts (WBCC) PGS; Insulin Growth Factor-1 (IGF-1) PGS; Anxiety PGS; Depression PGS; Schizophrenia PGS; Insomnia PGS; Pain PGS.

b All variables: age; sex; 10 PCs; CRP PGS; WBCC PGS; IGF-1 PGS; PGS; Anxiety PGS; Depression PGS; Schizophrenia PGS; Insomnia PGS; Pain PGS; education; wealth; smoking status; alcohol consumption; mobility.

c Additional variables: medication, physical activity; BMI.

d Additional variables: health (i.e., chronic lung disease; coronary heart disease; abnormal heart rhythm; heart murmur; congestive heart failure; angina; hypertension; diabetes; cancer; Parkinson's; Alzheimer's; dementia; asthma; arthritis; osteoporosis; psychiatric disorder).

Table S9k. **Longitudinal associations between immune and neuroendocrine profiles and hospitalisation for diseases of the blood and blood-forming organs and certain disorders involving the immune mechanism (*n*=4,139)**

Adjustments	Immune and Neuroendocrine Profiles				
	**HR**	**SE**	**95% CI**		***p***
***Moderate-risk* Profile | Blood Disorders**					
Model 1: *Unadjusted*	1.38	0.15	1.13	1.70	0.002
Model 2: *Model 1 + demographics & genetics* [a]	1.19	0.13	0.97	1.47	0.099
Model 3: *Model 2 + Fully Adjusted* [b]	1.10	0.12	0.89	1.36	0.388
Model 2a: *Demographics*	1.21	0.13	0.99	1.49	0.070
Model 2b: *Genetics*	1.37	0.14	1.12	1.69	0.003
Model 3a: Model 3 + *Medication*	1.10	0.12	0.89	1.35	0.398
Model 3b: Model 3 + *Physical Activity*	1.07	0.12	0.87	1.32	0.532
Model 3c: Model 3 + *Body Mass Index*	1.09	0.12	0.87	1.35	0.458
Model 3d: Model 3 + *Fully Adjusted* [c]	1.06	0.12	0.86	1.32	0.585
Model 4a: Model 2 + *Health*	1.19	0.13	0.97	1.47	0.099
Model 4b: Model 3 + *Health*	1.09	0.12	0.88	1.34	0.432
Model 4c: Model 3 + *Fully Adjusted* [d]	1.06	0.12	0.85	1.31	0.623
***High-risk* Profile | Blood Disorders**					
Model 1: *Unadjusted*	2.21	0.29	1.71	2.85	<0.001
Model 2: *Model 1 + demographics & genetics* [a]	1.92	0.26	1.48	2.49	<0.001
Model 3: *Model 2 + Fully Adjusted* [b]	1.68	0.23	1.28	2.19	<0.001
Model 2a: *Demographics*	1.96	0.26	1.51	2.54	<0.001
Model 2b: *Genetics*	2.16	0.29	1.67	2.80	<0.001
Model 3a: Model 3 + *Medication*	1.68	0.23	1.29	2.19	<0.001
Model 3b: Model 3 + *Physical Activity*	1.59	0.22	1.21	2.07	0.001
Model 3c: Model 3 + *Body Mass Index*	1.65	0.23	1.26	2.17	<0.001
Model 3d: Model 3 + *Fully Adjusted* [c]	1.58	0.22	1.20	2.08	0.001
Model 4a: Model 2 + *Health*	1.92	0.26	1.48	2.49	<0.001
Model 4b: Model 3 + *Health*	1.67	0.23	1.28	2.18	<0.001
Model 4c: Model 3 + *Fully Adjusted* [d]	1.58	0.22	1.20	2.08	0.001

Notes: The *low-risk* group is the reference; HR = hazard ratio; SE = standard error; CI = confidence interval; *p* = significance value.

a *Demographic and genetic variables*: age; sex; 10 principal components (PCs); C-reactive Protein (CRP) polygenic score (PGS); White Blood Cell Counts (WBCC) PGS; Insulin Growth Factor-1 (IGF-1) PGS; Anxiety PGS; Depression PGS; Schizophrenia PGS; Insomnia PGS; Pain PGS.

b All variables: age; sex; 10 PCs; CRP PGS; WBCC PGS; IGF-1 PGS; PGS; Anxiety PGS; Depression PGS; Schizophrenia PGS; Insomnia PGS; Pain PGS; education; wealth; smoking status; alcohol consumption; mobility.

c Additional variables: medication, physical activity; BMI.

d Additional variables: health (i.e., chronic lung disease; coronary heart disease; abnormal heart rhythm; heart murmur; congestive heart failure; angina; hypertension; diabetes; cancer; Parkinson's; Alzheimer's; dementia; asthma; arthritis; osteoporosis; psychiatric disorder).

Table S91. **Longitudinal associations between C-reactive protein and hospitalisation for diseases of the blood and blood-forming organs and certain disorders involving the immune mechanism (*n*=4,139)**

Adjustments	CRP				
	HR	SE	95% CI		*p*
**Blood Disorders**					
Model 1: *Unadjusted*	1.49	0.10	1.31	1.69	<0.001
Model 2: *Model 1 + demographics & genetics* [a]	1.37	0.09	1.19	1.56	<0.001
Model 3: *Model 2 + Fully Adjusted* [b]	1.27	0.09	1.11	1.46	0.001
Model 2a: *Demographics*	1.39	0.10	1.21	1.58	<0.001
Model 2b: *Genetics*	1.47	0.10	1.29	1.67	<0.001
Model 3a: Model 3 + *Medication*	1.28	0.09	1.11	1.46	0.001
Model 3b: Model 3 + *Physical Activity*	1.24	0.09	1.08	1.42	0.003
Model 3c: Model 3 + *Body Mass Index*	1.27	0.09	1.10	1.47	0.001
Model 3d: Model 3 + *Fully Adjusted* [c]	1.24	0.09	1.07	1.43	0.004
Model 4a: Model 2 + *Health*	1.37	0.09	1.19	1.56	<0.001
Model 4b: Model 3 + *Health*	1.27	0.09	1.11	1.46	0.001
Model 4c: Model 3 + *Fully Adjusted* [d]	1.24	0.09	1.07	1.43	0.004

Notes: The *low-risk* group is the reference; HR = hazard ratio; SE = standard error; CI = confidence interval; *p* = significance value.

a *Demographic and genetic variables*: age; sex; 10 principal components (PCs); C-reactive Protein (CRP) polygenic score (PGS); White Blood Cell Counts (WBCC) PGS; Insulin Growth Factor-1 (IGF-1) PGS; Anxiety PGS; Depression PGS; Schizophrenia PGS; Insomnia PGS; Pain PGS.

b All variables: age; sex; 10 PCs; CRP PGS; WBCC PGS; IGF-1 PGS; PGS; Anxiety PGS; Depression PGS; Schizophrenia PGS; Insomnia PGS; Pain PGS; education; wealth; smoking status; alcohol consumption; mobility.

c Additional variables: medication, physical activity; BMI.

d Additional variables: health (i.e., chronic lung disease; coronary heart disease; abnormal heart rhythm; heart murmur; congestive heart failure; angina; hypertension; diabetes; cancer; Parkinson's; Alzheimer's; dementia; asthma; arthritis; osteoporosis; psychiatric disorder).

Table S9m. **Longitudinal associations between fibrinogen and hospitalisation for diseases of the blood and blood-forming organs and certain disorders involving the immune mechanism (*n*=4,139)**

**Adjustments**	**Fb**				
	**HR**	**SE**	**95% CI**		***p***
**Blood Disorders**					
Model 1: *Unadjusted*	1.49	0.12	1.28	1.75	<0.001
Model 2: *Model 1 + demographics & genetics* [a]	1.38	0.12	1.17	1.63	<0.001
Model 3: *Model 2 + Fully Adjusted* [b]	1.26	0.11	1.07	1.49	0.007
Model 2a: *Demographics*	1.38	0.12	1.18	1.63	<0.001
Model 2b: *Genetics*	1.48	0.12	1.26	1.73	<0.001
Model 3a: Model 3 + *Medication*	1.26	0.11	1.07	1.49	0.007
Model 3b: Model 3 + *Physical Activity*	1.24	0.11	1.05	1.46	0.013
Model 3c: Model 3 + *Body Mass Index*	1.25	0.11	1.06	1.48	0.010
Model 3d: Model 3 + *Fully Adjusted* [c]	1.23	0.11	1.04	1.46	0.016
Model 4a: Model 2 + *Health*	1.37	0.12	1.17	1.62	<0.001
Model 4b: Model 3 + *Health*	1.26	0.11	1.07	1.49	0.007
Model 4c: Model 3 + *Fully Adjusted* [d]	1.23	0.11	1.04	1.46	0.015

Notes: The *low-risk* group is the reference; HR = hazard ratio; SE = standard error; CI = confidence interval; *p* = significance value.

a *Demographic and genetic variables*: age; sex; 10 principal components (PCs); C-reactive Protein (CRP) polygenic score (PGS); White Blood Cell Counts (WBCC) PGS; Insulin Growth Factor-1 (IGF-1) PGS; Anxiety PGS; Depression PGS; Schizophrenia PGS; Insomnia PGS; Pain PGS.

b All variables: age; sex; 10 PCs; CRP PGS; WBCC PGS; IGF-1 PGS; PGS; Anxiety PGS; Depression PGS; Schizophrenia PGS; Insomnia PGS; Pain PGS; education; wealth; smoking status; alcohol consumption; mobility.

c Additional variables: medication, physical activity; BMI.

d Additional variables: health (i.e., chronic lung disease; coronary heart disease; abnormal heart rhythm; heart murmur; congestive heart failure; angina; hypertension; diabetes; cancer; Parkinson's; Alzheimer's; dementia; asthma; arthritis; osteoporosis; psychiatric disorder).

Table S9n. **Longitudinal associations between white blood cell counts and hospitalisation for diseases of the blood and blood-forming organs and certain disorders involving the immune mechanism (*n*=4,139)**

**Adjustments**	**WBCC**				
	**HR**	**SE**	**95% CI**		***p***
**Blood Disorders**					
Model 1: *Unadjusted*	2.04	0.39	1.40	2.97	<0.001
Model 2: *Model 1 + demographics & genetics* [a]	1.87	0.37	1.27	2.77	0.002
Model 3: *Model 2 + Fully Adjusted* [b]	1.50	0.31	1.00	2.25	0.052
Model 2a: *Demographics*	1.77	0.35	1.21	2.61	0.004
Model 2b: *Genetics*	2.11	0.41	1.44	3.07	<0.001
Model 3a: Model 3 + *Medication*	1.50	0.31	1.00	2.25	0.051
Model 3b: Model 3 + *Physical Activity*	1.45	0.30	0.97	2.18	0.074
Model 3c: Model 3 + *Body Mass Index*	1.47	0.31	0.97	2.21	0.069
Model 3d: Model 3 + *Fully Adjusted* [c]	1.43	0.30	0.95	2.15	0.088
Model 4a: Model 2 + *Health*	1.82	0.36	1.23	2.68	0.003
Model 4b: Model 3 + *Health*	1.48	0.31	0.98	2.22	0.060
Model 4c: Model 3 + *Fully Adjusted* [d]	1.42	0.30	0.94	2.13	0.097

Notes: The *low-risk* group is the reference; HR = hazard ratio; SE = standard error; CI = confidence interval; *p* = significance value.

a *Demographic and genetic variables*: age; sex; 10 principal components (PCs); C-reactive Protein (CRP) polygenic score (PGS); White Blood Cell Counts (WBCC) PGS; Insulin Growth Factor-1 (IGF-1) PGS; Anxiety PGS; Depression PGS; Schizophrenia PGS; Insomnia PGS; Pain PGS.

b All variables: age; sex; 10 PCs; CRP PGS; WBCC PGS; IGF-1 PGS; PGS; Anxiety PGS; Depression PGS; Schizophrenia PGS; Insomnia PGS; Pain PGS; education; wealth; smoking status; alcohol consumption; mobility.

c Additional variables: medication, physical activity; BMI.

d Additional variables: health (i.e., chronic lung disease; coronary heart disease; abnormal heart rhythm; heart murmur; congestive heart failure; angina; hypertension; diabetes; cancer; Parkinson's; Alzheimer's; dementia; asthma; arthritis; osteoporosis; psychiatric disorder).

Table S9o. **Longitudinal associations between insulin growth factor-1 and hospitalisation for diseases of the blood and blood-forming organs and certain disorders involving the immune mechanism (*n*=4,139)**

Adjustments	IGF-1				
	**HR**	**SE**	**95% CI**		***p***
**Blood Disorders**					
Model 1: *Unadjusted*	0.61	0.09	0.46	0.81	0.001
Model 2: *Model 1 + demographics & genetics* [a]	0.86	0.13	0.64	1.15	0.304
Model 3: *Model 2 + Fully Adjusted* [b]	0.87	0.13	0.66	1.16	0.349
Model 2a: *Demographics*	0.85	0.12	0.64	1.13	0.255
Model 2b: *Genetics*	0.62	0.09	0.47	0.82	0.001
Model 3a: Model 3 + *Medication*	0.87	0.13	0.66	1.16	0.342
Model 3b: Model 3 + *Physical Activity*	0.89	0.13	0.67	1.17	0.392
Model 3c: Model 3 + *Body Mass Index*	0.88	0.13	0.66	1.16	0.360
Model 3d: Model 3 + *Fully Adjusted* [c]	0.89	0.13	0.67	1.17	0.394
Model 4a: Model 2 + *Health*	0.86	0.13	0.65	1.15	0.309
Model 4b: Model 3 + *Health*	0.87	0.13	0.66	1.16	0.350
Model 4c: Model 3 + *Fully Adjusted* [d]	0.89	0.13	0.67	1.17	0.396

Notes: The *low-risk* group is the reference; HR = hazard ratio; SE = standard error; CI = confidence interval; *p* = significance value.

a *Demographic and genetic variables*: age; sex; 10 principal components (PCs); C-reactive Protein (CRP) polygenic score (PGS); White Blood Cell Counts (WBCC) PGS; Insulin Growth Factor-1 (IGF-1) PGS; Anxiety PGS; Depression PGS; Schizophrenia PGS; Insomnia PGS; Pain PGS.

b All variables: age; sex; 10 PCs; CRP PGS; WBCC PGS; IGF-1 PGS; PGS; Anxiety PGS; Depression PGS; Schizophrenia PGS; Insomnia PGS; Pain PGS; education; wealth; smoking status; alcohol consumption; mobility.

c Additional variables: medication, physical activity; BMI.

d Additional variables: health (i.e., chronic lung disease; coronary heart disease; abnormal heart rhythm; heart murmur; congestive heart failure; angina; hypertension; diabetes; cancer; Parkinson's; Alzheimer's; dementia; asthma; arthritis; osteoporosis; psychiatric disorder).

Table S9p. **Longitudinal associations between immune and neuroendocrine profiles and hospitalisation for diseases of the circulatory system (*n*=3,163)**

Adjustments	Immune and Neuroendocrine Profiles				
	HR	SE	95% CI		*p*
***Moderate-risk* Profile | Circulatory Disorders**					
Model 1: *Unadjusted*	1.49	0.09	1.33	1.67	<0.001
Model 2: *Model 1 + demographics & genetics* [a]	1.34	0.08	1.19	1.50	<0.001
Model 3: *Model 2 + Fully Adjusted* [b]	1.26	0.08	1.12	1.42	<0.001
Model 2a: *Demographics*	1.35	0.08	1.20	1.51	<0.001
Model 2b: *Genetics*	1.48	0.09	1.32	1.66	<0.001
Model 3a: Model 3 + *Medication*	1.27	0.08	1.13	1.42	<0.001
Model 3b: Model 3 + *Physical Activity*	1.25	0.07	1.11	1.40	<0.001
Model 3c: Model 3 + *Body Mass Index*	1.19	0.07	1.05	1.34	0.005
Model 3d: Model 3 + *Fully Adjusted* [c]	1.18	0.07	1.05	1.34	0.006
Model 4a: Model 2 + *Health*	1.49	0.09	1.33	1.67	<0.001
Model 4b: Model 3 + *Health*	1.25	0.07	1.12	1.41	<0.001
Model 4c: Model 3 + *Fully Adjusted* [d]	1.18	0.07	1.04	1.33	0.009
***High-risk* Profile | Circulatory Disorders**					
Model 1: *Unadjusted*	1.72	0.14	1.46	2.03	<0.001
Model 2: *Model 1 + demographics & genetics* [a]	1.51	0.13	1.27	1.78	<0.001
Model 3: *Model 2 + Fully Adjusted* [b]	1.37	0.12	1.16	1.63	<0.001
Model 2a: *Demographics*	1.53	0.13	1.30	1.81	<0.001
Model 2b: *Genetics*	1.69	0.14	1.43	1.99	<0.001
Model 3a: Model 3 + *Medication*	1.37	0.12	1.15	1.62	<0.001
Model 3b: Model 3 + *Physical Activity*	1.35	0.12	1.14	1.60	0.001
Model 3c: Model 3 + *Body Mass Index*	1.28	0.11	1.07	1.52	0.006
Model 3d: Model 3 + *Fully Adjusted* [c]	1.25	0.11	1.05	1.49	0.011
Model 4a: Model 2 + *Health*	1.72	0.14	1.46	2.03	<0.001
Model 4b: Model 3 + *Health*	1.36	0.12	1.15	1.61	<0.001
Model 4c: Model 3 + *Fully Adjusted* [d]	1.25	0.11	1.05	1.48	0.014

Notes: The *low-risk* group is the reference; HR = hazard ratio; SE = standard error; CI = confidence interval; *p* = significance value.

a *Demographic and genetic variables*: age; sex; 10 principal components (PCs); C-reactive Protein (CRP) polygenic score (PGS); White Blood Cell Counts (WBCC) PGS; Insulin Growth Factor-1 (IGF-1) PGS; Anxiety PGS; Depression PGS; Schizophrenia PGS; Insomnia PGS; Pain PGS.

b All variables: age; sex; 10 PCs; CRP PGS; WBCC PGS; IGF-1 PGS; PGS; Anxiety PGS; Depression PGS; Schizophrenia PGS; Insomnia PGS; Pain PGS; education; wealth; smoking status; alcohol consumption; mobility.

c Additional variables: medication, physical activity; BMI.

d Additional variables: health (i.e., chronic lung disease; coronary heart disease; abnormal heart rhythm; heart murmur; congestive heart failure; angina; hypertension; diabetes; cancer; Parkinson's; Alzheimer's; dementia; asthma; arthritis; osteoporosis; psychiatric disorder).

Table S9q. **Longitudinal associations between C-reactive protein and hospitalisation for diseases of the circulatory system (*n*=3,163)**

**Adjustments**	**CRP**				
	**HR**	**SE**	**95% CI**		***p***
**Circulatory Disorders**					
Model 1: *Unadjusted*	1.41	0.05	1.31	1.52	<0.001
Model 2: *Model 1 + demographics & genetics* [a]	1.31	0.05	1.21	1.42	<0.001
Model 3: *Model 2 + Fully Adjusted* [b]	1.25	0.05	1.15	1.36	<0.001
Model 2a: *Demographics*	1.32	0.05	1.22	1.43	<0.001
Model 2b: *Genetics*	1.41	0.05	1.30	1.52	<0.001
Model 3a: Model 3 + *Medication*	1.25	0.05	1.15	1.36	<0.001
Model 3b: Model 3 + *Physical Activity*	1.24	0.05	1.14	1.34	<0.001
Model 3c: Model 3 + *Body Mass Index*	1.20	0.05	1.10	1.30	<0.001
Model 3d: Model 3 + *Fully Adjusted* [c]	1.19	0.05	1.09	1.29	<0.001
Model 4a: Model 2 + *Health*	1.31	0.05	1.21	1.42	<0.001
Model 4b: Model 3 + *Health*	1.24	0.05	1.15	1.35	<0.001
Model 4c: Model 3 + *Fully Adjusted* [d]	1.18	0.05	1.08	1.28	<0.001

Notes: The *low-risk* group is the reference; HR = hazard ratio; SE = standard error; CI = confidence interval; *p* = significance value.

a *Demographic and genetic variables*: age; sex; 10 principal components (PCs); C-reactive Protein (CRP) polygenic score (PGS); White Blood Cell Counts (WBCC) PGS; Insulin Growth Factor-1 (IGF-1) PGS; Anxiety PGS; Depression PGS; Schizophrenia PGS; Insomnia PGS; Pain PGS.

b All variables: age; sex; 10 PCs; CRP PGS; WBCC PGS; IGF-1 PGS; PGS; Anxiety PGS; Depression PGS; Schizophrenia PGS; Insomnia PGS; Pain PGS; education; wealth; smoking status; alcohol consumption; mobility.

c Additional variables: medication, physical activity; BMI.

d Additional variables: health (i.e., chronic lung disease; coronary heart disease; abnormal heart rhythm; heart murmur; congestive heart failure; angina; hypertension; diabetes; cancer; Parkinson's; Alzheimer's; dementia; asthma; arthritis; osteoporosis; psychiatric disorder).

Table S9r. **Longitudinal associations between fibrinogen and hospitalisation for diseases of the circulatory system (*n*=3,163)**

Adjustments	Fb				
	HR	SE	95% CI		*p*
**Circulatory Disorders**					
Model 1: *Unadjusted*	1.42	0.07	1.29	1.57	<0.001
Model 2: *Model 1 + demographics & genetics* [a]	1.29	0.07	1.17	1.43	<0.001
Model 3: *Model 2 + Fully Adjusted* [b]	1.21	0.06	1.10	1.34	<0.001
Model 2a: *Demographics*	1.31	0.07	1.18	1.44	<0.001
Model 2b: *Genetics*	1.40	0.07	1.28	1.55	<0.001
Model 3a: Model 3 + *Medication*	1.21	0.06	1.10	1.34	<0.001
Model 3b: Model 3 + *Physical Activity*	1.20	0.06	1.08	1.33	<0.001
Model 3c: Model 3 + *Body Mass Index*	1.17	0.06	1.05	1.29	0.003
Model 3d: Model 3 + *Fully Adjusted* [c]	1.16	0.06	1.05	1.29	0.004
Model 4a: Model 2 + *Health*	1.29	0.07	1.17	1.42	<0.001
Model 4b: Model 3 + *Health*	1.21	0.06	1.10	1.34	<0.001
Model 4c: Model 3 + *Fully Adjusted* [d]	1.16	0.06	1.05	1.29	0.004

Notes: The *low-risk* group is the reference; HR = hazard ratio; SE = standard error; CI = confidence interval; *p* = significance value.

a *Demographic and genetic variables*: age; sex; 10 principal components (PCs); C-reactive Protein (CRP) polygenic score (PGS); White Blood Cell Counts (WBCC) PGS; Insulin Growth Factor-1 (IGF-1) PGS; Anxiety PGS; Depression PGS; Schizophrenia PGS; Insomnia PGS; Pain PGS.

b All variables: age; sex; 10 PCs; CRP PGS; WBCC PGS; IGF-1 PGS; PGS; Anxiety PGS; Depression PGS; Schizophrenia PGS; Insomnia PGS; Pain PGS; education; wealth; smoking status; alcohol consumption; mobility.

c Additional variables: medication, physical activity; BMI.

d Additional variables: health (i.e., chronic lung disease; coronary heart disease; abnormal heart rhythm; heart murmur; congestive heart failure; angina; hypertension; diabetes; cancer; Parkinson's; Alzheimer's; dementia; asthma; arthritis; osteoporosis; psychiatric disorder).

Table S9s. **Longitudinal associations between white blood cell counts and hospitalisation for diseases of the circulatory system (*n*=3,163)**

**Adjustments**	**WBCC**				
	**HR**	**SE**	**95% CI**		***p***
**Circulatory Disorders**					
Model 1: *Unadjusted*	2.06	0.22	1.67	2.54	<0.001
Model 2: *Model 1 + demographics & genetics* [a]	1.95	0.22	1.56	2.43	<0.001
Model 3: *Model 2 + Fully Adjusted* [b]	1.73	0.20	1.38	2.18	<0.001
Model 2a: *Demographics*	1.94	0.21	1.56	2.41	<0.001
Model 2b: *Genetics*	2.02	0.22	1.64	2.50	<0.001
Model 3a: Model 3 + *Medication*	1.74	0.21	1.39	2.20	<0.001
Model 3b: Model 3 + *Physical Activity*	1.69	0.20	1.34	2.13	<0.001
Model 3c: Model 3 + *Body Mass Index*	1.61	0.19	1.28	2.03	<0.001
Model 3d: Model 3 + *Fully Adjusted* [c]	1.59	0.19	1.26	2.00	<0.001
Model 4a: Model 2 + *Health*	1.88	0.21	1.51	2.35	<0.001
Model 4b: Model 3 + *Health*	1.70	0.20	1.35	2.14	<0.001
Model 4c: Model 3 + *Fully Adjusted* [d]	1.56	0.19	1.24	1.98	<0.001

Notes: The *low-risk* group is the reference; HR = hazard ratio; SE = standard error; CI = confidence interval; *p* = significance value.

a *Demographic and genetic variables*: age; sex; 10 principal components (PCs); C-reactive Protein (CRP) polygenic score (PGS); White Blood Cell Counts (WBCC) PGS; Insulin Growth Factor-1 (IGF-1) PGS; Anxiety PGS; Depression PGS; Schizophrenia PGS; Insomnia PGS; Pain PGS.

b All variables: age; sex; 10 PCs; CRP PGS; WBCC PGS; IGF-1 PGS; PGS; Anxiety PGS; Depression PGS; Schizophrenia PGS; Insomnia PGS; Pain PGS; education; wealth; smoking status; alcohol consumption; mobility.

c Additional variables: medication, physical activity; BMI.

d Additional variables: health (i.e., chronic lung disease; coronary heart disease; abnormal heart rhythm; heart murmur; congestive heart failure; angina; hypertension; diabetes; cancer; Parkinson's; Alzheimer's; dementia; asthma; arthritis; osteoporosis; psychiatric disorder).

Table S9t. **Longitudinal associations between insulin growth factor-1 and hospitalisation for diseases of the circulatory system (*n*=3,163)**

Adjustments	IGF-1				
	HR	SE	95% CI		*p*
**Circulatory Disorders**					
Model 1: *Unadjusted*	0.64	0.05	0.55	0.76	<0.001
Model 2: *Model 1 + demographics & genetics* [a]	0.89	0.08	0.76	1.06	0.190
Model 3: *Model 2 + Fully Adjusted* [b]	0.91	0.08	0.77	1.08	0.273
Model 2a: *Demographics*	0.91	0.08	0.77	1.08	0.272
Model 2b: *Genetics*	0.64	0.05	0.55	0.75	<0.001
Model 3a: Model 3 + *Medication*	0.92	0.08	0.78	1.08	0.298
Model 3b: Model 3 + *Physical Activity*	0.92	0.08	0.78	1.09	0.335
Model 3c: Model 3 + *Body Mass Index*	0.92	0.08	0.78	1.08	0.314
Model 3d: Model 3 + *Fully Adjusted* [c]	0.94	0.08	0.80	1.10	0.426
Model 4a: Model 2 + *Health*	0.90	0.08	0.76	1.06	0.221
Model 4b: Model 3 + *Health*	0.92	0.08	0.78	1.08	0.301
Model 4c: Model 3 + *Fully Adjusted* [d]	0.94	0.08	0.80	1.11	0.453

Notes: The *low-risk* group is the reference; HR = hazard ratio; SE = standard error; CI = confidence interval; *p* = significance value.

a *Demographic and genetic variables*: age; sex; 10 principal components (PCs); C-reactive Protein (CRP) polygenic score (PGS); White Blood Cell Counts (WBCC) PGS; Insulin Growth Factor-1 (IGF-1) PGS; Anxiety PGS; Depression PGS; Schizophrenia PGS; Insomnia PGS; Pain PGS.

b All variables: age; sex; 10 PCs; CRP PGS; WBCC PGS; IGF-1 PGS; PGS; Anxiety PGS; Depression PGS; Schizophrenia PGS; Insomnia PGS; Pain PGS; education; wealth; smoking status; alcohol consumption; mobility.

c Additional variables: medication, physical activity; BMI.

d Additional variables: health (i.e., chronic lung disease; coronary heart disease; abnormal heart rhythm; heart murmur; congestive heart failure; angina; hypertension; diabetes; cancer; Parkinson's; Alzheimer's; dementia; asthma; arthritis; osteoporosis; psychiatric disorder).

Table S9u. **Longitudinal associations between immune and neuroendocrine profiles and hospitalisation for diseases of the digestive system (*n*=3,188)**

Adjustments	Immune and Neuroendocrine Profiles				
	HR	SE	95% CI		*p*
***Moderate-risk* Profile | Digestive Disorders**					
Model 1: *Unadjusted*	1.20	0.08	1.06	1.36	0.004
Model 2: *Model 1 + demographics & genetics* [a]	1.09	0.07	0.96	1.24	0.177
Model 3: *Model 2 + Fully Adjusted* [b]	1.04	0.07	0.92	1.18	0.545
Model 2a: *Demographics*	1.11	0.07	0.98	1.26	0.098
Model 2b: *Genetics*	1.18	0.08	1.04	1.34	0.009
Model 3a: Model 3 + *Medication*	1.04	0.07	0.91	1.18	0.574
Model 3b: Model 3 + *Physical Activity*	1.03	0.07	0.91	1.17	0.632
Model 3c: Model 3 + *Body Mass Index*	1.03	0.07	0.90	1.18	0.655
Model 3d: Model 3 + *Fully Adjusted* [c]	1.02	0.07	0.90	1.17	0.750
Model 4a: Model 2 + *Health*	1.09	0.07	0.96	1.24	0.177
Model 4b: Model 3 + *Health*	1.03	0.07	0.91	1.17	0.620
Model 4c: Model 3 + *Fully Adjusted* [d]	1.02	0.07	0.89	1.16	0.810
***High-risk* Profile | Digestive Disorders**					
Model 1: *Unadjusted*	1.12	0.11	0.93	1.36	0.236
Model 2: *Model 1 + demographics & genetics* [a]	1.00	0.10	0.82	1.22	0.998
Model 3: *Model 2 + Fully Adjusted* [b]	0.93	0.09	0.76	1.13	0.460
Model 2a: *Demographics*	1.03	0.10	0.85	1.26	0.733
Model 2b: *Genetics*	1.09	0.11	0.89	1.32	0.409
Model 3a: Model 3 + *Medication*	0.93	0.10	0.76	1.14	0.472
Model 3b: Model 3 + *Physical Activity*	0.91	0.09	0.75	1.12	0.376
Model 3c: Model 3 + *Body Mass Index*	0.92	0.10	0.75	1.13	0.407
Model 3d: Model 3 + *Fully Adjusted* [c]	0.91	0.10	0.74	1.11	0.354
Model 4a: Model 2 + *Health*	1.00	0.10	0.82	1.22	0.998
Model 4b: Model 3 + *Health*	0.92	0.09	0.75	1.12	0.404
Model 4c: Model 3 + *Fully Adjusted* [d]	0.90	0.09	0.74	1.11	0.322

Notes: The *low-risk* group is the reference; HR = hazard ratio; SE = standard error; CI = confidence interval; *p* = significance value.

a *Demographic and genetic variables*: age; sex; 10 principal components (PCs); C-reactive Protein (CRP) polygenic score (PGS); White Blood Cell Counts (WBCC) PGS; Insulin Growth Factor-1 (IGF-1) PGS; Anxiety PGS; Depression PGS; Schizophrenia PGS; Insomnia PGS; Pain PGS.

b All variables: age; sex; 10 PCs; CRP PGS; WBCC PGS; IGF-1 PGS; PGS; Anxiety PGS; Depression PGS; Schizophrenia PGS; Insomnia PGS; Pain PGS; education; wealth; smoking status; alcohol consumption; mobility.

c Additional variables: medication, physical activity; BMI.

d Additional variables: health (i.e., chronic lung disease; coronary heart disease; abnormal heart rhythm; heart murmur; congestive heart failure; angina; hypertension; diabetes; cancer; Parkinson's; Alzheimer's; dementia; asthma; arthritis; osteoporosis; psychiatric disorder).

Table S9v. **Longitudinal associations between C-reactive protein and hospitalisation for diseases of the digestive system (*n*=3,188)**

**Adjustments**	**CRP**				
	**HR**	**SE**	**95% CI**		***p***
**Digestive Disorders**					
Model 1: *Unadjusted*	1.14	0.05	1.05	1.24	0.002
Model 2: *Model 1 + demographics & genetics* [a]	1.06	0.05	0.97	1.16	0.185
Model 3: *Model 2 + Fully Adjusted* [b]	1.02	0.05	0.94	1.12	0.620
Model 2a: *Demographics*	1.08	0.05	0.99	1.17	0.096
Model 2b: *Genetics*	1.13	0.05	1.04	1.23	0.006
Model 3a: Model 3 + *Medication*	1.02	0.05	0.94	1.12	0.623
Model 3b: Model 3 + *Physical Activity*	1.02	0.05	0.93	1.11	0.750
Model 3c: Model 3 + *Body Mass Index*	1.02	0.05	0.93	1.12	0.725
Model 3d: Model 3 + *Fully Adjusted* [c]	1.01	0.05	0.92	1.11	0.827
Model 4a: Model 2 + *Health*	1.06	0.05	0.97	1.16	0.185
Model 4b: Model 3 + *Health*	1.02	0.05	0.93	1.11	0.747
Model 4c: Model 3 + *Fully Adjusted* [d]	1.00	0.05	0.91	1.10	0.933

Notes: The *low-risk* group is the reference; HR = hazard ratio; SE = standard error; CI = confidence interval; *p* = significance value.

a *Demographic and genetic variables*: age; sex; 10 principal components (PCs); C-reactive Protein (CRP) polygenic score (PGS); White Blood Cell Counts (WBCC) PGS; Insulin Growth Factor-1 (IGF-1) PGS; Anxiety PGS; Depression PGS; Schizophrenia PGS; Insomnia PGS; Pain PGS.

b All variables: age; sex; 10 PCs; CRP PGS; WBCC PGS; IGF-1 PGS; PGS; Anxiety PGS; Depression PGS; Schizophrenia PGS; Insomnia PGS; Pain PGS; education; wealth; smoking status; alcohol consumption; mobility.

c Additional variables: medication, physical activity; BMI.

d Additional variables: health (i.e., chronic lung disease; coronary heart disease; abnormal heart rhythm; heart murmur; congestive heart failure; angina; hypertension; diabetes; cancer; Parkinson's; Alzheimer's; dementia; asthma; arthritis; osteoporosis; psychiatric disorder).

Table S9w. **Longitudinal associations between fibrinogen and hospitalisation for diseases of the digestive system (*n*=3,188)**

**Adjustments**	**Fb**				
	**HR**	**SE**	**95% CI**		***p***
**Digestive Disorders**					
Model 1: *Unadjusted*	1.11	0.06	1.00	1.23	0.045
Model 2: *Model 1 + demographics & genetics* [a]	1.03	0.06	0.92	1.14	0.609
Model 3: *Model 2 + Fully Adjusted* [b]	0.97	0.05	0.87	1.08	0.580
Model 2a: *Demographics*	1.05	0.06	0.94	1.16	0.415
Model 2b: *Genetics*	1.09	0.06	0.98	1.21	0.106
Model 3a: Model 3 + *Medication*	0.97	0.05	0.87	1.08	0.568
Model 3b: Model 3 + *Physical Activity*	0.97	0.05	0.87	1.08	0.527
Model 3c: Model 3 + *Body Mass Index*	0.96	0.06	0.86	1.08	0.515
Model 3d: Model 3 + *Fully Adjusted* [c]	0.96	0.06	0.86	1.07	0.471
Model 4a: Model 2 + *Health*	1.02	0.06	0.92	1.14	0.686
Model 4b: Model 3 + *Health*	0.97	0.05	0.87	1.08	0.582
Model 4c: Model 3 + *Fully Adjusted* [d]	0.96	0.06	0.86	1.08	0.487

Notes: The *low-risk* group is the reference; HR = hazard ratio; SE = standard error; CI = confidence interval; *p* = significance value.

a *Demographic and genetic variables*: age; sex; 10 principal components (PCs); C-reactive Protein (CRP) polygenic score (PGS); White Blood Cell Counts (WBCC) PGS; Insulin Growth Factor-1 (IGF-1) PGS; Anxiety PGS; Depression PGS; Schizophrenia PGS; Insomnia PGS; Pain PGS.

b All variables: age; sex; 10 PCs; CRP PGS; WBCC PGS; IGF-1 PGS; PGS; Anxiety PGS; Depression PGS; Schizophrenia PGS; Insomnia PGS; Pain PGS; education; wealth; smoking status; alcohol consumption; mobility.

c Additional variables: medication, physical activity; BMI.

d Additional variables: health (i.e., chronic lung disease; coronary heart disease; abnormal heart rhythm; heart murmur; congestive heart failure; angina; hypertension; diabetes; cancer; Parkinson's; Alzheimer's; dementia; asthma; arthritis; osteoporosis; psychiatric disorder).

Table S9x. **Longitudinal associations between white blood cell counts and hospitalisation for diseases of the digestive system (*n*=3,188)**

**Adjustments**	**WBCC**				
	**HR**	**SE**	**95% CI**		***p***
**Digestive Disorders**					
Model 1: *Unadjusted*	1.27	0.15	1.00	1.60	0.046
Model 2: *Model 1 + demographics & genetics* [a]	1.15	0.14	0.90	1.46	0.262
Model 3: *Model 2 + Fully Adjusted* [b]	1.01	0.13	0.78	1.29	0.962
Model 2a: *Demographics*	1.14	0.14	0.90	1.45	0.272
Model 2b: *Genetics*	1.23	0.15	0.98	1.56	0.079
Model 3a: Model 3 + *Medication*	1.00	0.13	0.78	1.29	0.992
Model 3b: Model 3 + *Physical Activity*	0.99	0.13	0.77	1.28	0.961
Model 3c: Model 3 + *Body Mass Index*	1.00	0.13	0.77	1.28	0.969
Model 3d: Model 3 + *Fully Adjusted* [c]	0.98	0.13	0.76	1.27	0.885
Model 4a: Model 2 + *Health*	1.11	0.14	0.87	1.41	0.395
Model 4b: Model 3 + *Health*	0.99	0.13	0.77	1.27	0.913
Model 4c: Model 3 + *Fully Adjusted* [d]	0.96	0.13	0.75	1.25	0.781

Notes: The *low-risk* group is the reference; HR = hazard ratio; SE = standard error; CI = confidence interval; *p* = significance value.

a *Demographic and genetic variables*: age; sex; 10 principal components (PCs); C-reactive Protein (CRP) polygenic score (PGS); White Blood Cell Counts (WBCC) PGS; Insulin Growth Factor-1 (IGF-1) PGS; Anxiety PGS; Depression PGS; Schizophrenia PGS; Insomnia PGS; Pain PGS.

b All variables: age; sex; 10 PCs; CRP PGS; WBCC PGS; IGF-1 PGS; PGS; Anxiety PGS; Depression PGS; Schizophrenia PGS; Insomnia PGS; Pain PGS; education; wealth; smoking status; alcohol consumption; mobility.

c Additional variables: medication, physical activity; BMI.

d Additional variables: health (i.e., chronic lung disease; coronary heart disease; abnormal heart rhythm; heart murmur; congestive heart failure; angina; hypertension; diabetes; cancer; Parkinson's; Alzheimer's; dementia; asthma; arthritis; osteoporosis; psychiatric disorder).

Table S9y. **Longitudinal associations between insulin growth factor-1 and hospitalisation for diseases of the digestive system (*n*=3,188)**

Adjustments	IGF-1				
	HR	SE	95% CI		*p*
**Digestive Disorders**					
Model 1: *Unadjusted*	0.72	0.07	0.61	0.86	<0.001
Model 2: *Model 1 + demographics & genetics* [a]	1.15	0.14	0.90	1.46	0.262
Model 3: *Model 2 + Fully Adjusted* [b]	1.01	0.13	0.78	1.29	0.962
Model 2a: *Demographics*	1.14	0.14	0.90	1.45	0.272
Model 2b: *Genetics*	1.23	0.15	0.98	1.56	0.079
Model 3a: Model 3 + *Medication*	1.00	0.13	0.78	1.29	0.992
Model 3b: Model 3 + *Physical Activity*	0.99	0.13	0.77	1.28	0.961
Model 3c: Model 3 + *Body Mass Index*	1.00	0.13	0.77	1.28	0.969
Model 3d: Model 3 + *Fully Adjusted* [c]	0.98	0.13	0.76	1.27	0.885
Model 4a: Model 2 + *Health*	1.11	0.14	0.87	1.41	0.395
Model 4b: Model 3 + *Health*	0.99	0.13	0.77	1.27	0.913
Model 4c: Model 3 + *Fully Adjusted* [d]	0.96	0.13	0.75	1.25	0.781

Notes: The *low-risk* group is the reference; HR = hazard ratio; SE = standard error; CI = confidence interval; *p* = significance value.

a *Demographic and genetic variables*: age; sex; 10 principal components (PCs); C-reactive Protein (CRP) polygenic score (PGS); White Blood Cell Counts (WBCC) PGS; Insulin Growth Factor-1 (IGF-1) PGS; Anxiety PGS; Depression PGS; Schizophrenia PGS; Insomnia PGS; Pain PGS.

b All variables: age; sex; 10 PCs; CRP PGS; WBCC PGS; IGF-1 PGS; PGS; Anxiety PGS; Depression PGS; Schizophrenia PGS; Insomnia PGS; Pain PGS; education; wealth; smoking status; alcohol consumption; mobility.

c Additional variables: medication, physical activity; BMI.

d Additional variables: health (i.e., chronic lung disease; coronary heart disease; abnormal heart rhythm; heart murmur; congestive heart failure; angina; hypertension; diabetes; cancer; Parkinson's; Alzheimer's; dementia; asthma; arthritis; osteoporosis; psychiatric disorder).

Table S9z. **Longitudinal associations between immune and neuroendocrine profiles and hospitalisation for endocrine, nutritional and metabolic diseases (*n*=3,749)**

**Adjustments**	**Immune and Neuroendocrine Profiles**				
	**HR**	**SE**	**95% CI**		***p***
***Moderate-risk* Profile | Endocrine Disorders**					
Model 1: *Unadjusted*	1.57	0.10	1.39	1.78	<0.001
Model 2: *Model 1 + demographics & genetics* [a]	1.41	0.09	1.24	1.60	<0.001
Model 3: *Model 2 + Fully Adjusted* [b]	1.26	0.08	1.11	1.43	<0.001
Model 2a: *Demographics*	1.41	0.09	1.25	1.60	<0.001
Model 2b: *Genetics*	1.57	0.10	1.39	1.78	<0.001
Model 3a: Model 3 + *Medication*	1.26	0.08	1.11	1.43	<0.001
Model 3b: Model 3 + *Physical Activity*	1.24	0.08	1.09	1.41	0.001
Model 3c: Model 3 + *Body Mass Index*	1.14	0.08	1.00	1.30	0.044
Model 3d: Model 3 + *Fully Adjusted* [c]	1.13	0.08	0.99	1.29	0.070
Model 4a: Model 2 + *Health*	1.41	0.09	1.24	1.60	<0.001
Model 4b: Model 3 + *Health*	1.25	0.08	1.10	1.42	0.001
Model 4c: Model 3 + *Fully Adjusted* [d]	1.13	0.08	0.99	1.28	0.077
***High-risk* Profile | Endocrine Disorders**					
Model 1: *Unadjusted*	1.81	0.16	1.52	2.16	<0.001
Model 2: *Model 1 + demographics & genetics* [a]	1.62	0.15	1.36	1.93	<0.001
Model 3: *Model 2 + Fully Adjusted* [b]	1.38	0.13	1.16	1.66	<0.001
Model 2a: *Demographics*	1.62	0.15	1.35	1.93	<0.001
Model 2b: *Genetics*	1.81	0.16	1.52	2.16	<0.001
Model 3a: Model 3 + *Medication*	1.38	0.13	1.16	1.66	<0.001
Model 3b: Model 3 + *Physical Activity*	1.33	0.12	1.11	1.59	0.002
Model 3c: Model 3 + *Body Mass Index*	1.23	0.12	1.03	1.48	0.025
Model 3d: Model 3 + *Fully Adjusted* [c]	1.19	0.11	0.99	1.43	0.066
Model 4a: Model 2 + *Health*	1.62	0.15	1.36	1.93	<0.001
Model 4b: Model 3 + *Health*	1.38	0.13	1.15	1.65	<0.001
Model 4c: Model 3 + *Fully Adjusted* [d]	1.19	0.11	0.99	1.43	0.070

Notes: The *low-risk* group is the reference; HR = hazard ratio; SE = standard error; CI = confidence interval; *p* = significance value.

a *Demographic and genetic variables*: age; sex; 10 principal components (PCs); C-reactive Protein (CRP) polygenic score (PGS); White Blood Cell Counts (WBCC) PGS; Insulin Growth Factor-1 (IGF-1) PGS; Anxiety PGS; Depression PGS; Schizophrenia PGS; Insomnia PGS; Pain PGS.

b All variables: age; sex; 10 PCs; CRP PGS; WBCC PGS; IGF-1 PGS; PGS; Anxiety PGS; Depression PGS; Schizophrenia PGS; Insomnia PGS; Pain PGS; education; wealth; smoking status; alcohol consumption; mobility.

c Additional variables: medication, physical activity; BMI.

d Additional variables: health (i.e., chronic lung disease; coronary heart disease; abnormal heart rhythm; heart murmur; congestive heart failure; angina; hypertension; diabetes; cancer; Parkinson's; Alzheimer's; dementia; asthma; arthritis; osteoporosis; psychiatric disorder).

Table S9aa. **Longitudinal associations between C-reactive protein and hospitalisation for endocrine, nutritional and metabolic diseases (*n*=3,749)**

Adjustments	CRP				
	HR	SE	95% CI		*p*
**Endocrine Disorders**					
Model 1: *Unadjusted*	1.44	0.06	1.33	1.56	<0.001
Model 2: *Model 1 + demographics & genetics* [a]	1.35	0.06	1.24	1.47	<0.001
Model 3: *Model 2 + Fully Adjusted* [b]	1.24	0.05	1.13	1.35	<0.001
Model 2a: *Demographics*	1.35	0.06	1.24	1.46	<0.001
Model 2b: *Genetics*	1.44	0.06	1.33	1.56	<0.001
Model 3a: Model 3 + *Medication*	1.24	0.05	1.13	1.35	<0.001
Model 3b: Model 3 + *Physical Activity*	1.21	0.05	1.11	1.32	<0.001
Model 3c: Model 3 + *Body Mass Index*	1.15	0.05	1.05	1.26	0.003
Model 3d: Model 3 + *Fully Adjusted* [c]	1.13	0.05	1.03	1.23	0.012
Model 4a: Model 2 + *Health*	1.35	0.06	1.24	1.47	<0.001
Model 4b: Model 3 + *Health*	1.23	0.05	1.13	1.34	<0.001
Model 4c: Model 3 + *Fully Adjusted* [d]	1.12	0.05	1.03	1.23	0.013

Notes: The *low-risk* group is the reference; HR = hazard ratio; SE = standard error; CI = confidence interval; *p* = significance value.

a *Demographic and genetic variables*: age; sex; 10 principal components (PCs); C-reactive Protein (CRP) polygenic score (PGS); White Blood Cell Counts (WBCC) PGS; Insulin Growth Factor-1 (IGF-1) PGS; Anxiety PGS; Depression PGS; Schizophrenia PGS; Insomnia PGS; Pain PGS.

b All variables: age; sex; 10 PCs; CRP PGS; WBCC PGS; IGF-1 PGS; PGS; Anxiety PGS; Depression PGS; Schizophrenia PGS; Insomnia PGS; Pain PGS; education; wealth; smoking status; alcohol consumption; mobility.

c Additional variables: medication, physical activity; BMI.

d Additional variables: health (i.e., chronic lung disease; coronary heart disease; abnormal heart rhythm; heart murmur; congestive heart failure; angina; hypertension; diabetes; cancer; Parkinson's; Alzheimer's; dementia; asthma; arthritis; osteoporosis; psychiatric disorder).

Table S9ab. **Longitudinal associations between fibrinogen and hospitalisation for endocrine, nutritional and metabolic diseases (*n*=3,749)**

**Adjustments**	**Fb**				
	**HR**	**SE**	**95% CI**		***p***
**Endocrine Disorders**					
Model 1: *Unadjusted*	1.47	0.07	1.33	1.62	<0.001
Model 2: *Model 1 + demographics & genetics* [a]	1.36	0.07	1.23	1.51	<0.001
Model 3: *Model 2 + Fully Adjusted* [b]	1.23	0.07	1.10	1.36	<0.001
Model 2a: *Demographics*	1.35	0.07	1.22	1.50	<0.001
Model 2b: *Genetics*	1.47	0.08	1.33	1.62	<0.001
Model 3a: Model 3 + *Medication*	1.23	0.07	1.10	1.36	<0.001
Model 3b: Model 3 + *Physical Activity*	1.20	0.07	1.08	1.34	0.001
Model 3c: Model 3 + *Body Mass Index*	1.16	0.06	1.04	1.30	0.006
Model 3d: Model 3 + *Fully Adjusted* [c]	1.15	0.06	1.03	1.28	0.013
Model 4a: Model 2 + *Health*	1.35	0.07	1.22	1.50	<0.001
Model 4b: Model 3 + *Health*	1.22	0.07	1.10	1.36	<0.001
Model 4c: Model 3 + *Fully Adjusted* [d]	1.15	0.06	1.03	1.28	0.013

Notes: The *low-risk* group is the reference; HR = hazard ratio; SE = standard error; CI = confidence interval; *p* = significance value.

a *Demographic and genetic variables*: age; sex; 10 principal components (PCs); C-reactive Protein (CRP) polygenic score (PGS); White Blood Cell Counts (WBCC) PGS; Insulin Growth Factor-1 (IGF-1) PGS; Anxiety PGS; Depression PGS; Schizophrenia PGS; Insomnia PGS; Pain PGS.

b All variables: age; sex; 10 PCs; CRP PGS; WBCC PGS; IGF-1 PGS; PGS; Anxiety PGS; Depression PGS; Schizophrenia PGS; Insomnia PGS; Pain PGS; education; wealth; smoking status; alcohol consumption; mobility.

c Additional variables: medication, physical activity; BMI.

d Additional variables: health (i.e., chronic lung disease; coronary heart disease; abnormal heart rhythm; heart murmur; congestive heart failure; angina; hypertension; diabetes; cancer; Parkinson's; Alzheimer's; dementia; asthma; arthritis; osteoporosis; psychiatric disorder).

Table S9ac. **Longitudinal associations between white blood cell counts and hospitalisation for endocrine, nutritional and metabolic diseases (*n*=3,749)**

Adjustments	WBCC				
	HR	SE	95% CI		*p*
**Endocrine Disorders**					
Model 1: *Unadjusted*	2.11	0.25	1.68	2.66	<0.001
Model 2: *Model 1 + demographics & genetics* [a]	2.02	0.24	1.60	2.56	<0.001
Model 3: *Model 2 + Fully Adjusted* [b]	1.54	0.19	1.20	1.97	0.001
Model 2a: *Demographics*	2.00	0.24	1.58	2.53	<0.001
Model 2b: *Genetics*	2.11	0.25	1.68	2.66	<0.001
Model 3a: Model 3 + *Medication*	1.54	0.19	1.20	1.97	0.001
Model 3b: Model 3 + *Physical Activity*	1.50	0.19	1.17	1.92	0.001
Model 3c: Model 3 + *Body Mass Index*	1.36	0.18	1.06	1.75	0.015
Model 3d: Model 3 + *Fully Adjusted* [c]	1.34	0.17	1.04	1.72	0.023
Model 4a: Model 2 + *Health*	1.98	0.24	1.56	2.51	<0.001
Model 4b: Model 3 + *Health*	1.53	0.19	1.19	1.95	0.001
Model 4c: Model 3 + *Fully Adjusted* [d]	1.33	0.17	1.04	1.71	0.025

Notes: The *low-risk* group is the reference; HR = hazard ratio; SE = standard error; CI = confidence interval; *p* = significance value.

a *Demographic and genetic variables*: age; sex; 10 principal components (PCs); C-reactive Protein (CRP) polygenic score (PGS); White Blood Cell Counts (WBCC) PGS; Insulin Growth Factor-1 (IGF-1) PGS; Anxiety PGS; Depression PGS; Schizophrenia PGS; Insomnia PGS; Pain PGS.

b All variables: age; sex; 10 PCs; CRP PGS; WBCC PGS; IGF-1 PGS; PGS; Anxiety PGS; Depression PGS; Schizophrenia PGS; Insomnia PGS; Pain PGS; education; wealth; smoking status; alcohol consumption; mobility.

c Additional variables: medication, physical activity; BMI.

d Additional variables: health (i.e., chronic lung disease; coronary heart disease; abnormal heart rhythm; heart murmur; congestive heart failure; angina; hypertension; diabetes; cancer; Parkinson's; Alzheimer's; dementia; asthma; arthritis; osteoporosis; psychiatric disorder).

Table S9ad. **Longitudinal associations between insulin growth factor-1 and hospitalisation for endocrine, nutritional and metabolic diseases (*n*=3,749)**

Adjustments	IGF-1				
	HR	SE	95% CI		*p*
**Endocrine Disorders**					
Model 1: *Unadjusted*	0.65	0.06	0.54	0.77	<0.001
Model 2: *Model 1 + demographics & genetics* [a]	0.85	0.08	0.71	1.02	0.075
Model 3: *Model 2 + Fully Adjusted* [b]	0.87	0.08	0.73	1.03	0.106
Model 2a: *Demographics*	0.85	0.08	0.71	1.02	0.073
Model 2b: *Genetics*	0.65	0.06	0.54	0.77	<0.001
Model 3a: Model 3 + *Medication*	0.87	0.08	0.73	1.03	0.105
Model 3b: Model 3 + *Physical Activity*	0.87	0.08	0.73	1.04	0.132
Model 3c: Model 3 + *Body Mass Index*	0.87	0.08	0.73	1.04	0.123
Model 3d: Model 3 + *Fully Adjusted* [c]	0.88	0.08	0.74	1.05	0.159
Model 4a: Model 2 + *Health*	0.85	0.08	0.71	1.01	0.068
Model 4b: Model 3 + *Health*	0.86	0.08	0.72	1.03	0.100
Model 4c: Model 3 + *Fully Adjusted* [d]	0.88	0.08	0.74	1.05	0.153

Notes: The *low-risk* group is the reference; HR = hazard ratio; SE = standard error; CI = confidence interval; *p* = significance value.

a *Demographic and genetic variables*: age; sex; 10 principal components (PCs); C-reactive Protein (CRP) polygenic score (PGS); White Blood Cell Counts (WBCC) PGS; Insulin Growth Factor-1 (IGF-1) PGS; Anxiety PGS; Depression PGS; Schizophrenia PGS; Insomnia PGS; Pain PGS.

b All variables: age; sex; 10 PCs; CRP PGS; WBCC PGS; IGF-1 PGS; PGS; Anxiety PGS; Depression PGS; Schizophrenia PGS; Insomnia PGS; Pain PGS; education; wealth; smoking status; alcohol consumption; mobility.

c Additional variables: medication, physical activity; BMI.

d Additional variables: health (i.e., chronic lung disease; coronary heart disease; abnormal heart rhythm; heart murmur; congestive heart failure; angina; hypertension; diabetes; cancer; Parkinson's; Alzheimer's; dementia; asthma; arthritis; osteoporosis; psychiatric disorder).

Table S9ae. **Longitudinal associations between immune and neuroendocrine profiles and hospitalisation for diseases of the genitourinary system (*n*=3,524)**

**Adjustments**	**Immune and Neuroendocrine Profiles**				
	**HR**	**SE**	**95% CI**		***p***
***Moderate-risk* Profile | Genitourinary Disorders**					
Model 1: *Unadjusted*	1.45	0.10	1.26	1.67	<0.001
Model 2: *Model 1 + demographics & genetics* [a]	1.28	0.09	1.11	1.48	0.001
Model 3: *Model 2 + Fully Adjusted* [b]	1.18	0.09	1.02	1.36	0.027
Model 2a: *Demographics*	1.27	0.09	1.10	1.46	0.001
Model 2b: *Genetics*	1.45	0.10	1.26	1.67	<0.001
Model 3a: Model 3 + *Medication*	1.18	0.09	1.02	1.36	0.028
Model 3b: Model 3 + *Physical Activity*	1.16	0.09	1.01	1.34	0.042
Model 3c: Model 3 + *Body Mass Index*	1.12	0.09	0.97	1.30	0.131
Model 3d: Model 3 + *Fully Adjusted* [c]	1.11	0.09	0.96	1.29	0.163
Model 4a: Model 2 + *Health*	1.27	0.09	1.10	1.46	0.001
Model 4b: Model 3 + *Health*	1.17	0.09	1.01	1.36	0.032
Model 4c: Model 3 + *Fully Adjusted* [d]	1.11	0.09	0.95	1.29	0.180
***High-risk* Profile | Genitourinary Disorders**					
Model 1: *Unadjusted*	1.68	0.17	1.37	2.05	<0.001
Model 2: *Model 1 + demographics & genetics* [a]	1.50	0.16	1.22	1.84	<0.001
Model 3: *Model 2 + Fully Adjusted* [b]	1.35	0.14	1.10	1.65	0.004
Model 2a: *Demographics*	1.49	0.15	1.22	1.83	<0.001
Model 2b: *Genetics*	1.66	0.17	1.36	2.03	<0.001
Model 3a: Model 3 + *Medication*	1.35	0.14	1.10	1.66	0.004
Model 3b: Model 3 + *Physical Activity*	1.29	0.14	1.05	1.59	0.014
Model 3c: Model 3 + *Body Mass Index*	1.28	0.14	1.04	1.58	0.022
Model 3d: Model 3 + *Fully Adjusted* [c]	1.24	0.13	1.00	1.53	0.048
Model 4a: Model 2 + *Health*	1.49	0.15	1.21	1.82	<0.001
Model 4b: Model 3 + *Health*	1.34	0.14	1.09	1.65	0.005
Model 4c: Model 3 + *Fully Adjusted* [d]	1.23	0.13	1.00	1.52	0.050

Notes: The *low-risk* group is the reference; HR = hazard ratio; SE = standard error; CI = confidence interval; *p* = significance value.

a *Demographic and genetic variables*: age; sex; 10 principal components (PCs); C-reactive Protein (CRP) polygenic score (PGS); White Blood Cell Counts (WBCC) PGS; Insulin Growth Factor-1 (IGF-1) PGS; Anxiety PGS; Depression PGS; Schizophrenia PGS; Insomnia PGS; Pain PGS.

b All variables: age; sex; 10 PCs; CRP PGS; WBCC PGS; IGF-1 PGS; PGS; Anxiety PGS; Depression PGS; Schizophrenia PGS; Insomnia PGS; Pain PGS; education; wealth; smoking status; alcohol consumption; mobility.

c Additional variables: medication, physical activity; BMI.

d Additional variables: health (i.e., chronic lung disease; coronary heart disease; abnormal heart rhythm; heart murmur; congestive heart failure; angina; hypertension; diabetes; cancer; Parkinson's; Alzheimer's; dementia; asthma; arthritis; osteoporosis; psychiatric disorder).

Table S9af. **Longitudinal associations between C-reactive protein and hospitalisation for diseases of the genitourinary system (*n*=3,524)**

**Adjustments**	**CRP**				
	**HR**	**SE**	**95% CI**		***p***
**Genitourinary Disorders**					
Model 1: *Unadjusted*	1.35	0.06	1.23	1.48	<0.001
Model 2: *Model 1 + demographics & genetics* [a]	1.26	0.06	1.14	1.39	<0.001
Model 3: *Model 2 + Fully Adjusted* [b]	1.17	0.06	1.06	1.30	0.002
Model 2a: *Demographics*	1.25	0.06	1.14	1.38	<0.001
Model 2b: *Genetics*	1.34	0.06	1.22	1.48	<0.001
Model 3a: Model 3 + *Medication*	1.18	0.06	1.06	1.30	0.001
Model 3b: Model 3 + *Physical Activity*	1.15	0.06	1.04	1.27	0.007
Model 3c: Model 3 + *Body Mass Index*	1.13	0.06	1.02	1.26	0.018
Model 3d: Model 3 + *Fully Adjusted* [c]	1.11	0.06	1.00	1.24	0.044
Model 4a: Model 2 + *Health*	1.25	0.06	1.13	1.38	<0.001
Model 4b: Model 3 + *Health*	1.17	0.06	1.06	1.29	0.002
Model 4c: Model 3 + *Fully Adjusted* [d]	1.11	0.06	1.00	1.23	0.049

Notes: The *low-risk* group is the reference; HR = hazard ratio; SE = standard error; CI = confidence interval; *p* = significance value.

a *Demographic and genetic variables*: age; sex; 10 principal components (PCs); C-reactive Protein (CRP) polygenic score (PGS); White Blood Cell Counts (WBCC) PGS; Insulin Growth Factor-1 (IGF-1) PGS; Anxiety PGS; Depression PGS; Schizophrenia PGS; Insomnia PGS; Pain PGS.

b All variables: age; sex; 10 PCs; CRP PGS; WBCC PGS; IGF-1 PGS; PGS; Anxiety PGS; Depression PGS; Schizophrenia PGS; Insomnia PGS; Pain PGS; education; wealth; smoking status; alcohol consumption; mobility.

c Additional variables: medication, physical activity; BMI.

d Additional variables: health (i.e., chronic lung disease; coronary heart disease; abnormal heart rhythm; heart murmur; congestive heart failure; angina; hypertension; diabetes; cancer; Parkinson's; Alzheimer's; dementia; asthma; arthritis; osteoporosis; psychiatric disorder).

Table S9ag. **Longitudinal associations between fibrinogen and hospitalisation for diseases of the genitourinary system (*n*=3,524)**

**Adjustments**	**Fb**				
	**HR**	**SE**	**95% CI**		***p***
**Genitourinary Disorders**					
Model 1: *Unadjusted*	1.37	0.08	1.22	1.53	<0.001
Model 2: *Model 1 + demographics & genetics* [a]	1.26	0.08	1.12	1.42	<0.001
Model 3: *Model 2 + Fully Adjusted* [b]	1.18	0.07	1.04	1.33	0.009
Model 2a: *Demographics*	1.26	0.08	1.12	1.42	<0.001
Model 2b: *Genetics*	1.37	0.08	1.22	1.54	<0.001
Model 3a: Model 3 + *Medication*	1.18	0.07	1.04	1.33	0.009
Model 3b: Model 3 + *Physical Activity*	1.16	0.07	1.03	1.31	0.017
Model 3c: Model 3 + *Body Mass Index*	1.15	0.07	1.02	1.30	0.029
Model 3d: Model 3 + *Fully Adjusted* [c]	1.14	0.07	1.01	1.29	0.041
Model 4a: Model 2 + *Health*	1.26	0.08	1.12	1.42	<0.001
Model 4b: Model 3 + *Health*	1.18	0.07	1.04	1.33	0.009
Model 4c: Model 3 + *Fully Adjusted* [d]	1.14	0.07	1.01	1.29	0.041

Notes: The *low-risk* group is the reference; HR = hazard ratio; SE = standard error; CI = confidence interval; *p* = significance value.

a *Demographic and genetic variables*: age; sex; 10 principal components (PCs); C-reactive Protein (CRP) polygenic score (PGS); White Blood Cell Counts (WBCC) PGS; Insulin Growth Factor-1 (IGF-1) PGS; Anxiety PGS; Depression PGS; Schizophrenia PGS; Insomnia PGS; Pain PGS.

b All variables: age; sex; 10 PCs; CRP PGS; WBCC PGS; IGF-1 PGS; PGS; Anxiety PGS; Depression PGS; Schizophrenia PGS; Insomnia PGS; Pain PGS; education; wealth; smoking status; alcohol consumption; mobility.

c Additional variables: medication, physical activity; BMI.

d Additional variables: health (i.e., chronic lung disease; coronary heart disease; abnormal heart rhythm; heart murmur; congestive heart failure; angina; hypertension; diabetes; cancer; Parkinson's; Alzheimer's; dementia; asthma; arthritis; osteoporosis; psychiatric disorder).

Table S9ah. **Longitudinal associations between white blood cell counts and hospitalisation for diseases of the genitourinary system (*n*=3,524)**

Adjustments	WBCC				
	HR	SE	95% CI		*p*
**Genitourinary Disorders**					
Model 1: *Unadjusted*	1.92	0.26	1.47	2.49	<0.001
Model 2: *Model 1 + demographics & genetics* [a]	1.75	0.25	1.33	2.30	<0.001
Model 3: *Model 2 + Fully Adjusted* [b]	1.54	0.22	1.16	2.05	0.003
Model 2a: *Demographics*	1.71	0.24	1.31	2.24	<0.001
Model 2b: *Genetics*	1.93	0.26	1.48	2.52	<0.001
Model 3a: Model 3 + *Medication*	1.54	0.22	1.16	2.05	0.003
Model 3b: Model 3 + *Physical Activity*	1.49	0.22	1.12	1.98	0.006
Model 3c: Model 3 + *Body Mass Index*	1.46	0.22	1.10	1.95	0.010
Model 3d: Model 3 + *Fully Adjusted* [c]	1.43	0.21	1.07	1.90	0.016
Model 4a: Model 2 + *Health*	1.72	0.24	1.31	2.26	<0.001
Model 4b: Model 3 + *Health*	1.52	0.22	1.15	2.03	0.004
Model 4c: Model 3 + *Fully Adjusted* [d]	1.42	0.21	1.06	1.89	0.018

Notes: The *low-risk* group is the reference; HR = hazard ratio; SE = standard error; CI = confidence interval; *p* = significance value.

a *Demographic and genetic variables*: age; sex; 10 principal components (PCs); C-reactive Protein (CRP) polygenic score (PGS); White Blood Cell Counts (WBCC) PGS; Insulin Growth Factor-1 (IGF-1) PGS; Anxiety PGS; Depression PGS; Schizophrenia PGS; Insomnia PGS; Pain PGS.

b All variables: age; sex; 10 PCs; CRP PGS; WBCC PGS; IGF-1 PGS; PGS; Anxiety PGS; Depression PGS; Schizophrenia PGS; Insomnia PGS; Pain PGS; education; wealth; smoking status; alcohol consumption; mobility.

c Additional variables: medication, physical activity; BMI.

d Additional variables: health (i.e., chronic lung disease; coronary heart disease; abnormal heart rhythm; heart murmur; congestive heart failure; angina; hypertension; diabetes; cancer; Parkinson's; Alzheimer's; dementia; asthma; arthritis; osteoporosis; psychiatric disorder).

Table S9ai. **Longitudinal associations between insulin growth factor-1 and hospitalisation for diseases of the genitourinary system (*n*=3,524)**

**Adjustments**	**IGF-1**				
	**HR**	**SE**	**95% CI**		***p***
**Genitourinary Disorders**					
Model 1: *Unadjusted*	0.71	0.07	0.58	0.86	0.001
Model 2: *Model 1 + demographics & genetics* [a]	1.03	0.11	0.84	1.26	0.789
Model 3: *Model 2 + Fully Adjusted* [b]	1.04	0.11	0.85	1.27	0.732
Model 2a: *Demographics*	1.03	0.11	0.84	1.27	0.760
Model 2b: *Genetics*	0.70	0.07	0.58	0.86	0.001
Model 3a: Model 3 + *Medication*	1.04	0.11	0.85	1.27	0.734
Model 3b: Model 3 + *Physical Activity*	1.05	0.11	0.86	1.29	0.617
Model 3c: Model 3 + *Body Mass Index*	1.05	0.11	0.85	1.28	0.668
Model 3d: Model 3 + *Fully Adjusted* [c]	1.06	0.11	0.87	1.30	0.558
Model 4a: Model 2 + *Health*	1.03	0.11	0.84	1.26	0.789
Model 4b: Model 3 + *Health*	1.04	0.11	0.85	1.27	0.733
Model 4c: Model 3 + *Fully Adjusted* [d]	1.06	0.11	0.87	1.30	0.557

Notes: The *low-risk* group is the reference; HR = hazard ratio; SE = standard error; CI = confidence interval; *p* = significance value.

a *Demographic and genetic variables*: age; sex; 10 principal components (PCs); C-reactive Protein (CRP) polygenic score (PGS); White Blood Cell Counts (WBCC) PGS; Insulin Growth Factor-1 (IGF-1) PGS; Anxiety PGS; Depression PGS; Schizophrenia PGS; Insomnia PGS; Pain PGS.

b All variables: age; sex; 10 PCs; CRP PGS; WBCC PGS; IGF-1 PGS; PGS; Anxiety PGS; Depression PGS; Schizophrenia PGS; Insomnia PGS; Pain PGS; education; wealth; smoking status; alcohol consumption; mobility.

c Additional variables: medication, physical activity; BMI.

d Additional variables: health (i.e., chronic lung disease; coronary heart disease; abnormal heart rhythm; heart murmur; congestive heart failure; angina; hypertension; diabetes; cancer; Parkinson's; Alzheimer's; dementia; asthma; arthritis; osteoporosis; psychiatric disorder).

Table S9aj. **Longitudinal associations between immune and neuroendocrine profiles and hospitalisation for infectious and parasitic diseases (*n*=4,118)**

Adjustments	Immune and Neuroendocrine Profiles				
	HR	SE	95% CI		*p*
***Moderate-risk* Profile | Infections**					
Model 1: *Unadjusted*	1.29	0.12	1.07	1.54	0.007
Model 2: *Model 1 + demographics & genetics* [a]	1.11	0.10	0.92	1.33	0.287
Model 3: *Model 2 + Fully Adjusted* [b]	1.02	0.10	0.85	1.23	0.810
Model 2a: *Demographics*	1.12	0.11	0.93	1.35	0.225
Model 2b: *Genetics*	1.27	0.12	1.06	1.53	0.011
Model 3a: Model 3 + *Medication*	1.02	0.10	0.85	1.23	0.824
Model 3b: Model 3 + *Physical Activity*	1.00	0.10	0.83	1.21	0.978
Model 3c: Model 3 + *Body Mass Index*	1.04	0.10	0.85	1.26	0.717
Model 3d: Model 3 + *Fully Adjusted* [c]	1.02	0.10	0.84	1.24	0.842
Model 4a: Model 2 + *Health*	1.11	0.10	0.92	1.33	0.287
Model 4b: Model 3 + *Health*	1.02	0.10	0.84	1.23	0.853
Model 4c: Model 3 + *Fully Adjusted* [d]	1.02	0.10	0.84	1.23	0.877
***High-risk* Profile | Infections**					
Model 1: *Unadjusted*	1.78	0.22	1.39	2.27	<0.001
Model 2: *Model 1 + demographics & genetics* [a]	1.55	0.20	1.21	1.98	0.001
Model 3: *Model 2 + Fully Adjusted* [b]	1.38	0.18	1.08	1.78	0.011
Model 2a: *Demographics*	1.57	0.20	1.23	2.01	<0.001
Model 2b: *Genetics*	1.77	0.22	1.39	2.26	<0.001
Model 3a: Model 3 + *Medication*	1.39	0.18	1.08	1.78	0.011
Model 3b: Model 3 + *Physical Activity*	1.31	0.17	1.02	1.69	0.034
Model 3c: Model 3 + *Body Mass Index*	1.40	0.18	1.09	1.81	0.010
Model 3d: Model 3 + *Fully Adjusted* [c]	1.34	0.18	1.04	1.74	0.025
Model 4a: Model 2 + *Health*	1.55	0.20	1.21	1.98	0.001
Model 4b: Model 3 + *Health*	1.38	0.18	1.07	1.77	0.012
Model 4c: Model 3 + *Fully Adjusted* [d]	1.34	0.18	1.04	1.74	0.025

Notes: The *low-risk* group is the reference; HR = hazard ratio; SE = standard error; CI = confidence interval; *p* = significance value.

a *Demographic and genetic variables*: age; sex; 10 principal components (PCs); C-reactive Protein (CRP) polygenic score (PGS); White Blood Cell Counts (WBCC) PGS; Insulin Growth Factor-1 (IGF-1) PGS; Anxiety PGS; Depression PGS; Schizophrenia PGS; Insomnia PGS; Pain PGS.

b All variables: age; sex; 10 PCs; CRP PGS; WBCC PGS; IGF-1 PGS; PGS; Anxiety PGS; Depression PGS; Schizophrenia PGS; Insomnia PGS; Pain PGS; education; wealth; smoking status; alcohol consumption; mobility.

c Additional variables: medication, physical activity; BMI.

d Additional variables: health (i.e., chronic lung disease; coronary heart disease; abnormal heart rhythm; heart murmur; congestive heart failure; angina; hypertension; diabetes; cancer; Parkinson's; Alzheimer's; dementia; asthma; arthritis; osteoporosis; psychiatric disorder).

Table S9ak. **Longitudinal associations between C-reactive protein and hospitalisation for infectious and parasitic diseases (*n*=4,118)**

**Adjustments**	**CRP**				
	**HR**	**SE**	**95% CI**		***p***
**Infections**					
Model 1: *Unadjusted*	1.31	0.08	1.17	1.48	<0.001
Model 2: *Model 1 + demographics & genetics* [a]	1.20	0.08	1.06	1.36	0.004
Model 3: *Model 2 + Fully Adjusted* [b]	1.12	0.07	0.99	1.27	0.076
Model 2a: *Demographics*	1.21	0.08	1.07	1.37	0.002
Model 2b: *Genetics*	1.31	0.08	1.16	1.47	<0.001
Model 3a: Model 3 + *Medication*	1.12	0.07	0.99	1.28	0.074
Model 3b: Model 3 + *Physical Activity*	1.09	0.07	0.96	1.24	0.184
Model 3c: Model 3 + *Body Mass Index*	1.14	0.08	1.00	1.30	0.059
Model 3d: Model 3 + *Fully Adjusted* [c]	1.11	0.08	0.97	1.27	0.127
Model 4a: Model 2 + *Health*	1.20	0.08	1.06	1.36	0.004
Model 4b: Model 3 + *Health*	1.12	0.07	0.98	1.27	0.087
Model 4c: Model 3 + *Fully Adjusted* [d]	1.11	0.08	0.97	1.26	0.140

Notes: The *low-risk* group is the reference; HR = hazard ratio; SE = standard error; CI = confidence interval; *p* = significance value.

a *Demographic and genetic variables*: age; sex; 10 principal components (PCs); C-reactive Protein (CRP) polygenic score (PGS); White Blood Cell Counts (WBCC) PGS; Insulin Growth Factor-1 (IGF-1) PGS; Anxiety PGS; Depression PGS; Schizophrenia PGS; Insomnia PGS; Pain PGS.

b All variables: age; sex; 10 PCs; CRP PGS; WBCC PGS; IGF-1 PGS; PGS; Anxiety PGS; Depression PGS; Schizophrenia PGS; Insomnia PGS; Pain PGS; education; wealth; smoking status; alcohol consumption; mobility.

c Additional variables: medication, physical activity; BMI.

d Additional variables: health (i.e., chronic lung disease; coronary heart disease; abnormal heart rhythm; heart murmur; congestive heart failure; angina; hypertension; diabetes; cancer; Parkinson's; Alzheimer's; dementia; asthma; arthritis; osteoporosis; psychiatric disorder).

Table S9al. **Longitudinal associations between fibrinogen and hospitalisation for infectious and parasitic diseases (*n*=4,118)**

**Adjustments**	**Fb**				
	**HR**	**SE**	**95% CI**		***p***
**Infections**					
Model 1: *Unadjusted*	1.34	0.10	1.16	1.55	<0.001
Model 2: *Model 1 + demographics & genetics* [a]	1.22	0.10	1.05	1.42	0.011
Model 3: *Model 2 + Fully Adjusted* [b]	1.13	0.09	0.97	1.32	0.111
Model 2a: *Demographics*	1.23	0.10	1.06	1.43	0.008
Model 2b: *Genetics*	1.34	0.10	1.15	1.55	<0.001
Model 3a: Model 3 + *Medication*	1.14	0.09	0.97	1.33	0.110
Model 3b: Model 3 + *Physical Activity*	1.11	0.09	0.96	1.30	0.170
Model 3c: Model 3 + *Body Mass Index*	1.14	0.09	0.97	1.33	0.104
Model 3d: Model 3 + *Fully Adjusted* [c]	1.12	0.09	0.96	1.31	0.149
Model 4a: Model 2 + *Health*	1.21	0.09	1.04	1.41	0.013
Model 4b: Model 3 + *Health*	1.14	0.09	0.97	1.33	0.107
Model 4c: Model 3 + *Fully Adjusted* [d]	1.12	0.09	0.96	1.31	0.144

Notes: The *low-risk* group is the reference; HR = hazard ratio; SE = standard error; CI = confidence interval; *p* = significance value.

a *Demographic and genetic variables*: age; sex; 10 principal components (PCs); C-reactive Protein (CRP) polygenic score (PGS); White Blood Cell Counts (WBCC) PGS; Insulin Growth Factor-1 (IGF-1) PGS; Anxiety PGS; Depression PGS; Schizophrenia PGS; Insomnia PGS; Pain PGS.

b All variables: age; sex; 10 PCs; CRP PGS; WBCC PGS; IGF-1 PGS; PGS; Anxiety PGS; Depression PGS; Schizophrenia PGS; Insomnia PGS; Pain PGS; education; wealth; smoking status; alcohol consumption; mobility.

c Additional variables: medication, physical activity; BMI.

d Additional variables: health (i.e., chronic lung disease; coronary heart disease; abnormal heart rhythm; heart murmur; congestive heart failure; angina; hypertension; diabetes; cancer; Parkinson's; Alzheimer's; dementia; asthma; arthritis; osteoporosis; psychiatric disorder).

Table S9am. **Longitudinal associations between white blood cell counts and hospitalisation for infectious and parasitic diseases (*n*=4,118)**

Adjustments	WBCC				
	HR	SE	95% CI		*p*
**Infections**					
Model 1: *Unadjusted*	1.77	0.31	1.26	2.48	0.001
Model 2: *Model 1 + demographics & genetics* [a]	1.63	0.29	1.14	2.31	0.007
Model 3: *Model 2 + Fully Adjusted* [b]	1.37	0.26	0.95	1.97	0.093
Model 2a: *Demographics*	1.58	0.28	1.12	2.23	0.010
Model 2b: *Genetics*	1.78	0.31	1.27	2.51	0.001
Model 3a: Model 3 + *Medication*	1.37	0.26	0.95	1.98	0.092
Model 3b: Model 3 + *Physical Activity*	1.33	0.25	0.92	1.91	0.127
Model 3c: Model 3 + *Body Mass Index*	1.38	0.26	0.95	2.00	0.087
Model 3d: Model 3 + *Fully Adjusted* [c]	1.35	0.25	0.94	1.96	0.108
Model 4a: Model 2 + *Health*	1.59	0.29	1.12	2.26	0.010
Model 4b: Model 3 + *Health*	1.36	0.25	0.94	1.96	0.102
Model 4c: Model 3 + *Fully Adjusted* [d]	1.35	0.25	0.93	1.95	0.114

Notes: The *low-risk* group is the reference; HR = hazard ratio; SE = standard error; CI = confidence interval; *p* = significance value.

a *Demographic and genetic variables*: age; sex; 10 principal components (PCs); C-reactive Protein (CRP) polygenic score (PGS); White Blood Cell Counts (WBCC) PGS; Insulin Growth Factor-1 (IGF-1) PGS; Anxiety PGS; Depression PGS; Schizophrenia PGS; Insomnia PGS; Pain PGS.

b All variables: age; sex; 10 PCs; CRP PGS; WBCC PGS; IGF-1 PGS; PGS; Anxiety PGS; Depression PGS; Schizophrenia PGS; Insomnia PGS; Pain PGS; education; wealth; smoking status; alcohol consumption; mobility.

c Additional variables: medication, physical activity; BMI.

d Additional variables: health (i.e., chronic lung disease; coronary heart disease; abnormal heart rhythm; heart murmur; congestive heart failure; angina; hypertension; diabetes; cancer; Parkinson's; Alzheimer's; dementia; asthma; arthritis; osteoporosis; psychiatric disorder).

Table S9an. **Longitudinal associations between insulin growth factor-1 and hospitalisation for infectious and parasitic diseases (*n*=4,118)**

Adjustments	IGF-1				
	**HR**	**SE**	**95% CI**		***p***
**Infections**					
Model 1: *Unadjusted*	0.70	0.09	0.54	0.90	0.005
Model 2: *Model 1 + demographics & genetics* [a]	1.02	0.14	0.78	1.32	0.914
Model 3: *Model 2 + Fully Adjusted* [b]	1.04	0.14	0.80	1.35	0.761
Model 2a: *Demographics*	0.99	0.13	0.77	1.29	0.963
Model 2b: *Genetics*	0.70	0.09	0.55	0.91	0.007
Model 3a: Model 3 + *Medication*	1.04	0.14	0.80	1.35	0.763
Model 3b: Model 3 + *Physical Activity*	1.05	0.14	0.82	1.36	0.688
Model 3c: Model 3 + *Body Mass Index*	1.04	0.14	0.80	1.35	0.763
Model 3d: Model 3 + *Fully Adjusted* [c]	1.05	0.14	0.81	1.36	0.699
Model 4a: Model 2 + *Health*	1.02	0.14	0.78	1.32	0.891
Model 4b: Model 3 + *Health*	1.04	0.14	0.81	1.35	0.747
Model 4c: Model 3 + *Fully Adjusted* [d]	1.06	0.14	0.82	1.36	0.683

Notes: The *low-risk* group is the reference; HR = hazard ratio; SE = standard error; CI = confidence interval; *p* = significance value.

a *Demographic and genetic variables*: age; sex; 10 principal components (PCs); C-reactive Protein (CRP) polygenic score (PGS); White Blood Cell Counts (WBCC) PGS; Insulin Growth Factor-1 (IGF-1) PGS; Anxiety PGS; Depression PGS; Schizophrenia PGS; Insomnia PGS; Pain PGS.

b All variables: age; sex; 10 PCs; CRP PGS; WBCC PGS; IGF-1 PGS; PGS; Anxiety PGS; Depression PGS; Schizophrenia PGS; Insomnia PGS; Pain PGS; education; wealth; smoking status; alcohol consumption; mobility.

c Additional variables: medication, physical activity; BMI.

d Additional variables: health (i.e., chronic lung disease; coronary heart disease; abnormal heart rhythm; heart murmur; congestive heart failure; angina; hypertension; diabetes; cancer; Parkinson's; Alzheimer's; dementia; asthma; arthritis; osteoporosis; psychiatric disorder).

Table S9ao. **Longitudinal associations between immune and neuroendocrine profiles and hospitalisation for diseases of the musculoskeletal system and connective tissue (*n*=3,524)**

Adjustments	Immune and Neuroendocrine Profiles				
	**HR**	**SE**	**95% CI**		***p***
***Moderate-risk* Profile | Musculoskeletal Disorders**					
Model 1: *Unadjusted*	1.36	0.09	1.20	1.55	<0.001
Model 2: *Model 1 + demographics & genetics* [a]	1.22	0.08	1.07	1.39	0.003
Model 3: *Model 2 + Fully Adjusted* [b]	1.13	0.08	0.99	1.30	0.063
Model 2a: *Demographics*	1.20	0.08	1.05	1.36	0.007
Model 2b: *Genetics*	1.36	0.09	1.20	1.55	<0.001
Model 3a: Model 3 + *Medication*	1.14	0.08	0.99	1.30	0.062
Model 3b: Model 3 + *Physical Activity*	1.12	0.08	0.98	1.28	0.087
Model 3c: Model 3 + *Body Mass Index*	1.11	0.08	0.97	1.27	0.136
Model 3d: Model 3 + *Fully Adjusted* [c]	1.10	0.08	0.96	1.26	0.167
Model 4a: Model 2 + *Health*	1.19	0.08	1.05	1.36	0.008
Model 4b: Model 3 + *Health*	1.12	0.08	0.98	1.28	0.092
Model 4c: Model 3 + *Fully Adjusted* [d]	1.09	0.08	0.95	1.26	0.201
***High-risk* Profile | Musculoskeletal Disorders**					
Model 1: *Unadjusted*	1.49	0.14	1.24	1.80	<0.001
Model 2: *Model 1 + demographics & genetics* [a]	1.41	0.14	1.17	1.70	<0.001
Model 3: *Model 2 + Fully Adjusted* [b]	1.28	0.13	1.06	1.55	0.012
Model 2a: *Demographics*	1.39	0.13	1.15	1.68	0.001
Model 2b: *Genetics*	1.57	0.15	1.30	1.89	<0.001
Model 3a: Model 3 + *Medication*	1.28	0.13	1.06	1.55	0.012
Model 3b: Model 3 + *Physical Activity*	1.25	0.12	1.03	1.51	0.025
Model 3c: Model 3 + *Body Mass Index*	1.25	0.13	1.03	1.52	0.027
Model 3d: Model 3 + *Fully Adjusted* [c]	1.22	0.12	1.00	1.48	0.052
Model 4a: Model 2 + *Health*	1.37	0.13	1.13	1.65	0.001
Model 4b: Model 3 + *Health*	1.26	0.12	1.04	1.52	0.020
Model 4c: Model 3 + *Fully Adjusted* [d]	1.20	0.12	0.99	1.46	0.069

Notes: The *low-risk* group is the reference; HR = hazard ratio; SE = standard error; CI = confidence interval; *p* = significance value.

a *Demographic and genetic variables*: age; sex; 10 principal components (PCs); C-reactive Protein (CRP) polygenic score (PGS); White Blood Cell Counts (WBCC) PGS; Insulin Growth Factor-1 (IGF-1) PGS; Anxiety PGS; Depression PGS; Schizophrenia PGS; Insomnia PGS; Pain PGS.

b All variables: age; sex; 10 PCs; CRP PGS; WBCC PGS; IGF-1 PGS; PGS; Anxiety PGS; Depression PGS; Schizophrenia PGS; Insomnia PGS; Pain PGS; education; wealth; smoking status; alcohol consumption; mobility.

c Additional variables: medication, physical activity; BMI.

d Additional variables: health (i.e., chronic lung disease; coronary heart disease; abnormal heart rhythm; heart murmur; congestive heart failure; angina; hypertension; diabetes; cancer; Parkinson's; Alzheimer's; dementia; asthma; arthritis; osteoporosis; psychiatric disorder).

Table S9ap. **Longitudinal associations between C-reactive protein and hospitalisation for diseases of the musculoskeletal system and connective tissue (*n*=3,524)**

**Adjustments**	**CRP**				
	**HR**	**SE**	**95% CI**		***p***
**Musculoskeletal Disorders**					
Model 1: *Unadjusted*	1.23	0.06	1.13	1.35	<0.001
Model 2: *Model 1 + demographics & genetics* [a]	1.26	0.06	1.15	1.37	<0.001
Model 3: *Model 2 + Fully Adjusted* [b]	1.18	0.06	1.08	1.29	<0.001
Model 2a: *Demographics*	1.24	0.06	1.13	1.35	<0.001
Model 2b: *Genetics*	1.34	0.06	1.23	1.47	<0.001
Model 3a: Model 3 + *Medication*	1.18	0.06	1.08	1.29	<0.001
Model 3b: Model 3 + *Physical Activity*	1.17	0.05	1.06	1.28	0.001
Model 3c: Model 3 + *Body Mass Index*	1.17	0.06	1.06	1.28	0.002
Model 3d: Model 3 + *Fully Adjusted* [c]	1.15	0.06	1.05	1.27	0.004
Model 4a: Model 2 + *Health*	1.23	0.06	1.13	1.35	<0.001
Model 4b: Model 3 + *Health*	1.17	0.06	1.07	1.28	0.001
Model 4c: Model 3 + *Fully Adjusted* [d]	1.14	0.06	1.04	1.26	0.007

Notes: The *low-risk* group is the reference; HR = hazard ratio; SE = standard error; CI = confidence interval; *p* = significance value.

a *Demographic and genetic variables*: age; sex; 10 principal components (PCs); C-reactive Protein (CRP) polygenic score (PGS); White Blood Cell Counts (WBCC) PGS; Insulin Growth Factor-1 (IGF-1) PGS; Anxiety PGS; Depression PGS; Schizophrenia PGS; Insomnia PGS; Pain PGS.

b All variables: age; sex; 10 PCs; CRP PGS; WBCC PGS; IGF-1 PGS; PGS; Anxiety PGS; Depression PGS; Schizophrenia PGS; Insomnia PGS; Pain PGS; education; wealth; smoking status; alcohol consumption; mobility.

c Additional variables: medication, physical activity; BMI.

d Additional variables: health (i.e., chronic lung disease; coronary heart disease; abnormal heart rhythm; heart murmur; congestive heart failure; angina; hypertension; diabetes; cancer; Parkinson's; Alzheimer's; dementia; asthma; arthritis; osteoporosis; psychiatric disorder).

Table S9aq. **Longitudinal associations between fibrinogen and hospitalisation for diseases of the musculoskeletal system and connective tissue (*n*=3,524)**

Adjustments	Fb				
	HR	SE	95% CI		*p*
**Musculoskeletal Disorders**					
Model 1: *Unadjusted*	1.24	0.07	1.12	1.38	<0.001
Model 2: *Model 1 + demographics & genetics* [a]	1.12	0.06	1.01	1.26	0.040
Model 3: *Model 2 + Fully Adjusted* [b]	1.07	0.06	0.95	1.19	0.273
Model 2a: *Demographics*	1.12	0.06	1.00	1.25	0.042
Model 2b: *Genetics*	1.23	0.07	1.10	1.37	<0.001
Model 3a: Model 3 + *Medication*	1.07	0.06	0.95	1.19	0.273
Model 3b: Model 3 + *Physical Activity*	1.06	0.06	0.95	1.18	0.335
Model 3c: Model 3 + *Body Mass Index*	1.05	0.06	0.94	1.18	0.415
Model 3d: Model 3 + *Fully Adjusted* [c]	1.04	0.06	0.93	1.17	0.479
Model 4a: Model 2 + *Health*	1.11	0.06	0.99	1.24	0.070
Model 4b: Model 3 + *Health*	1.06	0.06	0.95	1.19	0.308
Model 4c: Model 3 + *Fully Adjusted* [d]	1.04	0.06	0.93	1.16	0.505

Notes: The *low-risk* group is the reference; HR = hazard ratio; SE = standard error; CI = confidence interval; *p* = significance value.

a *Demographic and genetic variables*: age; sex; 10 principal components (PCs); C-reactive Protein (CRP) polygenic score (PGS); White Blood Cell Counts (WBCC) PGS; Insulin Growth Factor-1 (IGF-1) PGS; Anxiety PGS; Depression PGS; Schizophrenia PGS; Insomnia PGS; Pain PGS.

b All variables: age; sex; 10 PCs; CRP PGS; WBCC PGS; IGF-1 PGS; PGS; Anxiety PGS; Depression PGS; Schizophrenia PGS; Insomnia PGS; Pain PGS; education; wealth; smoking status; alcohol consumption; mobility.

c Additional variables: medication, physical activity; BMI.

d Additional variables: health (i.e., chronic lung disease; coronary heart disease; abnormal heart rhythm; heart murmur; congestive heart failure; angina; hypertension; diabetes; cancer; Parkinson's; Alzheimer's; dementia; asthma; arthritis; osteoporosis; psychiatric disorder).

Table S9ar. **Longitudinal associations between white blood cell counts and hospitalisation for diseases of the musculoskeletal system and connective tissue (*n*=3,524)**

Adjustments	WBCC				
	**HR**	**SE**	**95% CI**		***p***
**Musculoskeletal Disorders**					
Model 1: *Unadjusted*	1.29	0.16	1.01	1.64	0.039
Model 2: *Model 1 + demographics & genetics* [a]	1.29	0.16	1.01	1.66	0.042
Model 3: *Model 2 + Fully Adjusted* [b]	1.19	0.16	0.92	1.54	0.197
Model 2a: *Demographics*	1.26	0.16	0.99	1.61	0.066
Model 2b: *Genetics*	1.28	0.16	1.00	1.63	0.048
Model 3a: Model 3 + *Medication*	1.19	0.16	0.92	1.54	0.196
Model 3b: Model 3 + *Physical Activity*	1.16	0.15	0.89	1.50	0.266
Model 3c: Model 3 + *Body Mass Index*	1.15	0.15	0.88	1.49	0.308
Model 3d: Model 3 + *Fully Adjusted* [c]	1.12	0.15	0.86	1.46	0.384
Model 4a: Model 2 + *Health*	1.25	0.16	0.98	1.60	0.076
Model 4b: Model 3 + *Health*	1.16	0.15	0.89	1.50	0.271
Model 4c: Model 3 + *Fully Adjusted* [d]	1.11	0.15	0.85	1.44	0.453

Notes: The *low-risk* group is the reference; HR = hazard ratio; SE = standard error; CI = confidence interval; *p* = significance value.

a *Demographic and genetic variables*: age; sex; 10 principal components (PCs); C-reactive Protein (CRP) polygenic score (PGS); White Blood Cell Counts (WBCC) PGS; Insulin Growth Factor-1 (IGF-1) PGS; Anxiety PGS; Depression PGS; Schizophrenia PGS; Insomnia PGS; Pain PGS.

b All variables: age; sex; 10 PCs; CRP PGS; WBCC PGS; IGF-1 PGS; PGS; Anxiety PGS; Depression PGS; Schizophrenia PGS; Insomnia PGS; Pain PGS; education; wealth; smoking status; alcohol consumption; mobility.

c Additional variables: medication, physical activity; BMI.

d Additional variables: health (i.e., chronic lung disease; coronary heart disease; abnormal heart rhythm; heart murmur; congestive heart failure; angina; hypertension; diabetes; cancer; Parkinson's; Alzheimer's; dementia; asthma; arthritis; osteoporosis; psychiatric disorder).

Table S9as. **Longitudinal associations between insulin growth factor-1 and hospitalisation for diseases of the musculoskeletal system and connective tissue (*n*=3,524)**

Adjustments	IGF-1				
	HR	SE	95% CI		*p*
**Musculoskeletal Disorders**					
Model 1: *Unadjusted*	0.69	0.06	0.57	0.82	<0.001
Model 2: *Model 1 + demographics & genetics* [a]	0.94	0.09	0.78	1.14	0.554
Model 3: *Model 2 + Fully Adjusted* [b]	0.95	0.09	0.79	1.15	0.617
Model 2a: *Demographics*	0.95	0.09	0.79	1.14	0.570
Model 2b: *Genetics*	0.69	0.07	0.57	0.83	<0.001
Model 3a: Model 3 + *Medication*	0.95	0.09	0.79	1.15	0.617
Model 3b: Model 3 + *Physical Activity*	0.95	0.09	0.79	1.15	0.609
Model 3c: Model 3 + *Body Mass Index*	0.96	0.09	0.79	1.15	0.633
Model 3d: Model 3 + *Fully Adjusted* [c]	0.96	0.09	0.79	1.15	0.633
Model 4a: Model 2 + *Health*	0.95	0.09	0.79	1.15	0.582
Model 4b: Model 3 + *Health*	0.95	0.09	0.79	1.15	0.623
Model 4c: Model 3 + *Fully Adjusted* [d]	0.96	0.09	0.79	1.15	0.632

Notes: The *low-risk* group is the reference; HR = hazard ratio; SE = standard error; CI = confidence interval; *p* = significance value.

a *Demographic and genetic variables*: age; sex; 10 principal components (PCs); C-reactive Protein (CRP) polygenic score (PGS); White Blood Cell Counts (WBCC) PGS; Insulin Growth Factor-1 (IGF-1) PGS; Anxiety PGS; Depression PGS; Schizophrenia PGS; Insomnia PGS; Pain PGS.

b All variables: age; sex; 10 PCs; CRP PGS; WBCC PGS; IGF-1 PGS; PGS; Anxiety PGS; Depression PGS; Schizophrenia PGS; Insomnia PGS; Pain PGS; education; wealth; smoking status; alcohol consumption; mobility.

c Additional variables: medication, physical activity; BMI.

d Additional variables: health (i.e., chronic lung disease; coronary heart disease; abnormal heart rhythm; heart murmur; congestive heart failure; angina; hypertension; diabetes; cancer; Parkinson's; Alzheimer's; dementia; asthma; arthritis; osteoporosis; psychiatric disorder).

Table S9at. **Longitudinal associations between immune and neuroendocrine profiles and hospitalisation for diseases of the nervous system (*n*=4,057)**

**Adjustments**	**Immune and Neuroendocrine Profiles**				
	**HR**	**SE**	**95% CI**		***p***
Moderate-risk **Profile | Nervous Disorders**					
Model 1: *Unadjusted*	1.37	0.13	1.15	1.65	0.001
Model 2: *Model 1 + demographics & genetics* [a]	1.19	0.11	0.99	1.43	0.067
Model 3: *Model 2 + Fully Adjusted* [b]	1.12	0.11	0.93	1.35	0.220
Model 2a: *Demographics*	1.21	0.11	1.01	1.45	0.043
Model 2b: *Genetics*	1.36	0.13	1.13	1.62	0.001
Model 3a: Model 3 + *Medication*	1.12	0.11	0.93	1.35	0.220
Model 3b: Model 3 + *Physical Activity*	1.09	0.10	0.91	1.32	0.346
Model 3c: Model 3 + *Body Mass Index*	1.08	0.11	0.89	1.31	0.428
Model 3d: Model 3 + *Fully Adjusted* [c]	1.06	0.10	0.88	1.29	0.549
Model 4a: Model 2 + *Health*	1.19	0.11	0.99	1.43	0.067
Model 4b: Model 3 + *Health*	1.11	0.11	0.92	1.34	0.257
Model 4c: Model 3 + *Fully Adjusted* [d]	1.05	0.10	0.87	1.28	0.594
High-risk **Profile | Nervous Disorders**					
Model 1: *Unadjusted*	1.24	0.18	0.94	1.64	0.125
Model 2: *Model 1 + demographics & genetics* [a]	1.06	0.15	0.80	1.41	0.666
Model 3: *Model 2 + Fully Adjusted* [b]	0.97	0.14	0.73	1.28	0.808
Model 2a: *Demographics*	1.08	0.15	0.82	1.43	0.592
Model 2b: *Genetics*	1.22	0.17	0.92	1.61	0.165
Model 3a: Model 3 + *Medication*	0.97	0.14	0.73	1.29	0.830
Model 3b: Model 3 + *Physical Activity*	0.90	0.13	0.68	1.20	0.486
Model 3c: Model 3 + *Body Mass Index*	0.93	0.14	0.69	1.24	0.598
Model 3d: Model 3 + *Fully Adjusted* [c]	0.88	0.13	0.66	1.17	0.376
Model 4a: Model 2 + *Health*	1.06	0.15	0.80	1.41	0.666
Model 4b: Model 3 + *Health*	0.95	0.14	0.71	1.26	0.721
Model 4c: Model 3 + *Fully Adjusted* [d]	0.87	0.13	0.65	1.16	0.332

Notes: The *low-risk* group is the reference; HR = hazard ratio; SE = standard error; CI = confidence interval; *p* = significance value.

a *Demographic and genetic variables*: age; sex; 10 principal components (PCs); C-reactive Protein (CRP) polygenic score (PGS); White Blood Cell Counts (WBCC) PGS; Insulin Growth Factor-1 (IGF-1) PGS; Anxiety PGS; Depression PGS; Schizophrenia PGS; Insomnia PGS; Pain PGS.

b All variables: age; sex; 10 PCs; CRP PGS; WBCC PGS; IGF-1 PGS; PGS; Anxiety PGS; Depression PGS; Schizophrenia PGS; Insomnia PGS; Pain PGS; education; wealth; smoking status; alcohol consumption; mobility.

c Additional variables: medication, physical activity; BMI.

d Additional variables: health (i.e., chronic lung disease; coronary heart disease; abnormal heart rhythm; heart murmur; congestive heart failure; angina; hypertension; diabetes; cancer; Parkinson's; Alzheimer's; dementia; asthma; arthritis; osteoporosis; psychiatric disorder).

Table S9au. **Longitudinal associations between C-reactive protein and hospitalisation for diseases of the nervous system (*n*=4,057)**

**Adjustments**	**CRP**				
	**HR**	**SE**	**95% CI**		***p***
**Nervous Disorders**					
Model 1: *Unadjusted*	1.20	0.08	1.06	1.36	0.004
Model 2: *Model 1 + demographics & genetics* [a]	1.09	0.07	0.95	1.23	0.213
Model 3: *Model 2 + Fully Adjusted* [b]	1.03	0.07	0.91	1.18	0.630
Model 2a: *Demographics*	1.10	0.07	0.97	1.25	0.150
Model 2b: *Genetics*	1.19	0.08	1.05	1.34	0.007
Model 3a: Model 3 + *Medication*	1.03	0.07	0.91	1.18	0.610
Model 3b: Model 3 + *Physical Activity*	1.00	0.07	0.88	1.14	0.976
Model 3c: Model 3 + *Body Mass Index*	1.00	0.07	0.87	1.15	0.996
Model 3d: Model 3 + *Fully Adjusted* [c]	0.97	0.07	0.85	1.12	0.703
Model 4a: Model 2 + *Health*	1.09	0.07	0.95	1.23	0.213
Model 4b: Model 3 + *Health*	1.02	0.07	0.90	1.16	0.752
Model 4c: Model 3 + *Fully Adjusted* [d]	0.97	0.07	0.84	1.11	0.609

Notes: The *low-risk* group is the reference; HR = hazard ratio; SE = standard error; CI = confidence interval; *p* = significance value.

a *Demographic and genetic variables*: age; sex; 10 principal components (PCs); C-reactive Protein (CRP) polygenic score (PGS); White Blood Cell Counts (WBCC) PGS; Insulin Growth Factor-1 (IGF-1) PGS; Anxiety PGS; Depression PGS; Schizophrenia PGS; Insomnia PGS; Pain PGS.

b All variables: age; sex; 10 PCs; CRP PGS; WBCC PGS; IGF-1 PGS; PGS; Anxiety PGS; Depression PGS; Schizophrenia PGS; Insomnia PGS; Pain PGS; education; wealth; smoking status; alcohol consumption; mobility.

c Additional variables: medication, physical activity; BMI.

d Additional variables: health (i.e., chronic lung disease; coronary heart disease; abnormal heart rhythm; heart murmur; congestive heart failure; angina; hypertension; diabetes; cancer; Parkinson's; Alzheimer's; dementia; asthma; arthritis; osteoporosis; psychiatric disorder).

Table S9av. **Longitudinal associations between fibrinogen and hospitalisation for diseases of the nervous system (*n*=4,057)**

Adjustments	Fb				
	HR	SE	95% CI		*p*
**Nervous Disorders**					
Model 1: *Unadjusted*	1.20	0.09	1.03	1.39	0.018
Model 2: *Model 1 + demographics & genetics* [a]	1.07	0.09	0.92	1.26	0.377
Model 3: *Model 2 + Fully Adjusted* [b]	0.99	0.08	0.84	1.16	0.866
Model 2a: *Demographics*	1.08	0.09	0.92	1.26	0.346
Model 2b: *Genetics*	1.18	0.09	1.02	1.38	0.029
Model 3a: Model 3 + *Medication*	0.99	0.08	0.84	1.16	0.880
Model 3b: Model 3 + *Physical Activity*	0.97	0.08	0.82	1.13	0.676
Model 3c: Model 3 + *Body Mass Index*	0.97	0.08	0.82	1.14	0.678
Model 3d: Model 3 + *Fully Adjusted* [c]	0.95	0.08	0.81	1.12	0.560
Model 4a: Model 2 + *Health*	1.06	0.09	0.91	1.24	0.448
Model 4b: Model 3 + *Health*	0.98	0.08	0.84	1.15	0.828
Model 4c: Model 3 + *Fully Adjusted* [d]	0.95	0.08	0.81	1.12	0.534

Notes: The *low-risk* group is the reference; HR = hazard ratio; SE = standard error; CI = confidence interval; *p* = significance value.

a *Demographic and genetic variables*: age; sex; 10 principal components (PCs); C-reactive Protein (CRP) polygenic score (PGS); White Blood Cell Counts (WBCC) PGS; Insulin Growth Factor-1 (IGF-1) PGS; Anxiety PGS; Depression PGS; Schizophrenia PGS; Insomnia PGS; Pain PGS.

b All variables: age; sex; 10 PCs; CRP PGS; WBCC PGS; IGF-1 PGS; PGS; Anxiety PGS; Depression PGS; Schizophrenia PGS; Insomnia PGS; Pain PGS; education; wealth; smoking status; alcohol consumption; mobility.

c Additional variables: medication, physical activity; BMI.

d Additional variables: health (i.e., chronic lung disease; coronary heart disease; abnormal heart rhythm; heart murmur; congestive heart failure; angina; hypertension; diabetes; cancer; Parkinson's; Alzheimer's; dementia; asthma; arthritis; osteoporosis; psychiatric disorder).

Table S9aw. **Longitudinal associations between white blood cell counts and hospitalisation for diseases of the nervous system (*n*=4,057)**

**Adjustments**	**WBCC**				
	**HR**	**SE**	**95% CI**		***p***
**Nervous Disorders**					
Model 1: *Unadjusted*	1.49	0.26	1.06	2.10	0.023
Model 2: *Model 1 + demographics & genetics* [a]	1.34	0.24	0.94	1.91	0.109
Model 3: *Model 2 + Fully Adjusted* [b]	1.08	0.20	0.74	1.56	0.703
Model 2a: *Demographics*	1.26	0.23	0.89	1.80	0.195
Model 2b: *Genetics*	1.52	0.27	1.08	2.15	0.018
Model 3a: Model 3 + *Medication*	1.08	0.21	0.74	1.56	0.692
Model 3b: Model 3 + *Physical Activity*	1.03	0.20	0.71	1.49	0.896
Model 3c: Model 3 + *Body Mass Index*	1.03	0.20	0.71	1.50	0.873
Model 3d: Model 3 + *Fully Adjusted* [c]	0.99	0.19	0.68	1.45	0.975
Model 4a: Model 2 + *Health*	1.30	0.24	0.91	1.87	0.147
Model 4b: Model 3 + *Health*	1.06	0.20	0.73	1.53	0.778
Model 4c: Model 3 + *Fully Adjusted* [d]	0.98	0.19	0.67	1.43	0.917

Notes: The *low-risk* group is the reference; HR = hazard ratio; SE = standard error; CI = confidence interval; *p* = significance value.

a *Demographic and genetic variables*: age; sex; 10 principal components (PCs); C-reactive Protein (CRP) polygenic score (PGS); White Blood Cell Counts (WBCC) PGS; Insulin Growth Factor-1 (IGF-1) PGS; Anxiety PGS; Depression PGS; Schizophrenia PGS; Insomnia PGS; Pain PGS.

b All variables: age; sex; 10 PCs; CRP PGS; WBCC PGS; IGF-1 PGS; PGS; Anxiety PGS; Depression PGS; Schizophrenia PGS; Insomnia PGS; Pain PGS; education; wealth; smoking status; alcohol consumption; mobility.

c Additional variables: medication, physical activity; BMI.

d Additional variables: health (i.e., chronic lung disease; coronary heart disease; abnormal heart rhythm; heart murmur; congestive heart failure; angina; hypertension; diabetes; cancer; Parkinson's; Alzheimer's; dementia; asthma; arthritis; osteoporosis; psychiatric disorder).

Table S9ax. **Longitudinal associations between insulin growth factor-1 and hospitalisation for diseases of the nervous system (*n*=4,057)**

Adjustments	IGF-1				
	HR	SE	95% CI		*p*
**Nervous Disorders**					
Model 1: *Unadjusted*	0.64	0.08	0.49	0.82	0.001
Model 2: *Model 1 + demographics & genetics* [a]	0.84	0.11	0.64	1.09	0.185
Model 3: *Model 2 + Fully Adjusted* [b]	0.84	0.11	0.64	1.09	0.178
Model 2a: *Demographics*	0.84	0.11	0.65	1.10	0.201
Model 2b: *Genetics*	0.64	0.08	0.50	0.83	0.001
Model 3a: Model 3 + *Medication*	0.83	0.11	0.64	1.08	0.172
Model 3b: Model 3 + *Physical Activity*	0.85	0.11	0.65	1.10	0.205
Model 3c: Model 3 + *Body Mass Index*	0.84	0.11	0.65	1.09	0.188
Model 3d: Model 3 + *Fully Adjusted* [c]	0.85	0.11	0.65	1.10	0.208
Model 4a: Model 2 + *Health*	0.85	0.11	0.65	1.10	0.208
Model 4b: Model 3 + *Health*	0.84	0.11	0.65	1.09	0.194
Model 4c: Model 3 + *Fully Adjusted* [d]	0.85	0.11	0.66	1.10	0.224

Notes: The *low-risk* group is the reference; HR = hazard ratio; SE = standard error; CI = confidence interval; *p* = significance value.

a *Demographic and genetic variables*: age; sex; 10 principal components (PCs); C-reactive Protein (CRP) polygenic score (PGS); White Blood Cell Counts (WBCC) PGS; Insulin Growth Factor-1 (IGF-1) PGS; Anxiety PGS; Depression PGS; Schizophrenia PGS; Insomnia PGS; Pain PGS.

b All variables: age; sex; 10 PCs; CRP PGS; WBCC PGS; IGF-1 PGS; PGS; Anxiety PGS; Depression PGS; Schizophrenia PGS; Insomnia PGS; Pain PGS; education; wealth; smoking status; alcohol consumption; mobility.

c Additional variables: medication, physical activity; BMI.

d Additional variables: health (i.e., chronic lung disease; coronary heart disease; abnormal heart rhythm; heart murmur; congestive heart failure; angina; hypertension; diabetes; cancer; Parkinson's; Alzheimer's; dementia; asthma; arthritis; osteoporosis; psychiatric disorder).

Table S9ay. **Longitudinal associations between immune and neuroendocrine profiles and hospitalisation for diseases of the respiratory system (*n*=3,846)**

**Adjustments**	**Immune and Neuroendocrine Profiles**				
	**HR**	**SE**	**95% CI**		***p***
***Moderate-risk* Profile | Respiratory Disorders**					
Model 1: *Unadjusted*	1.59	0.12	1.38	1.84	<0.001
Model 2: *Model 1 + demographics & genetics* [a]	1.41	0.11	1.22	1.63	<0.001
Model 3: *Model 2 + Fully Adjusted* [b]	1.26	0.10	1.09	1.47	0.002
Model 2a: *Demographics*	1.40	0.10	1.21	1.62	<0.001
Model 2b: *Genetics*	1.60	0.12	1.39	1.85	<0.001
Model 3a: Model 3 + *Medication*	1.26	0.10	1.08	1.46	0.003
Model 3b: Model 3 + *Physical Activity*	1.25	0.10	1.08	1.45	0.004
Model 3c: Model 3 + *Body Mass Index*	1.29	0.10	1.11	1.51	0.001
Model 3d: Model 3 + *Fully Adjusted* [c]	1.28	0.10	1.09	1.49	0.002
Model 4a: Model 2 + *Health*	1.41	0.11	1.22	1.63	<0.001
Model 4b: Model 3 + *Health*	1.25	0.10	1.08	1.45	0.003
Model 4c: Model 3 + *Fully Adjusted* [d]	1.26	0.10	1.08	1.47	0.003
***High-risk* Profile | Respiratory Disorders**					
Model 1: *Unadjusted*	2.64	0.25	2.20	3.18	<0.001
Model 2: *Model 1 + demographics & genetics* [a]	2.34	0.22	1.94	2.82	<0.001
Model 3: *Model 2 + Fully Adjusted* [b]	1.99	0.19	1.64	2.40	<0.001
Model 2a: *Demographics*	2.34	0.22	1.95	2.82	<0.001
Model 2b: *Genetics*	2.64	0.25	2.19	3.18	<0.001
Model 3a: Model 3 + *Medication*	1.99	0.19	1.65	2.41	<0.001
Model 3b: Model 3 + *Physical Activity*	1.94	0.19	1.61	2.35	<0.001
Model 3c: Model 3 + *Body Mass Index*	2.04	0.20	1.68	2.48	<0.001
Model 3d: Model 3 + *Fully Adjusted* [c]	2.01	0.20	1.65	2.44	<0.001
Model 4a: Model 2 + *Health*	2.34	0.22	1.94	2.82	<0.001
Model 4b: Model 3 + *Health*	1.98	0.19	1.64	2.40	<0.001
Model 4c: Model 3 + *Fully Adjusted* [d]	2.01	0.20	1.66	2.44	<0.001

Notes: The *low-risk* group is the reference; HR = hazard ratio; SE = standard error; CI = confidence interval; *p* = significance value.

a *Demographic and genetic variables*: age; sex; 10 principal components (PCs); C-reactive Protein (CRP) polygenic score (PGS); White Blood Cell Counts (WBCC) PGS; Insulin Growth Factor-1 (IGF-1) PGS; Anxiety PGS; Depression PGS; Schizophrenia PGS; Insomnia PGS; Pain PGS.

b All variables: age; sex; 10 PCs; CRP PGS; WBCC PGS; IGF-1 PGS; PGS; Anxiety PGS; Depression PGS; Schizophrenia PGS; Insomnia PGS; Pain PGS; education; wealth; smoking status; alcohol consumption; mobility.

c Additional variables: medication, physical activity; BMI.

d Additional variables: health (i.e., chronic lung disease; coronary heart disease; abnormal heart rhythm; heart murmur; congestive heart failure; angina; hypertension; diabetes; cancer; Parkinson's; Alzheimer's; dementia; asthma; arthritis; osteoporosis; psychiatric disorder).

Table S9az. **Longitudinal associations between C-reactive protein and hospitalisation for diseases of the respiratory system (*n*=3,846)**

**Adjustments**	**CRP**				
	**HR**	**SE**	**95% CI**		***p***
**Respiratory Disorders**					
Model 1: *Unadjusted*	1.67	0.08	1.52	1.83	<0.001
Model 2: *Model 1 + demographics & genetics* [a]	1.56	0.08	1.42	1.72	<0.001
Model 3: *Model 2 + Fully Adjusted* [b]	1.43	0.07	1.30	1.58	<0.001
Model 2a: *Demographics*	1.56	0.08	1.42	1.71	<0.001
Model 2b: *Genetics*	1.68	0.08	1.53	1.84	<0.001
Model 3a: Model 3 + *Medication*	1.44	0.07	1.30	1.58	<0.001
Model 3b: Model 3 + *Physical Activity*	1.42	0.07	1.29	1.56	<0.001
Model 3c: Model 3 + *Body Mass Index*	1.47	0.08	1.33	1.62	<0.001
Model 3d: Model 3 + *Fully Adjusted* [c]	1.45	0.08	1.31	1.61	<0.001
Model 4a: Model 2 + *Health*	1.56	0.08	1.42	1.72	<0.001
Model 4b: Model 3 + *Health*	1.43	0.07	1.30	1.58	<0.001
Model 4c: Model 3 + *Fully Adjusted* [d]	1.45	0.08	1.31	1.61	<0.001

Notes: The *low-risk* group is the reference; HR = hazard ratio; SE = standard error; CI = confidence interval; *p* = significance value.

a *Demographic and genetic variables*: age; sex; 10 principal components (PCs); C-reactive Protein (CRP) polygenic score (PGS); White Blood Cell Counts (WBCC) PGS; Insulin Growth Factor-1 (IGF-1) PGS; Anxiety PGS; Depression PGS; Schizophrenia PGS; Insomnia PGS; Pain PGS.

b All variables: age; sex; 10 PCs; CRP PGS; WBCC PGS; IGF-1 PGS; PGS; Anxiety PGS; Depression PGS; Schizophrenia PGS; Insomnia PGS; Pain PGS; education; wealth; smoking status; alcohol consumption; mobility.

c Additional variables: medication, physical activity; BMI.

d Additional variables: health (i.e., chronic lung disease; coronary heart disease; abnormal heart rhythm; heart murmur; congestive heart failure; angina; hypertension; diabetes; cancer; Parkinson's; Alzheimer's; dementia; asthma; arthritis; osteoporosis; psychiatric disorder).

Table S9ba. **Longitudinal associations between fibrinogen and hospitalisation for diseases of the respiratory system (*n*=3,846)**

**Adjustments**	**Fb**				
	**HR**	**SE**	**95% CI**		***p***
**Respiratory Disorders**					
Model 1: *Unadjusted*	1.64	0.10	1.47	1.84	<0.001
Model 2: *Model 1 + demographics & genetics* [a]	1.48	0.09	1.31	1.66	<0.001
Model 3: *Model 2 + Fully Adjusted* [b]	1.30	0.08	1.15	1.46	<0.001
Model 2a: *Demographics*	1.52	0.09	1.35	1.71	<0.001
Model 2b: *Genetics*	1.62	0.09	1.44	1.81	<0.001
Model 3a: Model 3 + *Medication*	1.29	0.08	1.15	1.46	<0.001
Model 3b: Model 3 + *Physical Activity*	1.29	0.08	1.14	1.45	<0.001
Model 3c: Model 3 + *Body Mass Index*	1.30	0.08	1.15	1.47	<0.001
Model 3d: Model 3 + *Fully Adjusted* [c]	1.29	0.08	1.15	1.46	<0.001
Model 4a: Model 2 + *Health*	1.48	0.09	1.32	1.67	<0.001
Model 4b: Model 3 + *Health*	1.31	0.08	1.16	1.48	<0.001
Model 4c: Model 3 + *Fully Adjusted* [d]	1.31	0.08	1.16	1.48	<0.001

Notes: The *low-risk* group is the reference; HR = hazard ratio; SE = standard error; CI = confidence interval; *p* = significance value.

a *Demographic and genetic variables*: age; sex; 10 principal components (PCs); C-reactive Protein (CRP) polygenic score (PGS); White Blood Cell Counts (WBCC) PGS; Insulin Growth Factor-1 (IGF-1) PGS; Anxiety PGS; Depression PGS; Schizophrenia PGS; Insomnia PGS; Pain PGS.

b All variables: age; sex; 10 PCs; CRP PGS; WBCC PGS; IGF-1 PGS; PGS; Anxiety PGS; Depression PGS; Schizophrenia PGS; Insomnia PGS; Pain PGS; education; wealth; smoking status; alcohol consumption; mobility.

c Additional variables: medication, physical activity; BMI.

d Additional variables: health (i.e., chronic lung disease; coronary heart disease; abnormal heart rhythm; heart murmur; congestive heart failure; angina; hypertension; diabetes; cancer; Parkinson's; Alzheimer's; dementia; asthma; arthritis; osteoporosis; psychiatric disorder).

Table S9bb. **Longitudinal associations between white blood cell counts and hospitalisation for diseases of the respiratory system (*n*=3,846)**

Adjustments	WBCC				
	**HR**	**SE**	**95% CI**		***p***
**Respiratory Disorders**					
Model 1: *Unadjusted*	3.18	0.42	2.45	4.13	<0.001
Model 2: *Model 1 + demographics & genetics* [a]	3.02	0.41	2.32	3.94	<0.001
Model 3: *Model 2 + Fully Adjusted* [b]	2.18	0.31	1.65	2.87	<0.001
Model 2a: *Demographics*	2.82	0.38	2.17	3.67	<0.001
Model 2b: *Genetics*	3.24	0.43	2.50	4.20	<0.001
Model 3a: Model 3 + *Medication*	2.16	0.30	1.64	2.85	<0.001
Model 3b: Model 3 + *Physical Activity*	2.13	0.30	1.62	2.80	<0.001
Model 3c: Model 3 + *Body Mass Index*	2.19	0.31	1.66	2.89	<0.001
Model 3d: Model 3 + *Fully Adjusted* [c]	2.14	0.30	1.62	2.81	<0.001
Model 4a: Model 2 + *Health*	2.88	0.39	2.21	3.74	<0.001
Model 4b: Model 3 + *Health*	2.10	0.29	1.60	2.76	<0.001
Model 4c: Model 3 + *Fully Adjusted* [d]	2.07	0.29	1.58	2.72	<0.001

Notes: The *low-risk* group is the reference; HR = hazard ratio; SE = standard error; CI = confidence interval; *p* = significance value.

a *Demographic and genetic variables*: age; sex; 10 principal components (PCs); C-reactive Protein (CRP) polygenic score (PGS); White Blood Cell Counts (WBCC) PGS; Insulin Growth Factor-1 (IGF-1) PGS; Anxiety PGS; Depression PGS; Schizophrenia PGS; Insomnia PGS; Pain PGS.

b All variables: age; sex; 10 PCs; CRP PGS; WBCC PGS; IGF-1 PGS; PGS; Anxiety PGS; Depression PGS; Schizophrenia PGS; Insomnia PGS; Pain PGS; education; wealth; smoking status; alcohol consumption; mobility.

c Additional variables: medication, physical activity; BMI.

d Additional variables: health (i.e., chronic lung disease; coronary heart disease; abnormal heart rhythm; heart murmur; congestive heart failure; angina; hypertension; diabetes; cancer; Parkinson's; Alzheimer's; dementia; asthma; arthritis; osteoporosis; psychiatric disorder).

Table S9bc. **Longitudinal associations between insulin growth factor-1 and hospitalisation for diseases of the respiratory system (*n*=3,846)**

Adjustments	IGF-1				
	**HR**	**SE**	**95% CI**		***p***
**Respiratory Disorders**					
Model 1: *Unadjusted*	0.53	0.05	0.43	0.64	<0.001
Model 2: *Model 1 + demographics & genetics* [a]	0.73	0.08	0.60	0.89	0.002
Model 3: *Model 2 + Fully Adjusted* [b]	0.73	0.08	0.60	0.90	0.002
Model 2a: *Demographics*	0.75	0.08	0.62	0.92	0.006
Model 2b: *Genetics*	0.52	0.05	0.43	0.63	<0.001
Model 3a: Model 3 + *Medication*	0.73	0.08	0.60	0.90	0.002
Model 3b: Model 3 + *Physical Activity*	0.74	0.08	0.61	0.90	0.003
Model 3c: Model 3 + *Body Mass Index*	0.73	0.08	0.60	0.90	0.002
Model 3d: Model 3 + *Fully Adjusted* [c]	0.74	0.08	0.61	0.91	0.003
Model 4a: Model 2 + *Health*	0.74	0.08	0.60	0.90	0.003
Model 4b: Model 3 + *Health*	0.74	0.08	0.60	0.90	0.003
Model 4c: Model 3 + *Fully Adjusted* [d]	0.75	0.08	0.61	0.91	0.004

Notes: The *low-risk* group is the reference; HR = hazard ratio; SE = standard error; CI = confidence interval; *p* = significance value.

a *Demographic and genetic variables*: age; sex; 10 principal components (PCs); C-reactive Protein (CRP) polygenic score (PGS); White Blood Cell Counts (WBCC) PGS; Insulin Growth Factor-1 (IGF-1) PGS; Anxiety PGS; Depression PGS; Schizophrenia PGS; Insomnia PGS; Pain PGS.

b All variables: age; sex; 10 PCs; CRP PGS; WBCC PGS; IGF-1 PGS; PGS; Anxiety PGS; Depression PGS; Schizophrenia PGS; Insomnia PGS; Pain PGS; education; wealth; smoking status; alcohol consumption; mobility.

c Additional variables: medication, physical activity; BMI.

d Additional variables: health (i.e., chronic lung disease; coronary heart disease; abnormal heart rhythm; heart murmur; congestive heart failure; angina; hypertension; diabetes; cancer; Parkinson's; Alzheimer's; dementia; asthma; arthritis; osteoporosis; psychiatric disorder).

Table S9bd. **Longitudinal associations between immune and neuroendocrine profiles and hospitalisation for diseases of the skin and subcutaneous tissue (*n*=4,045)**

**Adjustments**	**Immune and Neuroendocrine Profiles**				
	**HR**	**SE**	**95% CI**		***p***
***Moderate-risk* Profile | Skin Disorders**					
Model 1: *Unadjusted*	1.22	0.12	1.00	1.48	0.048
Model 2: *Model 1 + demographics & genetics* [a]	1.07	0.11	0.88	1.31	0.491
Model 3: *Model 2 + Fully Adjusted* [b]	1.00	0.10	0.82	1.23	0.968
Model 2a: *Demographics*	1.08	0.11	0.89	1.31	0.454
Model 2b: *Genetics*	1.22	0.12	1.01	1.49	0.044
Model 3a: Model 3 + *Medication*	1.01	0.10	0.82	1.23	0.965
Model 3b: Model 3 + *Physical Activity*	0.99	0.10	0.81	1.22	0.946
Model 3c: Model 3 + *Body Mass Index*	0.97	0.10	0.79	1.19	0.773
Model 3d: Model 3 + *Fully Adjusted* [c]	0.96	0.10	0.78	1.19	0.721
Model 4a: Model 2 + *Health*	1.07	0.11	0.88	1.31	0.491
Model 4b: Model 3 + *Health*	1.00	0.10	0.82	1.23	0.988
Model 4c: Model 3 + *Fully Adjusted* [d]	0.96	0.10	0.78	1.18	0.710
***High-risk* Profile | Skin Disorders**					
Model 1: *Unadjusted*	1.49	0.21	1.13	1.96	0.005
Model 2: *Model 1 + demographics & genetics* [a]	1.33	0.19	1.00	1.76	0.047
Model 3: *Model 2 + Fully Adjusted* [b]	1.19	0.17	0.90	1.58	0.231
Model 2a: *Demographics*	1.33	0.19	1.01	1.76	0.043
Model 2b: *Genetics*	1.49	0.21	1.13	1.97	0.005
Model 3a: Model 3 + *Medication*	1.19	0.17	0.89	1.58	0.237
Model 3b: Model 3 + *Physical Activity*	1.17	0.17	0.88	1.55	0.292
Model 3c: Model 3 + *Body Mass Index*	1.15	0.17	0.86	1.53	0.361
Model 3d: Model 3 + *Fully Adjusted* [c]	1.12	0.17	0.84	1.50	0.434
Model 4a: Model 2 + *Health*	1.33	0.19	1.00	1.76	0.047
Model 4b: Model 3 + *Health*	1.19	0.17	0.89	1.58	0.236
Model 4c: Model 3 + *Fully Adjusted* [d]	1.12	0.17	0.84	1.50	0.437

Notes: The *low-risk* group is the reference; HR = hazard ratio; SE = standard error; CI = confidence interval; *p* = significance value.

a *Demographic and genetic variables*: age; sex; 10 principal components (PCs); C-reactive Protein (CRP) polygenic score (PGS); White Blood Cell Counts (WBCC) PGS; Insulin Growth Factor-1 (IGF-1) PGS; Anxiety PGS; Depression PGS; Schizophrenia PGS; Insomnia PGS; Pain PGS.

b All variables: age; sex; 10 PCs; CRP PGS; WBCC PGS; IGF-1 PGS; PGS; Anxiety PGS; Depression PGS; Schizophrenia PGS; Insomnia PGS; Pain PGS; education; wealth; smoking status; alcohol consumption; mobility.

c Additional variables: medication, physical activity; BMI.

d Additional variables: health (i.e., chronic lung disease; coronary heart disease; abnormal heart rhythm; heart murmur; congestive heart failure; angina; hypertension; diabetes; cancer; Parkinson's; Alzheimer's; dementia; asthma; arthritis; osteoporosis; psychiatric disorder).

Table S9be. **Longitudinal associations between C-reactive protein and hospitalisation for diseases of the skin and subcutaneous tissue (*n*=4,045)**

**Adjustments**	**CRP**				
	**HR**	**SE**	**95% CI**		***p***
**Skin Disorders**					
Model 1: *Unadjusted*	1.22	0.08	1.07	1.39	0.004
Model 2: *Model 1 + demographics & genetics* [a]	1.13	0.08	0.99	1.30	0.076
Model 3: *Model 2 + Fully Adjusted* [b]	1.07	0.08	0.93	1.23	0.355
Model 2a: *Demographics*	1.13	0.08	0.99	1.30	0.076
Model 2b: *Genetics*	1.22	0.08	1.07	1.40	0.003
Model 3a: Model 3 + *Medication*	1.07	0.08	0.93	1.23	0.361
Model 3b: Model 3 + *Physical Activity*	1.06	0.08	0.92	1.22	0.453
Model 3c: Model 3 + *Body Mass Index*	1.04	0.08	0.90	1.21	0.591
Model 3d: Model 3 + *Fully Adjusted* [c]	1.03	0.08	0.89	1.19	0.694
Model 4a: Model 2 + *Health*	1.13	0.08	0.99	1.30	0.076
Model 4b: Model 3 + *Health*	1.07	0.08	0.93	1.23	0.369
Model 4c: Model 3 + *Fully Adjusted* [d]	1.03	0.08	0.89	1.19	0.704

Notes: The *low-risk* group is the reference; HR = hazard ratio; SE = standard error; CI = confidence interval; *p* = significance value.

a *Demographic and genetic variables*: age; sex; 10 principal components (PCs); C-reactive Protein (CRP) polygenic score (PGS); White Blood Cell Counts (WBCC) PGS; Insulin Growth Factor-1 (IGF-1) PGS; Anxiety PGS; Depression PGS; Schizophrenia PGS; Insomnia PGS; Pain PGS.

b All variables: age; sex; 10 PCs; CRP PGS; WBCC PGS; IGF-1 PGS; PGS; Anxiety PGS; Depression PGS; Schizophrenia PGS; Insomnia PGS; Pain PGS; education; wealth; smoking status; alcohol consumption; mobility.

c Additional variables: medication, physical activity; BMI.

d Additional variables: health (i.e., chronic lung disease; coronary heart disease; abnormal heart rhythm; heart murmur; congestive heart failure; angina; hypertension; diabetes; cancer; Parkinson's; Alzheimer's; dementia; asthma; arthritis; osteoporosis; psychiatric disorder).

Table S9bf. **Longitudinal associations between fibrinogen and hospitalisation for diseases of the skin and subcutaneous tissue (*n*=4,045)**

**Adjustments**	**Fb**				
	**HR**	**SE**	**95% CI**		***p***
**Skin Disorders**					
Model 1: *Unadjusted*	1.17	0.10	1.00	1.38	0.053
Model 2: *Model 1 + demographics & genetics* [a]	1.07	0.09	0.91	1.27	0.418
Model 3: *Model 2 + Fully Adjusted* [b]	0.99	0.09	0.83	1.17	0.883
Model 2a: *Demographics*	1.07	0.09	0.91	1.27	0.404
Model 2b: *Genetics*	1.17	0.10	1.00	1.38	0.054
Model 3a: Model 3 + *Medication*	0.99	0.09	0.83	1.17	0.888
Model 3b: Model 3 + *Physical Activity*	0.98	0.09	0.83	1.17	0.849
Model 3c: Model 3 + *Body Mass Index*	0.97	0.09	0.81	1.15	0.720
Model 3d: Model 3 + *Fully Adjusted* [c]	0.97	0.09	0.82	1.15	0.716
Model 4a: Model 2 + *Health*	1.07	0.09	0.90	1.27	0.432
Model 4b: Model 3 + *Health*	0.99	0.09	0.83	1.17	0.884
Model 4c: Model 3 + *Fully Adjusted* [d]	0.97	0.09	0.82	1.15	0.718

Notes: The *low-risk* group is the reference; HR = hazard ratio; SE = standard error; CI = confidence interval; *p* = significance value.

a *Demographic and genetic variables*: age; sex; 10 principal components (PCs); C-reactive Protein (CRP) polygenic score (PGS); White Blood Cell Counts (WBCC) PGS; Insulin Growth Factor-1 (IGF-1) PGS; Anxiety PGS; Depression PGS; Schizophrenia PGS; Insomnia PGS; Pain PGS.

b All variables: age; sex; 10 PCs; CRP PGS; WBCC PGS; IGF-1 PGS; PGS; Anxiety PGS; Depression PGS; Schizophrenia PGS; Insomnia PGS; Pain PGS; education; wealth; smoking status; alcohol consumption; mobility.

c Additional variables: medication, physical activity; BMI.

d Additional variables: health (i.e., chronic lung disease; coronary heart disease; abnormal heart rhythm; heart murmur; congestive heart failure; angina; hypertension; diabetes; cancer; Parkinson's; Alzheimer's; dementia; asthma; arthritis; osteoporosis; psychiatric disorder).

Table S9bg. **Longitudinal associations between white blood cell counts and hospitalisation for diseases of the skin and subcutaneous tissue (*n*=4,045)**

**Adjustments**	**WBCC**				
	**HR**	**SE**	**95% CI**		***p***
**Skin Disorders**					
Model 1: *Unadjusted*	1.71	0.32	1.19	2.46	0.004
Model 2: *Model 1 + demographics & genetics* [a]	1.55	0.30	1.06	2.27	0.024
Model 3: *Model 2 + Fully Adjusted* [b]	1.25	0.25	0.84	1.85	0.275
Model 2a: *Demographics*	1.51	0.29	1.04	2.20	0.031
Model 2b: *Genetics*	1.75	0.33	1.21	2.52	0.003
Model 3a: Model 3 + *Medication*	1.24	0.25	0.84	1.85	0.279
Model 3b: Model 3 + *Physical Activity*	1.23	0.25	0.83	1.82	0.309
Model 3c: Model 3 + *Body Mass Index*	1.20	0.25	0.81	1.79	0.363
Model 3d: Model 3 + *Fully Adjusted* [c]	1.19	0.24	0.80	1.77	0.399
Model 4a: Model 2 + *Health*	1.53	0.30	1.05	2.24	0.028
Model 4b: Model 3 + *Health*	1.24	0.25	0.84	1.84	0.285
Model 4c: Model 3 + *Fully Adjusted* [d]	1.18	0.24	0.80	1.77	0.406

Notes: The *low-risk* group is the reference; HR = hazard ratio; SE = standard error; CI = confidence interval; *p* = significance value.

a *Demographic and genetic variables*: age; sex; 10 principal components (PCs); C-reactive Protein (CRP) polygenic score (PGS); White Blood Cell Counts (WBCC) PGS; Insulin Growth Factor-1 (IGF-1) PGS; Anxiety PGS; Depression PGS; Schizophrenia PGS; Insomnia PGS; Pain PGS.

b All variables: age; sex; 10 PCs; CRP PGS; WBCC PGS; IGF-1 PGS; PGS; Anxiety PGS; Depression PGS; Schizophrenia PGS; Insomnia PGS; Pain PGS; education; wealth; smoking status; alcohol consumption; mobility.

c Additional variables: medication, physical activity; BMI.

d Additional variables: health (i.e., chronic lung disease; coronary heart disease; abnormal heart rhythm; heart murmur; congestive heart failure; angina; hypertension; diabetes; cancer; Parkinson's; Alzheimer's; dementia; asthma; arthritis; osteoporosis; psychiatric disorder).

Table S9bh. **Longitudinal associations between insulin growth factor-1 and hospitalisation for diseases of the skin and subcutaneous tissue (*n*=4,045)**

**Adjustments**	**IGF-1**				
	**HR**	**SE**	**95% CI**		***p***
**Skin Disorders**					
Model 1: *Unadjusted*	0.71	0.10	0.54	0.93	0.012
Model 2: *Model 1 + demographics & genetics* [a]	0.94	0.14	0.71	1.24	0.665
Model 3: *Model 2 + Fully Adjusted* [b]	0.95	0.13	0.72	1.25	0.711
Model 2a: *Demographics*	0.95	0.14	0.72	1.25	0.705
Model 2b: *Genetics*	0.70	0.10	0.53	0.91	0.009
Model 3a: Model 3 + *Medication*	0.95	0.13	0.72	1.25	0.718
Model 3b: Model 3 + *Physical Activity*	0.95	0.13	0.72	1.26	0.736
Model 3c: Model 3 + *Body Mass Index*	0.96	0.13	0.73	1.26	0.761
Model 3d: Model 3 + *Fully Adjusted* [c]	0.96	0.13	0.73	1.26	0.761
Model 4a: Model 2 + *Health*	0.94	0.13	0.71	1.24	0.664
Model 4b: Model 3 + *Health*	0.95	0.13	0.72	1.25	0.710
Model 4c: Model 3 + *Fully Adjusted* [d]	0.96	0.13	0.73	1.26	0.761

Notes: The *low-risk* group is the reference; HR = hazard ratio; SE = standard error; CI = confidence interval; *p* = significance value.

a *Demographic and genetic variables*: age; sex; 10 principal components (PCs); C-reactive Protein (CRP) polygenic score (PGS); White Blood Cell Counts (WBCC) PGS; Insulin Growth Factor-1 (IGF-1) PGS; Anxiety PGS; Depression PGS; Schizophrenia PGS; Insomnia PGS; Pain PGS.

b All variables: age; sex; 10 PCs; CRP PGS; WBCC PGS; IGF-1 PGS; PGS; Anxiety PGS; Depression PGS; Schizophrenia PGS; Insomnia PGS; Pain PGS; education; wealth; smoking status; alcohol consumption; mobility.

c Additional variables: medication, physical activity; BMI.

d Additional variables: health (i.e., chronic lung disease; coronary heart disease; abnormal heart rhythm; heart murmur; congestive heart failure; angina; hypertension; diabetes; cancer; Parkinson's; Alzheimer's; dementia; asthma; arthritis; osteoporosis; psychiatric disorder).
